# Clinical syndromes linked to biallelic germline variants in *MCM8* and *MCM9*

**DOI:** 10.1016/j.xhgg.2025.100480

**Published:** 2025-07-18

**Authors:** Noah C. Helderman, Ting Yang, Claire Palles, Diantha Terlouw, Hailiang Mei, Ruben H.P. Vorderman, Davy Cats, Marcos Díaz-Gay, Marjolijn C.J. Jongmans, Ashwin Ramdien, Irma van de Beek, Thomas F. Eleveld, Andrew Green, Frederik J. Hes, Marry M. van den Heuvel-Eibrink, Annelore Van Der Kelen, Sabine Kliesch, Roland P. Kuiper, Inge M.M. Lakeman, Lisa E.E.L.O. Lashley, Leendert H.J. Looijenga, Manon S. Oud, Johanna Steingröver, Yardena Tenenbaum-Rakover, Carli M. Tops, Frank Tüttelmann, Richarda M. de Voer, Dineke Westra, Margot J. Wyrwoll, Mariano Golubicki, Marina Antelo, Laia Bonjoch, Mariona Terradas, Laura Valle, Ludmil B. Alexandrov, Hans Morreau, Tom van Wezel, Sergi Castellví-Bel, Yael Goldberg, Maartje Nielsen

**Affiliations:** 1 Department of Clinical Genetics, Leiden University Medical Center, Leiden, the Netherlands; 2 Department of Cellular and Molecular Medicine, University of California, San Diego, La Jolla, CA 92093, USA; 3 Department of Bioengineering, University of California, San Diego, La Jolla, CA 92093, USA; 4 Moores Cancer Center, University of California, San Diego, La Jolla, CA 92037, USA; 5 Institute of Cancer and Genomic Sciences, University of Birmingham, Birmingham, UK; 6 Department of Pathology, Leiden University Medical Center, Leiden, the Netherlands; 7 Sequencing Analysis Support Core, Department of Biomedical Data Sciences, Leiden University Medical Center, Leiden, the Netherlands; 8 Digital Genomics Group, Structural Biology Program, Spanish National Cancer Research Center (CNIO), 28029 Madrid, Spain; 9 Princess Maxima Center for Pediatric Oncology, Utrecht, the Netherlands; 10 Department of Genetics, University Medical Center Utrecht, University of Utrecht, Utrecht, the Netherlands; 11 Department of Clinical Genetics, The Netherlands Cancer Institute, Amsterdam, the Netherlands; 12 Department of Clinical Genetics, Children’s Health Ireland (CHI) at Crumlin, Dublin, Ireland; 13 Vrije Universiteit Brussel (VUB), Universitair Ziekenhuis Brussel (UZ Brussel), Clinical Sciences, Research Group Genetics, Reproduction, and Development, Centre for Medical Genetics, Brussels, Belgium; 14 Division of Childhealth, University Medical Center Utrecht, Utrecht, the Netherlands; 15 Centre of Reproductive Medicine and Andrology, Department of Clinical and Surgical Andrology, University Hospital Münster, Münster, Germany; 16 Department of Obstetrics and Gynecology, Leiden University Medical Center, Leiden, the Netherlands; 17 Department of Pathology, University Medical Center Utrecht, Utrecht, the Netherlands; 18 Department of Human Genetics, Donders Institute for Brain, Cognition, and Behaviour, Radboud University Medical Center, Nijmegen, the Netherlands; 19 Centre of Medical Genetics, Institute of Reproductive Genetics, University and University Hospital of Münster, Münster, Germany; 20 Pediatric Endocrinology, Clalit Health Services, Afula, Israel; The Ruth and Bruce Rappaport Faculty of Medicine, Technion, Haifa, Israel; 21 Department of Human Genetics, Radboud University Medical Center, Radboud Institute for Molecular Life Sciences, Nijmegen, the Netherlands; 22 Centre for Regenerative Medicine, Institute for Stem Cell Research, School of Biological Sciences, University of Edinburgh, Edinburgh, UK; 23 Oncology Section and Molecular Biology Laboratory, Hospital of Gastroenterology “Dr. C.B. Udaondo”, Buenos Aires, Argentina; 24 Gastroenterology, Fundació de Recerca Clínic Barcelona-Institut d’Investigacions Biomèdiques August Pi I Sunyer (FRCB-IDIBAPS), CIBEREHD, Universitat de Barcelona, Clínic Barcelona, Barcelona, Spain; 25 Hereditary Cancer Program, Catalan Institute of Oncology, Oncobell Program, IDIBELL, CIBERONC, Hospitalet de Llobregat, Barcelona, Spain; 26 Raphael Recanati Genetic Institute, Rabin Medical Center-Beilinson Hospital, Petah Tikva, Israel

**Keywords:** MCM8, MCM9, Polyposis, adenomatous polyps, inheritable tumor syndrome, early-onset colorectal cancer, gastric cancer, hypogonadism, infertility, primary ovarian insufficiency

## Abstract

*MCM8* and *MCM9* are newly proposed cancer predisposition genes, linked to polyposis and early-onset cancer, in addition to their previously established association with hypogonadism. Given the uncertain range of phenotypic manifestations and unclear cancer risk estimates, this study aimed to delineate the molecular and clinical characteristics of biallelic germline *MCM8/MCM9* variant carriers. We found significant enrichment of biallelic *MCM9* variants in individuals with colonic polyps (odds ratio [OR] 6.51, 95% confidence interval [CI] 1.24–34.11, *p* = 0.03), rectal polyps (OR 8.40, 95% CI 1.28–55.35, *p* = 0.03), and gastric cancer (OR 27.03, 95% CI 2.93–248.5; *p* = 0.004) in data from the 100000 Genomes Project, compared to controls. No similar enrichment was found for biallelic *MCM8* variants or in the 200000 UK Biobank. Likewise, in our case series, which included 26 *MCM8* and 28 *MCM9* variant carriers, we documented polyposis, gastric cancer, and early-onset colorectal cancer (CRC) in *MCM9* carriers but not in *MCM8* carriers. Moreover, our case series indicates that beyond hypogonadism, biallelic *MCM8* and *MCM9* variants are associated with early-onset germ cell tumors (occurring before age 15). Tumors from *MCM8/MCM9* variant carriers predominantly displayed clock-like mutational processes, without evidence of DNA repair deficiency-associated signatures. Collectively, our data indicate that biallelic *MCM9* variants are associated with polyposis, gastric cancer, and early-onset CRC, while both biallelic *MCM8* and *MCM9* variants are linked to hypogonadism and the early development of germ cell tumors. These findings underscore the importance of including *MCM8/MCM9* in diagnostic gene panels for certain clinical contexts and suggest that biallelic carriers may benefit from cancer surveillance.

## Teaser

Biallelic *MCM9* variants are associated with polyposis, gastric cancer, and early-onset colorectal cancer, while both biallelic *MCM8* and *MCM9* variants are linked to hypogonadism and the early development of germ cell tumors. These findings underscore the importance of including *MCM8/MCM9* in diagnostic gene panels for certain clinical contexts.

## Introduction

The identification of cancer predisposition syndromes plays a crucial role in preventing and surveilling malignancies at an early stage in affected individuals. Nevertheless, a significant proportion of familial cancer cases lack a clear explanation.^1^ This poses challenges in developing personalized surveillance programs and highlights the urgency of exploring and identifying novel cancer predisposition genes.

The minichromosome maintenance 8 homologous recombination repair factor (*MCM8*; NM_032485.6, ENST00000610722.4, OMIM: 608187) and minichromosome maintenance 9 homologous recombination repair factor (*MCM9*; NM_017696.3, ENST00000619706.5, OMIM: 610098) genes are two recently suggested cancer predisposition genes.^2^^,^^3^^,^^4^ The proteins encoded by these genes form a helicase hexameric complex that is likely involved in DNA replication and the initiation of DNA replication,^5^^,^^6^^,^^7^^,^^8^^,^^9^ meiosis,^7^^,^^10^^,^^11^^,^^12^^,^^13^ homologous recombination,^14^^,^^15^^,^^16^^,^^17^^,^^18^^,^^19^^,^^20^ and mismatch repair (MMR).^4^^,^^19^^,^^21^

Following their significant association with primary ovarian insufficiency (POI; HP:0008209),^2^^,^^3^^,^^4^^,^^22^^,^^23^^,^^24^^,^^25^^,^^26^^,^^27^^,^^28^^,^^29^^,^^30^^,^^31^^,^^32^^,^^33^^,^^34^^,^^35^^,^^36^^,^^37^^,^^38^^,^^39^^,^^40^ biallelic germline variants of *MCM8/MCM9* were first linked to cancer in several families with polyposis (HP:0200063) and early-onset colorectal cancer (CRC; HP:0003003).^2^^,^^3^^,^^4^ Subsequently, there have been reports of individuals with CRC carrying a monoallelic *MCM8/MCM9* variant,^2^^,^^3^^,^^4^ as well as reports describing mono- and biallelic germline *MCM8/MCM9* variants in individuals with other nonmalignant pathologies, including short stature (HP:0004322),^29^^,^^34^^,^^35^^,^^38^^,^^39^^,^^34^^,^^35^^,^^38^^,^^39^ delayed puberty (HP:0000823),^22^^,^^23^^,^^26^^,^^28^^,^^33^^,^^38^^,^^39^^,^^40^ hypothyroidism (HP:0000821),^22^^,^^28^ and absent or infantile uteri/ovaries.^22^^,^^23^^,^^26^^,^^27^,^29^^,^^31^^,^^33^,^35^^,^^37^^,^^38^^,^^39^^,^^40^

Due to the limited number of families with biallelic germline *MCM8/MCM9* variants described so far, the complete spectrum of phenotypic manifestations and accurate cancer risk estimates remains uncertain. As a result, the incorporation of the *MCM8/MCM9* genes into diagnostic gene panels is not widespread, and the respective syndrome(s) associated with both genes could easily be missed. This study, therefore, sought to delineate the molecular and clinical features of biallelic germline *MCM8/MCM9* variants and to establish recommendations for the clinical management of variant carriers.

## Subjects, material, and methods

### Ethics statement

This study was approved by the local institutional review board (IRB) and biobank committee of the Leiden University Medical Center in the Netherlands (protocol B18.007). Storage and management of clinical and molecular data and participant samples from our case series were supervised by the Leiden University Medical Center. Participant samples were handled according to the medical ethical guidelines described in the code of conduct for responsible use of human tissue in the context of health research (Federation of Dutch Medical Scientific Societies). Samples were coded/anonymized, and all individuals provided written informed consent for the use of tissue and data.

### Population-based cohorts

#### Estimation of population allele and biallelic carrier frequencies in gnomAD version 2.1.1

**Figure 1:**
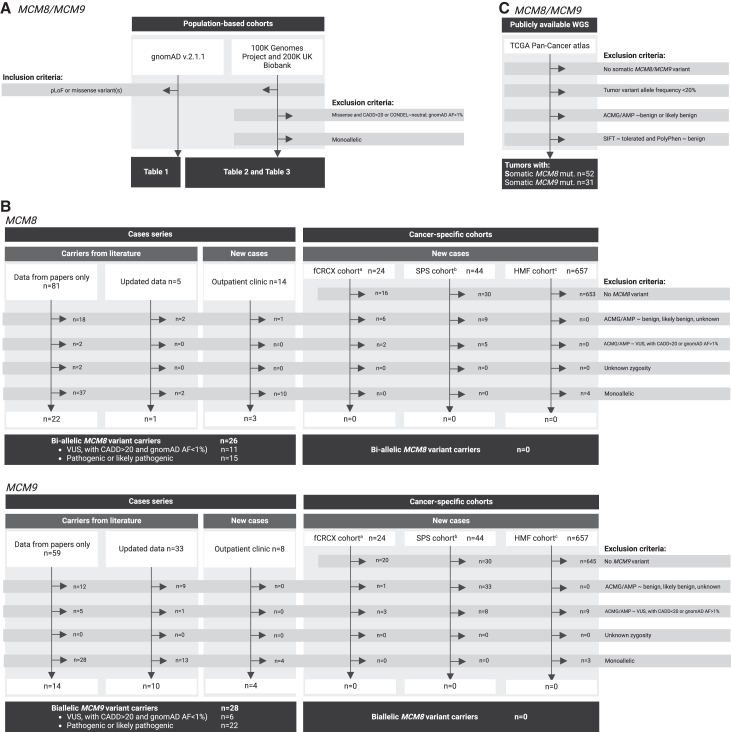
Flowchart of study approach Pathogenicity-based filtering of (A) population-based cohorts, (B) our case series and cancer-specific cohorts, and (C) TCGA Pan-Cancer atlas dataset. ^a^fCRCX cohort comprised 24 CRC-affected members of 16 Amsterdam-positive non-polyposis CRC families; ^b^SPS cohort comprised 44 unrelated serrated polyposis families; ^c^HMF cohort comprised 632 metastasized CRCs and 25 metastasized ECs. Tumors from TCGA Pan-Cancer Atlas were selected based on the presence of somatic *MCM8*/*MCM9* variants and are not related to germline variant carriers. ACMG/AMP, American College of Medical Genetics and Genomics; AF, allele frequency; CADD, Combined Annotation-Dependent Depletion; CRC, colorectal cancer; EC, endometrial cancer; pLoF, predicted loss of function; VUS, variant of uncertain significance.

The gnomAD version 2.1.1 database (https://gnomad.broadinstitute.org/), which comprises 125,748 exome sequences and 15,708 whole-genome sequences from a total of 141,456 unrelated individuals, was accessed in May 2023 to estimate the population allele frequencies (AFs) and biallelic carrier frequencies of *MCM8* and *MCM9* variants across diverse populations. We analyzed predicted loss of function (pLoF) variants—including splice acceptor, splice donor, frameshift, and stop gained variants—as well as missense variants, using variant annotations based on the Ensembl Variant Effect Predictor (VEP) classification (Figure 1)^41^ Population AFs were derived from the combined exome and genome dataset and expressed as the number of cases per 100,000 individuals, unless stated otherwise. Biallelic carrier frequencies were estimated using the gnomAD variant co-occurrence tool (https://gnomad.broadinstitute.org/variant-cooccurrence?dataset=gnomad_r2_1), which enables phasing of variants and is restricted to the exome dataset. Biallelic carriers were defined as individuals harboring either homozygous or compound heterozygous variants in *MCM8* or *MCM9*. For compound heterozygosity, only individuals with variants in *trans* (on different alleles) were included, while those with variants in *cis* (on the same allele) were excluded from the analysis.

#### Identification of carriers and variant enrichment analysis in 200000 UK Biobank and 100000 Genomes Project datasets

Germline variants in *MCM8* and *MCM9* were identified from the 100000 Genomes Project (project code 1142, version 17) and the 200000 exomes release of the UK Biobank (project code 86977, released on November 17, 2021). Variants were annotated using VEP version 107.^41^ We retained missense variants with a Combined Annotation-Dependent Depletion (CADD)^42^ score ≥20 and a deleterious Condel score,^43^ as well as pLoF variants (including splice acceptor, splice donor, frameshift, and stop gained variants), provided their AF was <1% in gnomAD version 2.1.1 (Figure 1). The impact of variants on the canonical transcripts was reported for *MCM8* (ENST00000610722.4) and *MCM9* (ENST00000619706.5).

The *International Classification of Diseases, 10th Revision* (ICD-10) codes from participants’ diagnosis information (Participant Explorer in 100000 Genomes Project, field ID 41270 in 200000 UK Biobank), along with the *International Classification of Diseases for Oncology* (ICD-O) codes obtained from cancer histology and behavior fields (field ID 40011 and 40012 in 200000 UK Biobank), as shown in Table S1, were searched to identify participants with phenotypes associated with *MCM8/MCM9* variants, as selected based on the literature^44^ as well as our case series.

We conducted case-control tests to assess whether potentially pathogenic biallelic (homozygous or compound heterozygous) *MCM8*/*MCM9* variants were enriched in participants with the phenotypes of interest compared to a control cohort. A total of 15,091 controls were identified from the 100000 Genomes Project dataset and 90,897 from the 200000 UK Biobank dataset. Controls were selected based on the absence of personal or family history of common cancers and any phenotypes listed in Table S1. To account for differences in age and ethnicity between cases and controls, association testing was performed using PLINK version 1.9, adjusting for both variables. Sex was included as a covariate in all analyses, except for breast cancer, endometrial cancer, and female infertility, where only female controls were considered.

### Case series

First, we identified *MCM8/MCM9* variant carriers through multiple channels. On August 1, 2023, we conducted a comprehensive literature search for “MCM8” and “MCM9” in the NCBI PubMed database. This strategy yielded 116 studies discussing *MCM8* and 75 studies discussing *MCM9*. We included all studies in English and carefully examined them for any descriptions of *MCM8/MCM9* variant carriers. We excluded (systematic) reviews to avoid duplicate participant data. Participant data were sourced from the papers themselves^22^^,^^23^^,^^24^^,^^25^^,^^26^^,^^27^^,^^28^^,^^29^^,^^30^^,^^31^^,^^32^^,^^34^^,^^35^^,^^36^^,^^37^^,^^38^^,^^39^^,^^40^^,^^45^^,^^46^^,^^47^^,^^48^^,^^49^^,^^50^ or updated data were obtained from the first or corresponding authors upon request.^2^^,^^3^^,^^4^^,^^51^

Second, as part of the European Reference Network for all participants with one of the rare genetic tumor risk syndromes (GENTURIS) initiative,^52^ we identified *MCM8/MCM9* variant carriers not previously documented through outpatient clinics at various institutes across Europe. Participant data were sourced from genetic practitioners or retrieved from health records.

#### Pathogenicity-based filtering and classification of the identified *MCM8/MCM9* variant carriers

The *MCM8/MCM9* variants identified in the case series and cancer-specific cohorts (see next section) were filtered based on their predicted pathogenicity (Figure 1). Initially, we annotated the *MCM8/MCM9* variants using the guidelines from the American College of Medical Genetics and Genomics (ACMG) and the Association for Molecular Pathology (AMP) for variant interpretation,^53^^,^^54^ along with the CADD scoring^42^ and gnomAD version 2.1.1 AF, accessed through Franklin.^55^ We excluded from the analysis (1) carriers of benign or likely benign *MCM8/MCM9* variants and (2) variants of uncertain significance (VUS) with a CADD score <20 or a gnomAD AF higher than 1%.

Individuals with homozygous or compound heterozygous variants that met the pathogenicity-based filtering criteria were considered to be biallelic carriers. Conversely, compound heterozygous carriers with one variant meeting the criteria and one that did not were categorized as monoallelic carriers. Additionally, compound heterozygous carriers with a pathogenic or likely pathogenic variant and a VUS that met the pathogenicity-based filtering criteria were included in the pathogenic or likely pathogenic group (i.e., as biallelic carriers).

### Cancer-specific cohorts

We ascertained several cancer-specific cohorts to search for variant carriers with cancer phenotypes associated with germline *MCM8/MCM9* variants. These included 44 non-related serrated polyposis patients (SPS cohort)^56^ and 24 cancer-affected members of 16 nonpolyposis CRC families (fCRCX cohort) from which germline whole-exome sequencing (WES) data was available for the analysis of single-nucleotide variants (SNVs) and insertion or deletion (indel) mutations, as well as 632 metastasized CRCs and 25 metastasized endometrial carcinomas with available germline and tumor whole-genome sequencing (WGS) data, accessible upon request by the Hartwig Medical Foundation database (reference no. HMF-DR-288; https://www.hartwigmedicalfoundation.nl/). The identified *MCM8/MCM*9 variant carriers were filtered and classified based on pathogenicity using the same criteria applied to the *MCM8/MCM9* variant carriers from the case series, as detailed in the previous section (Figure 1).

### Tumor DNA analysis

#### DNA sequencing and bioinformatic analysis of tumors from the case series

##### Participants and samples

To explore single-base substitution (SBS) mutational signatures potentially associated with *MCM8/MCM9* deficiency, DNA was obtained from formalin-fixed paraffin-embedded (FFPE) tumor tissue from the following individuals of our case series: 1 individual (1 tumor) with biallelic *MCM8* variants, 2 individuals (5 tumors) with monoallelic *MCM8* variants, 3 individuals (8 tumors) with biallelic *MCM9* variants, and 1 individual (1 tumor) with monoallelic *MCM8* and *MCM9* variants.

##### Sample preparation and molecular evaluation

DNA extraction from FFPE tissue blocks was conducted using the NucleoSpin DNA FFPE XS kit (Machery-Nagel, Düren, Germany), and DNA concentrations were quantified using the Qubit Meter dsDNA High Sensitivity kit (Thermo Fisher Scientific, Waltham, MA). WGS or WES was performed specifically for the purpose of this study using the NovaSeq 6000 Sequencing System (Illumina, San Diego, CA).

##### Somatic mutation calling

FASTQ files were aligned to the human genome build GRCh38.d1.vd1 using the Burrows-Wheeler Aligner (BWA-MEM, version 0.7.17).^57^ Picard MarkDuplicates (GATK version 4.1.4.1) (Picard Toolkit, http://broadinstitute.github.io/picard; Broad Institute, Cambridge, MA) was applied to mark all duplicated reads.^58^ SBS were identified using Mutect2 (GATK version 4.1.4.1),^59^ VarScan (version 2.4.3),^60^ MuSE (version 1.0),^61^ and Strelka (version 2.9.10)^62^ and filtered by variant caller confidences scores. Only variants that were called from at least two of these four callers were selected for the following mutational signature analysis and additional filtering based on their mutation confidence scores was applied: tumor logarithm of the odds score ≥ 10 (Mutect2) and SomaticEVS ≥ 13 (Strelka2). Samples with no matched germline sequencing data (10 out of 15 samples) were applied only to Mutect2 for variant calling under tumor-only mode.

##### Driver mutation identification

To identify potential driver mutations, we applied three complementary approaches to both SBSs and indels.

(1) We matched mutations to known driver events from The Cancer Genome Atlas (TCGA) MC3 study^63^ by aligning them based on protein position and amino acid change. To increase specificity, only mutations flagged in at least two of the following categories in the master driver mutation sheet for colon adenocarcinoma and rectal adenocarcinoma were retained: “New_Linear (cancer-focused) flag,” “New_Linear (functional) flag,” and “New_3D mutational hotspot flag.”
(2) We identified truncating mutations—nonsense, frameshift, or splice-site changes—in genes annotated as tumor suppressors, by cross-referencing the 82 IntOGen driver genes^64^ with the COSMIC Cancer Gene Census^65^ to determine tumor suppressor gene classification.
(3) We included missense mutations in any of the 82 IntOGen driver genes if they were annotated as “oncogenic” or “likely oncogenic” by OncoKB (version 3.4.1).

##### Mutational signature analysis

Mutational signature assignment was performed using SigProfilerAssignment (version 0.0.32)^66^ based on the COSMIC (version 3.3) SBS and small insertion and deletion (ID) reference signatures.^67^^,^^68^^,^^69^^,^^70^^,^^71^ Treatment-associated signatures (SBS11, SBS25, SBS31, SBS32, SBS35, SBS86, SBS87, SBS90, and SBS99) were excluded from all samples before signature assignment (using the *exclude_signature_subgroups* option), except for sample ID P8_33A, who had history of neoadjuvant chemotherapy treatment.

##### Tumor mutational burden

Tumor mutational burden (TMB) in coding regions was calculated by intersecting filtered VCF files with coding exonic regions defined by the Agilent GRCh38 exome capture kit (no_overlap_CCDS_CodingExons_33M.bed). The total number of somatic mutations within these regions was summed and normalized to the target region size (∼33 Mb) to yield TMB values expressed as the number of somatic mutations per megabase.

##### Total copy-number identification

Total copy-number analysis was performed using CNVkit (version 0.9.8)^72^ on both WGS and WES data, which were processed separately.

#### Bioinformatic analysis of publicly available WGS data

To further evaluate potential SBS mutational signatures associated with *MCM8/MCM9* deficiency, we analyzed tumor WGS data from two publicly available sources. First, we examined tumor data from cases with germline monoallelic *MCM8/MCM9* variants from the HMF cancer-specific cohort, as described earlier. Second, we evaluated tumor data from TCGA Pan-Cancer Atlas, accessed through cBioPortal for Cancer Genomics (https://www.cbioportal.org/) between February and April 2023.

For TCGA Pan-Cancer Atlas samples, tumors from any cancer type were selected based on the presence of somatic *MCM8/MCM9* variant(s) that met the following criteria: (1) a tumor variant AF of ≥20% and (2) classified as pathogenic or likely pathogenic or as a VUS according to the ACMG/AMP recommendations for variant interpretation.^53^^,^^54^ Additionally, variants were excluded in case they were assessed as tolerated by the Sorting Intolerant from Tolerant score^73^ and deemed benign by the PolyPhen score (Figure 1).^74^

In both the HMF and TCGA WGS datasets, SBS mutational signatures were identified by fitting the counts of SNVs per 96 tri-nucleotide context to the COSMIC version 3.3 reference mutational signatures^75^ using the MutationalPatterns tool.^76^

### Statistical analysis

Clinical data were collected using Castor Electronic Data Capture (https://castoredc.com). Figures were created, and statistical analysis was performed using RStudio version 2022.02.3+492 (Team R, Integrated Development for R, Boston, MA, 2022) or PLINK version 1.9.

## Results

### Population-based cohorts

#### Individuals with (biallelic) germline *MCM8/MCM9* variants are rare in gnomAD version 2.1.1

The occurrence of pLoF variants of *MCM8* in gnomAD (version 2.1.1) was 1.4 individuals per 100,000 persons across all populations, with the highest prevalence (5.5 individuals per 100,000 persons) in the African/African American population (Table 1). Regarding *MCM9*, the prevalence of a pLoF variant was 2.5 individuals per 100,000 persons across all populations, with the highest prevalence (5.7 individuals per 100,000 persons) found in the European Finnish population. The prevalence of missense *MCM8* and *MCM9* variants was 462.4 and 1,173.3 individuals per 100,000 persons, respectively. Twenty-three individuals (0.02%) were identified as biallelic carriers of missense variants or more severe mutations of the *MCM8* gene (Table 1). With respect to the *MCM9* gene, 22 (0.02%) individuals were predicted to be biallelic carriers, including 21 carriers of missense variants or worse and one carrier of a homozygous pLoF variant.

**Table 1:** Population allele and biallelic carrier frequencies in gnomAD version 2.1.1

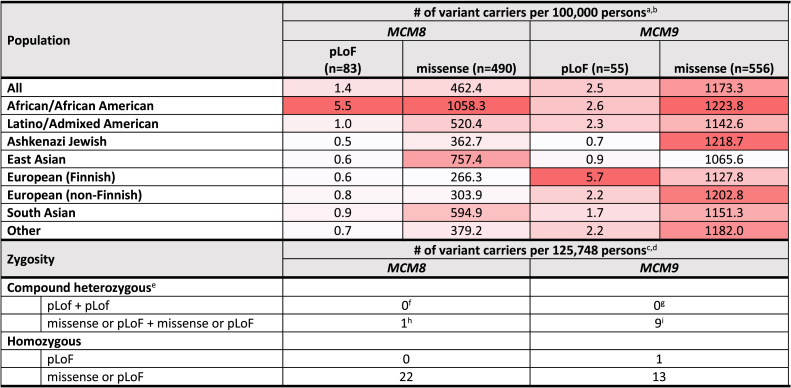

AF, allele frequency; gnomAD, Genome Aggregation Database; pLoF, predicted loss of function.

^a^Population AF of *MCM8/MCM9* variants calculated based on the gnomAD (version 2.1.1) database, accessed through https://gnomad.broadinstitute.org/ in May 2023.

^b^Color intensity of each cell is proportional to the population AF, in relation to the population AFs of cells from the same column.

^c^Data were extracted from the gnomAD version 2.1.1 database and were based on exomes only (*n* = 125,748). The gnomAD database was accessed through https://gnomad.broadinstitute.org/ in May 2023.

^d^Although highly uncommon, there is a possibility that an individual may be categorized in both the compound heterozygous group and the homozygous group. This situation arises when the individual carries a rare homozygous variant and simultaneously a rare heterozygous/heterozygous variant pair in the same gene.

^e^Only variants in *trans* (located on different copies of the gene) were considered.

^f^One individual had two unphased (unknown whether *cis* or *trans*) heterozygous variants.

^g^One individual had two unphased (unknown whether *cis* or *trans*) heterozygous variants.

^h^Ten individuals had two unphased (unknown whether *cis* or *trans*) heterozygous variants.

^i^Sixteen individuals had two unphased (unknown whether *cis* or *trans*) heterozygous variants.

#### Biallelic *MCM9* variant carriers in the 100000 Genomes Project have an increased risk of polyposis and gastric cancer, while no enrichment was observed for biallelic *MCM8* variants or in the 200000 UK Biobank dataset

In the 100000 Genomes Project, we identified 51 biallelic carriers (21 homozygous and 30 compound heterozygous) and 2,782 monoallelic carriers of pLoF or predicted deleterious missense variants in the *MCM8* gene. Moreover, we found 64 biallelic carriers (21 homozygous and 43 compound heterozygous) and 3,166 monoallelic carriers of pLoF or predicted deleterious missense variants in the *MCM9* gene. Among the 51 biallelic *MCM8* variant carriers in the 100000 Genomes Project, 2 individuals (3.9%) had CRC, 3 (5.9%) had colonic polyps, 3 (5.9%) had colonic adenomas, 3 (5.9%) had rectal polyps, 2 (3.9%) had hypothyroidism, and 5 (9.8%) had breast cancer. Additionally, 1 individual (2.0%) had epilepsy, 1 had endometrial cancer, 1 had short stature, and 1 experienced delayed puberty. Among the 64 biallelic *MCM9* variant carriers in the 100000 Genomes Project, 3 individuals (4.7%) had CRC, 2 (3.1%) had colonic polyps, 2 (3.1%) had colonic adenomas, 2 (3.1%) had rectal polyps, 3 (4.7%) had hypothyroidism, 5 (7.8%) had breast cancer, and 2 (3.1%) had epilepsy. Additionally, 1 individual (1.6%) had melanoma, 1 had gastric cancer, and 1 had endometrial cancer. While no significant enrichment of biallelic *MCM8* pLoF or predicted deleterious missense variants were observed for any of these phenotypes compared to controls, we did observe significant associations between biallelic *MCM9* pLoF or predicted deleterious missense variants and colonic polyps (odds ratio [OR] 6.51, 95% confidence interval [CI] 1.24–34.11, *p* = 0.03), rectal polyps (OR 8.40, 95% CI 1.28–55.35, *p* = 0.03), and gastric cancer (OR 27.03, 95% CI 2.93–248.5, *p* = 0.004) (Table 2).

**Table 2:** Enrichment analysis of biallelic *MCM8/MCM9* variants in 100000 Genomes Project, adjusting for age, sex, and ethnicity

Phenotype^a^	Potentially deleterious alleles in cases	Potentially deleterious alleles in controls	Non/unlikely deleterious alleles in cases	Non/unlikely deleterious alleles in controls	OR (95% CI)	*p*
***MCM8***						

Colonic	14	10	13,198	30,168	1.62 (0.38–6.86)	0.51
CRC	4	10	6,942	30,168	0.52 (0.04–6.08)	0.60
Colonic polyps	6	10	6,096	30,168	1.20 (0.20–7.11)	0.84
Colonic adenomas	6	10	5,748	30,168	2.37 (0.40–14.08)	0.34
Rectal polyps	6	10	2,802	30,168	2.41 (0.38–15.47)	0.35
Hypothyroidism	4	10	6,642	30,168	0.89 (0.14–5.83)	0.91
Breast cancer	10	6^b^	8,866	16,244^b^	1.04 (0.21–5.12)	0.96
Epilepsy	2	10	6,196	30,168	0.77 (0.03–19.72)	0.88
Endometrial cancer	2	6^b^	2,212	16,244^b^	0.83 (0.07–10.31)	0.89
Short stature	2	10	1,986	30,168	0.68 (3.32x10^−13^ to 1.42x10^12^)	0.98
Delayed puberty	2	10	256	30,168	1.19 (1.35x10^−37^ to 1.05x10^37^)	0.99

***MCM9***						

Colonic	12	10	13,200	30,168	3.68 (0.74–18.41)	0.11
CRC	6	10	6,940	30,168	1.49 (0.12–18.11)	0.75
Colonic polyps	6	10	6,096	30,168	**6.51 (1.24–34.11)**	**0.03**
Colonic adenomas	4	10	5,750	30,168	1.55 (0.13–18.33)	0.73
Rectal polyps	4	10	2,804	30,168	**8.40 (1.28–55.35)**	**0.03**
Hypothyroidism	6	10	6,640	30,168	3.88 (0.72–20.81)	0.11
Breast cancer	10	2^b^	8,866	16,242^b^	3.53 (0.33–37.2)	0.29
Epilepsy	4	10	6,194	30,168	0.40 (0.02–9.27)	0.57
Endometrial cancer	2	2^b^	2,212	16,242^b^	1.7 (0.01–270.4)	0.83
Melanoma	2	10	1,426	30,168	5.08 (0.43–59.06)	0.19
Gastric cancer	2	10	560	30,168	**27.03 (2.93–248.5)**	**0.004**

Boldface values indicate statistical significance. CI, confidence interval; CRC, colorectal cancer; OR, odds ratio.

a No cases with ovarian cancer, cervical cancer, female infertility, primary ovarian insufficiency, male infertility, absent or infantile uterus, or germ cell tumors were identified; as such, these phenotypes are not included in the table. Among the cases, short stature and delayed puberty were reported exclusively in biallelic *MCM8* carriers, while gastric cancer and melanoma were observed only in biallelic *MCM9* carriers.

b For the analysis of breast and endometrial cancers, only female controls were included for comparison.

In the 200,000 exomes release of the UK Biobank, we identified 110 biallelic carriers (47 homozygous and 63 compound heterozygous) and 8,453 monoallelic carriers of pLoF or predicted deleterious missense variants in the *MCM8* gene. Additionally, we found 74 biallelic carriers (15 homozygous and 59 compound heterozygous) and 4,991 monoallelic carriers of pLoF or predicted deleterious missense variants in the *MCM9* gene. Among the 110 biallelic *MCM8* variant carriers in the 200000 UK Biobank, 2 individuals (1.8%) were registered with CRC, 3 (2.7%) with colonic polyps, 4 (3.6%) with adenomas, 1 (0.9%) with female infertility, and 6 (5.5%) with hypothyroidism. Among the 74 biallelic *MCM9* variant carriers in the 200000 UK Biobank, 1 individual (1.4%) was registered with colorectal cancer (CRC), 3 (4%) with colonic polyps, 6 (8%) with adenomas, 1 (1.4%) with rectal polyps, and 2 (2.7%) with hypothyroidism. However, no significant enrichment of biallelic *MCM8/MCM9* pLoF or predicted deleterious missense variants was observed for any of these phenotypes compared to controls in the 200000 UK Biobank (Table 3).

**Table 3:** Enrichment analysis of biallelic *MCM8/MCM9* variants in 200000 UK Biobank, adjusting for age, sex, and ethnicity

Phenotype^a^	Potentially deleterious alleles in cases	Potentially deleterious alleles in controls	Non/unlikely deleterious alleles in cases	Non/unlikely deleterious alleles in controls	OR (95% CI)	*p*
***MCM8***						

Colonic	16	136	39,158	181,658	0.54 (0.26–1.14)	0.11
CRC	4	136	6,474	181,658	0.83 (0.20–3.41)	0.80
Colonic polyps	6	136	18,518	181,658	0.43 (0.14–1.39)	0.16
Colonic adenomas	8	136	20,586	181,658	0.52 (0.19–1.44)	0.21
Female infertility	2	74^b^	988	98,908^b^	2.68 (0.34–21.11)	0.35
Hypothyroidism	12	136	21,982	181,658	0.67 (0.29–1.58)	0.36

***MCM9***						

Colonic	16	84	39,158	181,710	-	-
CRC	2	84	6,476	181,710	0.82 (0.11–5.99)	0.84
Colonic polyps	6	84	18,518	181,710	0.80 (0.25–2.63)	0.72
Colonic adenomas	12	84	20,582	181,710	1.51 (0.63–3.61)	0.35
Rectal polyps	2	84	10,326	181,710	0.49 (0.07–3.59)	0.48
Hypothyroidism	4	84	21,990	181,710	0.46 (0.11–1.94)	0.29

CI, confidence interval; CRC, colorectal cancer; OR, odds ratio.

a No cases with breast cancer, gastric cancer, melanoma, endometrial cancer, ovarian cancer, cervical cancer, primary ovarian insufficiency, male infertility, epilepsy, short stature, delayed puberty, absent or infantile uterus, or germ cell tumors were identified; as such, these phenotypes are not included in the table. Among the cases, female infertility was reported exclusively in biallelic *MCM8* carriers, while rectal polyps were observed only in biallelic *MCM9* carriers.

b For the analysis of female infertility, only female controls were included for comparison.

None of the other phenotypes investigated (see Table S1) were registered among the biallelic *MCM8/MCM9* variant carriers, based on ICD-10/ICD-O registrations.

### Case series

#### Phenotype of biallelic germline *MCM8/MCM9* variants carriers

In our case series, we identified 26 biallelic *MCM8* variant carriers (including 15 with pathogenic or likely pathogenic variants and 11 with a VUS) and 28 biallelic *MCM9* variant carriers (including 22 with pathogenic or likely pathogenic variants and 6 with a VUS) that met the pathogenicity-based filtering criteria. This group included 3 biallelic *MCM8* and 4 biallelic *MCM9* variant carriers who had not been previously described (Figure 1). An overview of all identified *MCM8/MCM9* variant carriers, including their sources, is presented in Table S2. The supplemental information contain a detailed description of all newly identified *MCM8/MCM9* variant carriers and previously documented carriers for whom we obtained updated clinical information (individuals meeting the pathogenicity-based filtering criteria only), with the pedigrees being presented in Figure S1.

##### Biallelic *MCM8/MCM9* variant carriers often present with hypogonadism linked to impaired gonadal development

**Figure 2:**
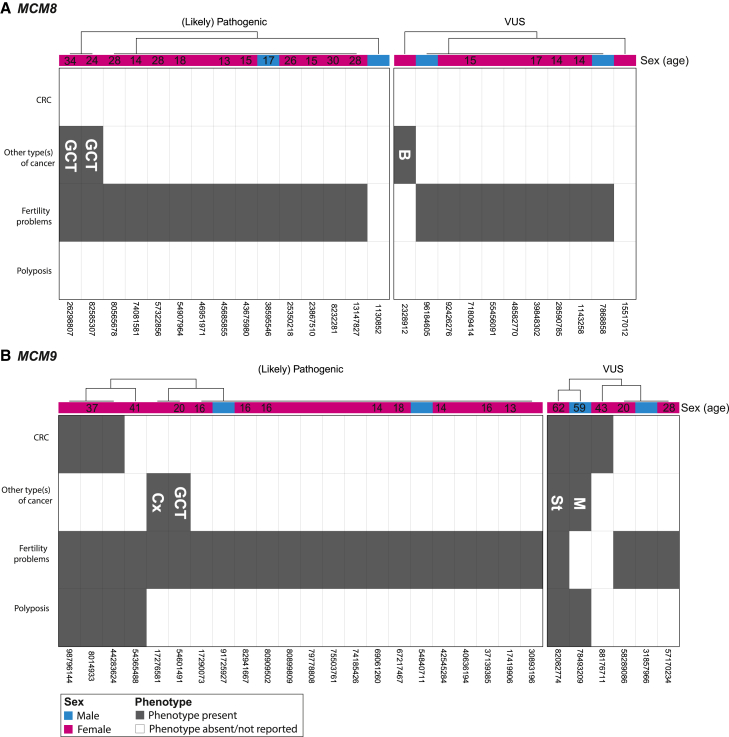
Phenotype of biallelic *MCM8/MCM9* variant carriers The phenotype is presented for all (A) biallelic *MCM8* and (B) biallelic *MCM9* variant carriers from our case series. Each column represents an individual, while each row corresponds to one of the four primary observed phenotypes: CRC, other type(s) of cancer, hypogonadism, and polyposis. Person IDs are provided below each column, whereas their corresponding ages, which represent the most recent reported age of each individual, are shown above every column (when available). B, breast cancer; Cx, cervical cancer; GCT, germ cell tumor; M, melanoma; St, stomach cancer.

**Figure 3:**
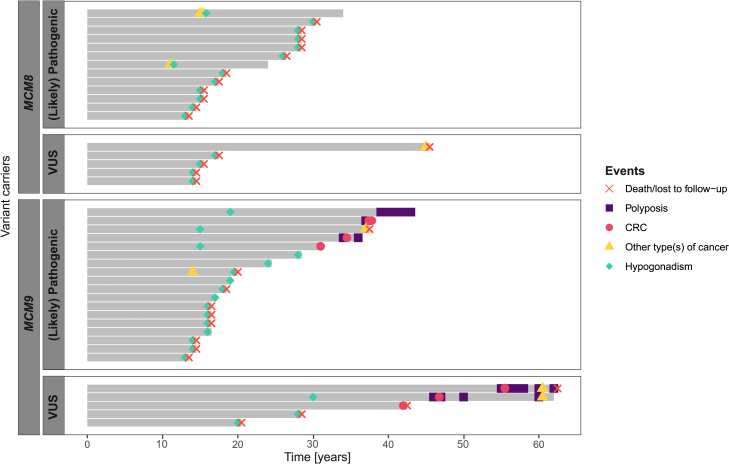
Disease onset in biallelic *MCM8/MCM9* variant carriers The onset of the four primary observed phenotypes (CRC, other type[s] of cancer, hypogonadism, and polyposis) is displayed for each biallelic *MCM8/MCM9* variant carrier with available age details in our case series. Those without age details were excluded from the analysis. Individuals are ordered by ACMG/AMP classification (pathogenic or likely pathogenic, VUS)^53^^,^^54^ and current age or age at the time of death/lost to follow-up.

The majority of individuals with biallelic *MCM8* (23 out of 26, 88%) or *MCM9* (26 out of 28, 93%) variants from our case series experienced hypogonadism (HP:0000815) (Figure 2). Apart from five males (three with biallelic *MCM8* variants and two with biallelic *MCM9* variants) with azoospermia (no sperm in the semen; HP:0000027), these issues involved women affected by POI. Fourteen out of 20 (70%) individuals affected by POI and carrying biallelic *MCM8* variants had undetectable or small ovaries coupled with an infantile or absent uterus upon ultrasound in 13 (65%) of the affected individuals. Among the biallelic *MCM9* variant carriers affected by POI, 14 out of 23 (61%) exhibited invisible or small ovaries, and 12 out of 23 (52%) had infantile or absent uteri. Furthermore, osteoporosis or delayed bone age (HP:0000939) was reported in seven individuals with biallelic *MCM9* variants and one individual with biallelic *MCM8* variants, all of whom were affected by hypogonadism. In both the *MCM8* and *MCM9* groups, hypogonadism manifested at a relatively young age, typically between 10 and 30 years (Figure 3). Many of these individuals were part of earlier studies, with no updated clinical data available upon request, so most were lost to follow-up post-publication.

##### Biallelic *MCM9* variant carriers may face polyposis, gastric cancer, and early-onset CRC, while both biallelic *MCM8/MCM9* carriers may face female germ cell tumors

Polyposis (typically >20 polyps, including hyperplastic, adenomatous, and serrated types) was reported in 6 out of 28 (21%) biallelic *MCM9* variant carriers from our case series (Figure 2). Similarly, CRC was observed in 6 of 28 (21%) biallelic *MCM9* variant carriers in our case series. This includes three carriers of likely pathogenic variant(s) who developed CRC between the ages of 30 and 40 and three carriers with a VUS diagnosed between 40 and 60 years (Figure 3). No CRC or polyp diagnoses were reported among the biallelic *MCM8* variant carriers. Three female carriers—two with biallelic *MCM8* variants and one with a biallelic *MCM9* variant—were diagnosed with germ cell tumors (HP:0100728) between the ages of 11 and 15 years. These included two endodermal sinus tumors originating from dysgerminomas, which themselves arose from gonadoblastomas, and one germ cell tumor of unspecified origin. Single biallelic *MCM9* variant carriers were diagnosed with gastric cancer (HP:0012126), a human papillomavirus-unrelated clear cell carcinoma of the cervix (HP:0031522), and melanoma (HP:0012056), whereas a biallelic *MCM8* variant carrier was diagnosed with breast cancer (HP:0003002).

##### Monoallelic *MCM8/MCM9* variants may experience hypogonadism

During the pathogenicity-based filtering process of our case series, we filtered 49 monoallelic *MCM8* variant carriers and 45 monoallelic *MCM9* variant carriers. Out of these 49 monoallelic *MCM8* variant carriers, hypogonadism was noted in 14 (29%) individuals, with two having a likely pathogenic variant and 12 carrying a VUS (Figures S2 and S3). Two monoallelic *MCM8* variant carriers were diagnosed with CRC, another two with polyposis, and two individuals with a monoallelic *MCM8* variant were diagnosed with breast cancer.

Among the 45 monoallelic *MCM9* variant carriers from our case series, 10 (22%) were known to have hypogonadism, including 1 individual who was also diagnosed with CRC and polyposis (Figures S2 and S3). CRC and polyps were additionally reported in 5 and 6 other monoallelic *MCM9* variant carriers, respectively. No other types of cancer were reported in the monoallelic *MCM9* group.

#### Genotype-phenotype correlations reveal potential hotspot sites

**Figure 4:**
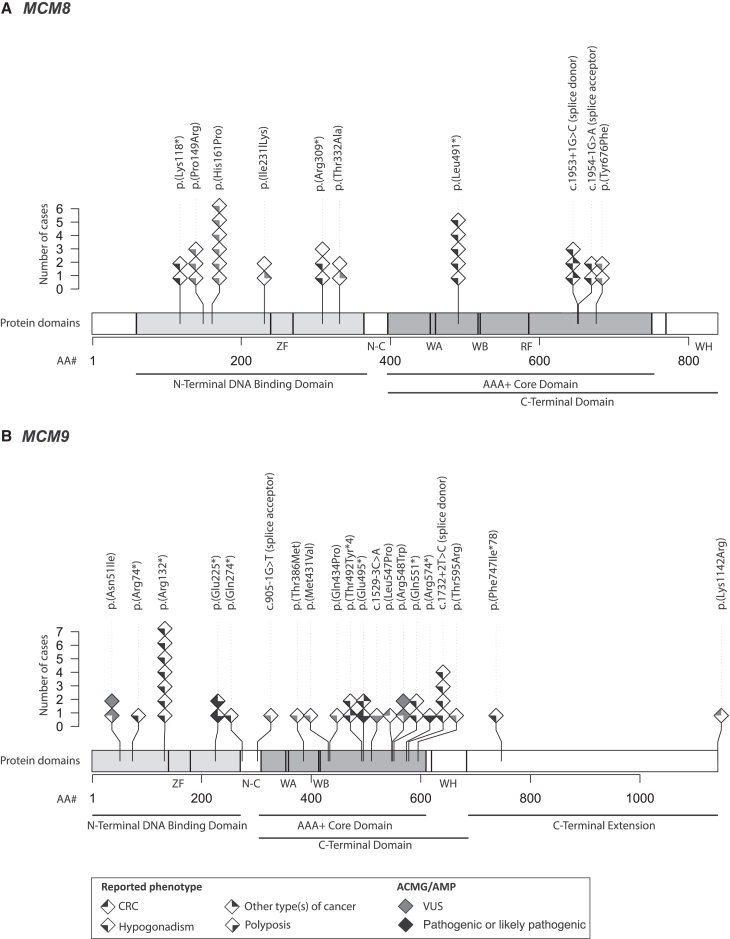
Biallelic *MCM8/MCM9* variants mapped onto the respective protein domains (A) *MCM8* and (B) *MCM9* variants from all biallelic variant carriers in our case series are mapped onto the domains of the MCM8 and MCM9 proteins, respectively. Each homozygote variant carrier corresponds to one diamond symbol, whereas for compound heterozygous variant carriers, both variants are separately plotted. The fill and color of the diamond symbols correspond to the phenotype of the individual (CRC, other type[s] of cancer, hypogonadism, polyposis) and the ACMG/AMP classification of the variant (pathogenic or likely pathogenic, VUS),^53^^,^^54^ respectively. N-C, N-C linker domain; RF, arginine finger; VUS, variant of uncertain significance; WA, Walker A; WB, Walker B; WH, winged-helix; ZF, zinc finger.

Mapping of variants onto the MCM8 and MCM9 protein domains revealed that the variants in our case series clustered in two key regions: the N-terminal DNA binding domain, which is crucial for protein-DNA binding (6 of 11 *MCM8* variants in biallelic carriers, 55%; 4 of 20 *MCM9* variants in biallelic carriers, 20%), and the AAA+ core domain, essential for DNA helicase activity (5 of 11 *MCM8* variants in biallelic carriers, 45%; 12 of 20 *MCM9* variants in biallelic carriers, 60%) (Figures 4 and S4).^44^ Additionally, several variants were found to be shared among multiple families with hypogonadism from our case series. For instance, the c.482A>C [p.(His161Pro)] VUS in the *MCM8* gene, previously linked to hypogonadism,^39^^,^^26^^,^^46^^,^^49^^,^^51^ was shared by six biallelic carriers across two families. Similarly, the pathogenic c.394C>T [p.(Arg132∗)] variant in the *MCM9* gene, also associated with hypogonadism,^39^^,^^49^^,^^51^ was shared by seven biallelic carriers from four unrelated families.

### Cancer-specific cohorts

No biallelic *MCM8/MCM9* variant carriers meeting the pathogenicity-based filtering criteria were identified in the SPS case group, fCRCX, and HMF (metastasized CRC and endometrial cancer case groups) cancer-specific cohorts. In the HMF cancer-specific cohort, four monoallelic *MCM8* and three monoallelic *MCM9* variant carriers meeting the pathogenicity-based filtering criteria were identified with CRC.

### Tumor DNA analysis

**Figure 5:**
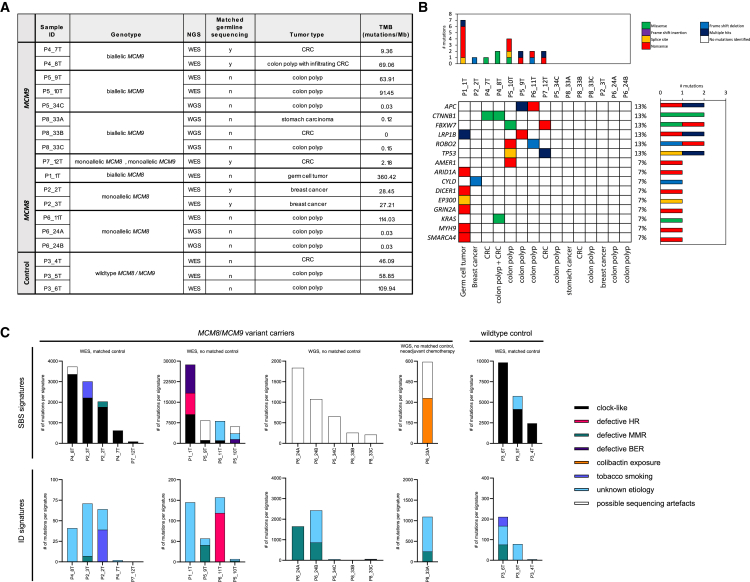
Mutational landscape of tumors from *MCM8/MCM9* variant carriers from our case series (A) Sample overview of the tumors that were available from our case series for mutational signature analysis, including the corresponding genotype, next-generation sequencing (NGS) approach, the availability of normal control tissue, and the tumor type. Control tissue originated from an individual who tested negative for germline *MCM8/MCM9* variants. TMB was defined as the number of somatic mutations per megabase. For WGS samples, only somatic mutations located within coding exonic regions were included in the TMB calculation, unlike in the mutational signature analyses, where all somatic mutations were considered. Of note, TMB values derived from WGS samples were lower than those from WES samples. This difference may reflect factors such as WGS sample contamination leading to the exclusion of true variants by variant callers, differences in sequencing depth and coverage, or underlying biological differences between the samples. (B) Oncoplot visualizing the detected driver mutations for every tumor. (C) The number of mutations in each signature is presented for every tumor. Mutational signature assignment was performed using SigProfilerAssignment (version 0.0.32)^66^ based on the COSMIC version 3.3 single-base substitution (SBS) and insertion and deletion (ID) reference signatures. SBS1 and SBS5 were classified as clock-like mutational signatures. SBS3 and ID6 were considered to be caused by defective homologous repair (HR). SBS26, ID1, and ID2 were linked to defective MMR, and SBS30 and SBS36 were associated with defective base excision repair (BER). SBS88 was attributed to colibactin exposure, and SBS92 and ID3 were attributed to tobacco smoking. SBS37, SBS40, SBS94, ID4, ID5, ID9, ID10, ID11, ID14, ID15, and ID16 were considered to be of unknown etiology, while SBS40, SBS45, SBS50, SBS51, SBS54, SBS56, SBS58, and SBS95 were considered possible sequencing artifacts. ID, insertion and deletion; MMR, mismatch repair; TMB, tumor mutational burden; WES, whole-exome sequencing; WGS, whole-genome sequencing.

An overview of the analyzed tumors from the case series is provided in Figure 5A. Germline WES-based DNA analysis, performed using previously described methods,^77^ revealed no pathogenic variants in other well-established CRC- or polyposis-associated genes in any of the corresponding participants. Of note, one participant who had three polyps included in the analysis (P6_11T, P6_24A, and P6_24B) carried biallelic VUS in *HROB*, which encodes a protein believed to support the function of *MCM8* and *MCM9*.^9^^,^^78^^,^^79^

#### Most tumors in the case series appear diploid; driver mutations were identified in a subset of samples

Copy-number analysis showed that most tumors in the case series were diploid, with no significant gains or losses in CRC-related genes (Figure S5). TMB ranged from less than 1 to 360.42 mutations per megabase, with a median of 27.83 mutations per megabase and an interquartile range of 0.13–27.83. Two tumors—P6_24B and P8_33B—exhibited highly fragmented copy-number profiles, fluctuating between values of 1 and 3. However, these results should be interpreted with caution due to low sequencing depth, which may have limited the ability of CNVkit to accurately assign copy-number states. Driver mutations in CRC-related genes were detected in a subset of samples (Figure 5B), with detailed information on the specific mutations provided in Table S3.

#### Clock-like and unknown-etiology signatures dominate tumors from the case series and HMF cancer-specific cohort, while MMR and HR deficiency-associated signatures appear in only a minority of cases

SBS1 and SBS5, which reflect clock-like mutational processes,^80^ were detected in all tumors from our case series with matched germline sequencing data, as well as in one CRC and two polyps from a wild-type control (Figure 5C). In addition, SBS1 and SBS5—alongside SBS93 and SBS40, both of unknown origin—were the most prominent signatures in metastasized CRCs from seven individuals with monoallelic *MCM8* or *MCM9* variants in the HMF cancer-specific cohort (Figure S6).

Tumors from our case series lacking matched germline sequencing data were dominated by sequencing artifact signatures (SBS45, SBS47, SBS50, SBS51, SBS54, SBS56, SBS58, and SBS95), limiting our ability to compare these to tumors with matched controls or to previously published cases.^67^^,^^68^^,^^69^^,^^70^^,^^71^

Signatures ID1 or ID2, which display a high number of indels in MMR-deficient cases, were detected in 8 of 15 tumors from our case series and in 2 control tumors. SBS26, similarly associated with MMR deficiency, was identified in one tumor (P2_2T), where it contributed to a minority of the mutations (269 out of 2,026, 13%).

Signatures associated with homologous recombination (HR) deficiency, including SBS3 and ID6, were each identified in one tumor (P1_1T and P6_11T, respectively) from two separate participants, both of whom lacked matched germline data. In addition, ID signatures of unknown etiology, including ID4, ID5, ID9, ID10, ID11, ID14, ID15, and ID16, were detected in all but two tumors from our case series and in all three control tumors.

#### Somatic *MCM8/MCM9* mutations may occur as a result of other DNA repair deficiencies and mutational processes, potentially involving copy-number variations

In TCGA Pan-Cancer Atlas dataset, insights into the somatic mutational behavior of *MCM8* and *MCM9* were gained through the observation of copy-number alterations in both genes. Furthermore, unsupervised hierarchical clustering of SBS mutational signature profiles revealed clusters characterized by signatures such as SBS7a/b (UV damage), SBS2 and SBS13 (APOBEC activity), SBS6, SBS14, SBS15, SBS20, and SBS21 (MMR deficiency), and SBS10a/b (POLE deficiency),^67^^,^^68^^,^^69^^,^^70^^,^^71^ which suggest that somatic *MCM8*/*MCM9* variants may be secondary to other DNA repair deficiencies and mutational processes (Figure S7).

## Discussion

Following the initial discovery of biallelic germline *MCM8/MCM9* variants in families with CRC, polyposis, and hypogonadism,^2^^,^^3^^,^^4^ we present a comprehensive clinical and molecular characterization of biallelic *MCM8/MCM9* variant carriers from multiple sources. Our analysis of the 100000 Genomes Project reveals that biallelic *MCM9* variant carriers are at increased risk for polyposis and gastric cancer, a pattern not observed in biallelic *MCM8* carriers. This finding is further supported by our case series, which included 26 biallelic *MCM8* and 28 biallelic *MCM9* variant carriers, including 7 previously unreported cases. Furthermore, the case series indicates that in addition to the previously established association with hypogonadism due to impaired gonadal development, biallelic *MCM8* and *MCM9* variants are linked to the development of germ cell tumors, with biallelic *MCM9* variants potentially associated with early-onset CRC. These findings highlight the importance of including *MCM8* and *MCM9* in diagnostic gene panels for relevant clinical contexts and suggest that biallelic carriers may benefit from cancer surveillance.

Gaining an unbiased understanding of the phenotype of biallelic *MCM8/MCM9* variant carriers is currently challenging. This difficulty arises mainly from the limited inclusion of *MCM8/MCM9* genes in current diagnostic gene panels for cancer and polyposis, constraining our case series, and the relative rarity of germline *MCM8/MCM9* variants in the general population, as reflected by our investigations in gnomAD version 2.1.1, the 100000 Genomes Project, and the 200,000 exome release of the UK Biobank. This rarity may have contributed to the absence of biallelic *MCM8/MCM9* variants in the cancer-specific cohorts and could have influenced the enrichment analysis of these variants in the 100000 Genomes Project and 200000 UK Biobank. Aside from the increased risk of polyposis and gastric cancer associated with biallelic *MCM9* variants in the 100000 Genomes Project, the lack of enrichment for biallelic *MCM8/MCM9* variants in other phenotypes and in the 200000 UK Biobank may be attributed to one of two factors: (1) these variants may not actually contribute to studied phenotypes, or (2) there may be limitations in the analysis itself, such as reliance on the accuracy and consistency of ICD-10/ICD-O registrations and the variant filtering approach, which, partly due to the relative novelty of both genes, relied primarily on *in silico* prediction tools. In regard to our case series, we acknowledge an ascertainment bias, contributing to the high frequency of hypogonadism in our cohort since most individuals examined were from studies primarily focused on fertility problems rather than cancer. In contrast, the occurrence of cancer and polyposis among biallelic *MCM8/MCM9* variant carriers may be underestimated because many individuals in our case series are still young, potentially too young to have developed cancer, and because colonoscopies are not typically recommended for biallelic *MCM8/MCM9* variant carriers. Moreover, the prevalence of the associated phenotypes might be underestimated due to our variant filtering approach, being dependent on limited *in silico* prediction algorithms and data from previous studies, for instance in regard to segregation analysis and variant phasing. This may have led to misclassification of individuals as (biallelic) variant carriers, thereby potentially diluting the observed prevalence of phenotypes in our analyses.

Despite its limitations, our population-based analysis and case series describe the most extensive collection of individuals with biallelic *MCM8/MCM9* variants to date, underscoring the importance of considering these variants in specific clinical contexts. We recommend considering biallelic *MCM9* variants in individuals and families with unexplained polyposis, gastric cancer, germ cell tumors, or (early-onset) CRC, particularly in cases of recessive inheritance and known hypogonadism, until more data are available. Similarly, biallelic *MCM8* variants should be considered in cases of unexplained germ cell tumors, especially when accompanied by recessive inheritance or hypogonadism. Additionally, given previous reports linking biallelic *MCM8* variants to CRC^4^ and the potential underestimation of cancer and polyposis in our case series, it may be prudent to consider biallelic *MCM8* variants in cases of unexplained CRC or polyposis until further data are available. As these genes become more integrated into diagnostic gene panels and more families are identified, larger sample sizes and longer follow-up periods will allow for more accurate cancer risk assessments.

Given the range of malignancies observed in our case series, surveillance for these individuals could be considered within a shared decision-making framework, taking into account the current evidence until more data become available. Similar to the *NTHL1*- and *MUTYH*-deficiency syndromes,^81^^,^^82^^,^^83^ which are associated with CRC and polyposis, the *MCM9*-deficiency syndrome observed in our population-based analysis and case series may warrant comparable surveillance protocols. Established colon surveillance guidelines for *NTHL1*- and *MUTYH*-deficiency syndromes,^81^^,^^82^^,^^83^ which recommend (bi)annual colonoscopy beginning around 18–20 years of age, could potentially be extended to individuals carrying biallelic *MCM9* variants. However, given the observed onset age of 30–60 years in our series, initiating colonoscopy at 25 years may be more appropriate. Additionally, due to the potential increased risk of gastric cancer, concurrent gastroscopy could be considered. Considering the prevalence of germ cell tumors in female biallelic *MCM8/MCM9* variant carriers, annual ultrasound screening starting at age 10 could be considered, given the early onset of 11–15 years observed in our case series. Further evaluation of cancer risks and the cost-effectiveness of surveillance measures is necessary to develop comprehensive surveillance guidelines.

In contrast to biallelic *MCM8/MCM9* variant carriers, our current data suggest that the phenotype of monoallelic *MCM8*/*MCM9* variant carriers may primarily be limited to hypogonadism, with no clear evidence of an increased cancer risk, which does not seem to justify cancer surveillance for these individuals. Although the prevalence of hypogonadism among monoallelic carriers in our case series (29% for *MCM8*, 22% for *MCM9*) appears higher than the global prevalence (e.g., 3.5% for POI^84^), the potential ascertainment bias in our study, as previously discussed, highlights the need for further research to more fully characterize the phenotype of monoallelic *MCM8/MCM9* variant carriers.

To gain potential causal evidence for a role of *MCM8/MCM9* deficiency in the development of polyps and cancer, future studies exploring the mutational landscape of tumors from *MCM8/MCM9* variant carriers are essential. In the mutational signature analysis from our case series, we observed that clock-like mutational signatures SBS1 and SBS5 dominate in tumors from *MCM8/MCM9* variant carriers with matched germline sequencing data available. However, these clock-like mutational processes, commonly found in most CRCs without specific DNA repair defects and in many other cancer types,^67^^,^^68^^,^^69^^,^^70^^,^^71^ were not more prevalent in tumors from *MCM8/MCM9* variant carriers than in those from our wild-type control. Mutational signatures associated with HR and MMR deficiency, both linked to MCM8 and MCM9 dysfunction,^4^^,^^14^^,^^15^^,^^16^^,^^17^^,^^18^^,^^19^^,^^20^^,^^21^ were observed in only a minority of tumors, predominantly those lacking matched germline sequencing data. In contrast, ID signatures of unknown etiology were present in nearly all tumors from our case series. Further studies are therefore needed to determine whether tumors from *MCM8/MCM9* variant carriers are molecularly similar to sporadic cases, or whether additional, unrecognized mutational signatures may be associated with *MCM8/MCM9* deficiency.

In conclusion, our study offers a detailed clinical and molecular characterization of biallelic *MCM8/MCM9* variant carriers from various sources. Our data suggest that biallelic *MCM9* variants are associated with polyposis, gastric cancer, and early-onset CRC, while both biallelic *MCM8* and *MCM9* variants are linked to hypogonadism and the early development of germ cell tumors. These findings support the inclusion of *MCM8/MCM9* in diagnostic gene panels for specific clinical contexts and indicate that carriers might benefit from cancer surveillance. Further studies are essential to accurately assess cancer risk and determine the causative role of *MCM8/MCM9* deficiency in cancer predisposition.

## Data and code availability

Original/source data for the population-based analyses presented in the paper are available from the following public repositories: https://gnomad.broadinstitute.org/, https://www.ukbiobank.ac.uk/, and https://www.genomicsengland.co.uk/. Original/source data for the bioinformatic analyses of publicly available WGS datasets is accessible via https://www.hartwigmedicalfoundation.nl/ and https://www.cbioportal.org/.

The datasets supporting the analysis of the case series and cancer-specific cohorts in this study have not been deposited in a public repository due to restrictions from our IRB. However, they are available from the corresponding author upon reasonable request and subject to a data transfer agreement.

## Acknowledgments

Please see the supplemental information.

## Author contributions

N.C.H.: conceptualization, methodology, formal analysis, investigation, writing – original draft, and visualization. D.C.: formal analysis and writing – review & editing. M.C.J.J., I.v.d.B., T.F.E., A.G., F.J.H., M.M.v.d.H.-E., A.V.D.K., S.K., R.P.K., I.M.M.L., L.E.E.L.O.L., L.H.J.L., M.S.O., J.S., Y.T.-R., C.M.T., F.T., R.M.d.V., D.W., and M.J.W.: investigation and writing – review & editing. C.P., D.T., H.M., R.H.P.V., A.R., M.G., M.A., L.B., M.T., and L.V.: formal analysis, investigation, and writing – review & editing. T.Y., M.D.G., L.B.A., H.M., and T.v.W.: methodology, formal analysis, investigation, and writing – review & editing. S.C.-B. and Y.G.: conceptualization, methodology, formal analysis, investigation, and writing – review & editing. M.N.: conceptualization, methodology, formal analysis, investigation, writing – review & editing, and supervision.

## Declaration of interests

The authors declare no competing interests.

## Web resources

Castor: https://castoredc.com/
cBioPortal: https://www.cbioportal.org/
gnomAD: https://gnomad.broadinstitute.org/
gnomAD Browser Variant Co-occurrence: https://gnomad.broadinstitute.org/variant-cooccurrence?dataset=gnomad_r2_1
Hartwig Medical Foundation: https://www.hartwigmedicalfoundation.nl/
Online Mendelian Inheritance in Man: http://www.omim.org/
Picard: http://broadinstitute.github.io/picard
VariantValidator: https://www.variantvalidator.org/
UK Biobank: https://www.ukbiobank.ac.uk/

## Supplemental information

Document S1. Figures S1–S7, Tables S1–S3, and supplemental notes

Data S1. DNA variant HGVS nomenclature verification

Document S2. Article plus supplemental information
