# Supplementary material for: Clinical syndromes linked to biallelic germline variants in MCM8 and MCM9
Source: HGG Adv. 2025 Jul 18;6(4):100480. doi: 10.1016/j.xhgg.2025.100480 (PMC12361757; doi:10.1016/j.xhgg.2025.100480)
Supplement: Document S1. Figures S1–S7, Tables S1–S3, and supplemental notes [file mmc1.pdf]

HGGA, Volume *6*

Supplemental information

Clinical syndromes linked to biallelic germline variants in *MCM8* and *MCM9*

Noah C. Helderman, Ting Yang, Claire Palles, Diantha Terlouw, Hailiang Mei, Ruben H.P. Vorderman, Davy Cats, Marcos Díaz-Gay, Marjolijn C.J. Jongmans, Ashwin Ramdien, Irma van de Beek, Thomas F. Eleveld, Andrew Green, Frederik J. Hes, Marry M. van den Heuvel-Eibrink, Annelore Van Der Kelen, Sabine Kliesch, Roland P. Kuiper, Inge M.M. Lakeman, Lisa E.E.L.O. Lashley, Leendert H.J. Looijenga, Manon S. Oud, Johanna Steingröver, Yardena Tenenbaum-Rakover, Carli M. Tops, Frank Tüttelmann, Richarda M. de Voer, Dineke Westra, Margot J. Wyrwoll, Mariano Golubicki, Marina Antelo, Laia Bonjoch, Mariona Terradas, Laura Valle, Ludmil B. Alexandrov, Hans Morreau, Tom van Wezel, Sergi Castellví-Bel, Yael Goldberg, and Maartje Nielsen

Table of Contents

Acknowledgements........ 2

Supplemental case reports 3

Supplemental figures 7

Figure S1 7

Figure S2 11

Figure S3 12

Figure S4 13

Figure S5 15

Figure S6 17

Figure S7 18

Supplemental tables 19

Table S1. 19

Table S2. 21

Table S3. 28

Supplemental references 29

Acknowledgements

The authors sincerely thank all individuals and their families for their participation in this study. This research has been conducted using data from UK Biobank (project code 86977), a major biomedical database (www.ukbiobank.ac.uk). Moreover, this research was made possible through access to data in the National Genomic Research Library (project code 1142), which is managed by Genomics England Limited (a wholly owned company of the Department of Health and Social Care). The National Genomic Research Library holds data provided by patients and collected by the NHS as part of their care and data collected as part of their participation in research. The National Genomic Research Library is funded by the National Institute for Health Research and NHS England. The Wellcome Trust, Cancer Research UK and the Medical Research Council have also funded research infrastructure. Moreover, this work is supported (not financially) by the European Reference Network on Genetic Tumour Risk Syndromes (ERN GENTURIS). ERN GENTURIS is funded by the European Union. Finally, this publication and the underlying study have been made possible partly based on data that Hartwig Medical Foundation and the Center of Personalised Cancer Treatment (CPCT) have made available to the study through the Hartwig Medical Database (reference number HMF-DR-288).

MD-G was supported by a fellowship within the "Generación D" initiative, Red.es, Ministerio para la Transformación Digital y de la Función Pública, for talent attraction (C005/24-ED CV1), being by the European Union NextGenerationEU funds, through PRTR. MG and MA were supported by Foundation Nelia et Amadeo Barletta and the Argentinian National Cancer Institute. LB and SCB were supported by Fondo de Investigación Sanitaria/FEDER (20/00113, 23/00189), Fundació La Marató de TV3 (2019-202008-10), Fundación Científica de la Asociación Española contra el Cáncer (PRYGN211085CAST), "la Caixa" Banking Foundation, CERCA Program (Generalitat de Catalunya), and Agència de Gestió d'Ajuts Universitaris i de Recerca (Generalitat de Catalunya, GRPRE 2017SGR21). CIBEREHD and CIBERONC are funded by the Instituto de Salud Carlos III. The work was carried out (in part) at the Esther Koplowitz Centre, Barcelona. MT and LV's research activity is funded by the Spanish Ministry of Science and Innovation (Agencia Estatal de Investigación), co-funded by FEDER funds (PID2020-112595RB-I00). JS was funded by the German Federal Ministry for Education and Research (BMBF) as part of the Junior Scientist Research Centre 'ReproTrackMS' (grant 01GR2303). FT was supported by grants from the German Research Foundation in the frame of the Clinical Research Unit 'Male Germ Cells' (DFG CRU326, project number 329621271). MJW was supported by the DFG Walter Benjamin Programme (award WY 215/1-1). The funders had no role in the study design, data acquisition and analysis, decision to publish, or preparation of the manuscript.

Supplemental case reports

*MCM8*_01

In a Dutch, non-consanguineous family, two daughters (III.1, age 15; III.4, age 11) presented sequentially with streak ovaries (lacking primordial follicles) and a type II germ cell tumor. Both germ cell tumors were endodermal sinus tumors originating from dysgerminomas, which in turn arose from gonadoblastomas. At age 13, III.4 also developed an enchondroma in the left femur. Germline genetic testing revealed a pathogenic c.1953+1G>C splice donor and a 2027A>T [p.(Tyr676Phe)] VUS in the *MCM8* gene, both present in the daughters. Segregation analysis showed the pathogenic splice variant was inherited from the healthy father (II.1), and the missense variant from the mother (II.2). II.2 was diagnosed with a well-differentiated infiltrating ductal carcinoma (ER+/PR+) and ductal carcinoma in situ (grade I) at age 48, and with three primary invasive carcinoma NSTs (2xER+/PR+; 1xER+/PR-) at age 56. II.1 and II.2 also had two healthy daughters (III.2, III.3) and two abortions, one spontaneous at 11 weeks. III.2 and III.3 are heterozygous carriers of the missense and splice variants, respectively.

*MCM8*_02

The index patient (III.7) is a heterozygous carrier of the c.2209G>A [p.(Ala737Thr)] VUS in the *MCM8* gene and the benign c.1865C>T [p.(Ala622Val)] variant in the *BRCA1* gene. She has two children (IV.7, IV.9) and was diagnosed with ovarian cancer at age 58, which was treated with debulking and adjuvant chemotherapy. At age 59, she developed non-Hodgkin lymphoma, and at age 67, she was diagnosed with moderately differentiated colorectal cancer (MSS). The ovarian cancer metastasized when she was 68, and the non-Hodgkin lymphoma metastasized when she was 83. Her father (II.10), paternal half-brother (III.4), and maternal aunt (II.13) were diagnosed with colorectal cancer at ages 66, 63, and 88, respectively. Her paternal grandmother (I.2) and three paternal aunts (II.1, II.2, II.8) were diagnosed with breast cancer at ages 68-75, 95, 57, and 78, respectively.

*MCM8*_03

The index patient (III.6) presented to the clinical genetics department after the detection of 30 polyps (tubular adenomas with low-grade dysplasia) at age 63. Whole exome sequencing (WES) identified a heterozygous c.482A>G [p.(His161Arg)] variant of uncertain significance in the *MCM8* gene, and three heterozygous variants in the *HROB* gene, including the likely pathogenic c.1267C>T [p.(Gln423*)] variant and two missense variants, c.1363C>G [p.(Leu455Val)] and c.1318A>G [p.(Ser440Gly)]. The latter two variants were in cis on one haplotype and classified as variants of uncertain significance according to the American College of Medical Genetics and Genomics guidelines. Notably, the c.1318A>G [p.(Ser440Gly)] variant may introduce a cryptic splice site leading to protein disruption.

Besides polyps, III.6 had a son (IV.4) via a donor egg due to dysfunctional ovaries lacking oocytes. She experienced nocturnal generalized epileptic seizures between ages 42-46, low bone density at age 53, and COPD (GOLD II) at age 55. Her unrelated Dutch parents (II.10, II.11) included her father, who had acute myelocytic leukemia and died at 84, and her mother, who had a tubulovillous adenoma at 70±1 and died at 83. They had seven children, including five daughters (III.2, III.3, III.6, III.10, III.11) and three sons (III.7, III.8, III.9), with the youngest two being fraternal twins.

Genetic testing of two relatives, a brother (III.8) and a maternal uncle (II.21), revealed that both did not have the *MCM8* variant. The brother carried all three *HROB* variants, developed a squamous cell carcinoma on his ear at 49, was diagnosed with over 150 polyps from age 59, was infertile (azoospermia), and experienced partial seizures before age 29, controlled with carbamazepine. The uncle, negative for *MCM8* and *HROB* variants, developed moderately differentiated rectal adenocarcinoma (Dukes C2; MMR proficient) at 59, treated with abdominoperineal rectum amputation and radiotherapy. He also had multiple (>45) adenomatous polyps and six facial basal cell carcinomas at ages 85 and 88.

Other family members, not tested for *MCM8/HROB* variants, were diagnosed with polyps (II.22, III.2, III.9) and/or cancer (I.4, II.12, II.18, II.19, II.22, III.3, III.9).

***MCM8*_04**

Multiple family members (I.4, III.2, III.6, III.7, III.9, III.15, IV.1) were affected by (early-onset) breast cancer. Germline whole exome sequencing analysis of the index patient (III.15), who was diagnosed with breast cancer at 45 and died from unspecified colon pathology, revealed two heterozygous VUS in the *MCM8* gene: c.692T>A [p.(Ile231Lys)] and c.994A>G [p.(Thr332Ala)]. Additionally, a heterozygous pathogenic variant in the *CHEK2* gene, c.1100del [p.(Thr367Met*15)], was identified. Neither the *MCM8* nor *CHEK2* variants fully segregated with the breast cancer phenotype. The *CHEK2* variant was found in a daughter (IV.1) of the index's sister (III.1; obligate *CHEK2* carrier), both of whom had breast cancer at ages 49 and 41, respectively, but was absent in other breast cancer-affected family members (III.6, III.7, III.9). The *MCM8* c.692T>A [p.(Ile231Lys)] variant was present in a maternal uncle (II.5) and four sisters (III.9, III.11, III.13, III.17) of the index, with only one (III.9) affected by breast cancer at age 67. The *MCM8* c.1100del [p.(Thr367Met*15)] variant was identified in one sister of the index (III.11), who, like the index, is a compound heterozygous carrier of both *MCM8* variants but had no reported cancer or other pathologies. Both *MCM8* variants were absent in III.1, III.4, III.6, III.7, IV.2, IV.7, IV.9, and IV.10.

***MCM8*_05**

A 40-year-old male, previously reported by Golubicki et al.[1], was diagnosed with stage IIIB (T4N1M0) left-sided colon cancer. The tumor was a well-differentiated, mucinous adenocarcinoma. The patient reported no family history of cancer but did mention fertility problems, including his spouse experiencing an advanced pregnancy miscarriage. Genetic testing revealed the pathogenic c.351_354del [p.(Lys118Glu*5)] and benign c.414A>G [p.(Ile138Met)] variants in the *MCM8* gene. Previous tumor analysis in this patient showed an MSI-positive (microsatellite instability) phenotype with the loss of MLH1/PMS2 protein expression, while *BRAF* V600E was wildtype, and there was no somatic *MLH1* promotor hypermethylation. Somatic WES indicated a high tumor mutational burden of 77 single nucleotide variants per megabase, with a significant contribution from the MMR deficiency-associated mutational signature SBS15.

***MCM9*_01**

A patient with MMR proficient CRC was found to carry a heterozygous pathogenic c.1987dup [p.(Ser663Phe*36)] variant in the *MCM9* gene. This patient also had a family history of CRC.

***MCM9*_02**

In a Dutch family with multiple cancer and polyposis cases, the index patient (III.1) was found to carry a c.1915C>G [p.(Leu639Val)] VUS in the *MCM9* gene. This patient was diagnosed with MMR proficient adenocarcinoma of the sigmoid colon at 25 years old and metastasized signet ring cell carcinoma at 36, resulting in his death. The patient's father (II.3) and paternal grandfather (I.1) had melanoma and lung cancer, respectively, while his mother (II.4) and all maternal uncles (II.5, II.7) and aunts (II.8, II.10, II.12, II.14) had polyps. One maternal uncle (II.5) also had a diffuse growing adenocarcinoma of the distal esophagus and stomach at 56, and the maternal grandfather (I.4) had primary colorectal carcinomas (MMR proficient) at 47, 60, and 69.

***MCM9*_03**

In a consanguineous Ashkenazi family, previously described by Goldberg et al.[2, 3], two female siblings (III.3, III.4) were initially evaluated for hypergonadotropic hypogonadism and POI. Both were diagnosed with multiple polyps (>20) and CRC at young ages (34 and 37 years, respectively) but responded well to FOLFOX chemotherapy, with no current evidence of disease. Germline genetic analysis revealed a homozygous c.672_673delinsC [p.(Glu225*)] variant in the *MCM9* gene, considered likely pathogenic. The siblings' parents (I.3, II.1), their two other female siblings (III.1, III.2), and their maternal grandmother/paternal aunt (I.2) were heterozygous carriers of this variant. The mother (II.1) was diagnosed with over ten polyps between the ages of 53-65, and the father (II.2) was diagnosed with CRC and a polyp at age 83. The other siblings (III.1, III.2) did not have CRC, though III.2 had two polyps detected at age 39.

***MCM9*_04**

In a consanguineous family of Middle Eastern Arabic origin, previously described by Goldberg et al.[3], multiple carriers of the pathogenic c.1483G>T [p.(Glu495*)] variant in the *MCM9* gene were identified. The related parents (II.2, II.3) were both heterozygous carriers of this variant and each had three polyps diagnosed between the ages of 66-68 years. They had three daughters (III.1, III.2, III.4) and two sons (III.3, III.6). Two daughters (III.1, III.2), who were homozygous carriers of the c.1483G>T [p.(E495*)] variant, were diagnosed with POI around age 15. One of these daughters (III.1) was also diagnosed with CRC at age 31, while the other (III.2) was diagnosed with clear cell carcinoma of the cervix at age 37. One son (III.3), a heterozygous carrier of the variant, was diagnosed with severe oligozoospermia and microsatellite stable (MSS) CRC at age 35. The third daughter (III.4), also a heterozygous carrier, had four children.

***MCM9*_05**

This family was previously described by Potorac et al.[4] Two sisters (IV.1, age 24; IV.6, age 17) from consanguineous Syrian parents (II.13, II.14) were diagnosed with POI, characterized by absent ovaries and no or hypotrophic uteri. Genetic analysis revealed a homozygous pathogenic c.394C>T [p.(Arg132*)] variant in the *MCM9* gene in both sisters. Another sister (IV.2) and a brother (IV.3), who later presented with POI and non-obstructive azoospermia, respectively, were also homozygous carriers of this variant. A second brother (IV.7), a heterozygous carrier, had normal puberty. Additionally, III.13, III.14, IV.1, IV.3, and IV.6 all suffered from severe osteoporosis. Genetic panel analysis for osteoporosis in IV.3 revealed a heterozygous c.106G>A [p.(Gly36Arg)] variant in the *COL1A2* gene, although this variant did not fully segregate with the bone pathology (III.13 was wildtype, while III.14 and IV.6 were heterozygous carriers).

***MCM9*_06**

The index patient (III.1) was diagnosed with POI at 19 years old, along with her twin sisters (III.4, III.5), who never experienced puberty and exhibited streak ovaries and a small uterus. Their father (II.3) is Irish, and their unrelated mother (II.4) is Hungarian, with no reported family history of cancer and normal colonoscopy results for both parents. The index patient's brothers (III.2, III.3) had normal puberty and health, although they have not had children. During her first pregnancy at age 39 (using a donor egg), the index patient (III.1) developed severe diarrhea, leading to the discovery of a likely pathogenic c.1720C>T [p.(Arg574*)] variant and a c.1529-3C>A VUS in the *MCM9* gene through germline genetic analysis. Segregation analysis revealed that the father (II.3) carries the heterozygous c.1720C>T [p.(Arg574*)] variant, while the mother (II.4) carries the heterozygous c.1529-3C>A variant. Since identifying the biallelic *MCM9* variants, the index patient (III.1) has undergone colonoscopies every six months, with 1-2 polyps removed per procedure. The siblings of the index patient did not undergo genetic testing or colonoscopies.

***MCM9*_07**

At 16 years old, the index patient (IV.2) from a Belgian family presented with primary amenorrhea and delayed puberty. Genetic testing revealed compound heterozygous variants in the *MCM9* gene: c.820C>T [p.(Gln274*)] and c.2237_2238dup [p.(Phe47I78)], both classified as likely pathogenic, along with the likely pathogenic c.1222G>T [p.(Asp408Tyr)] variant in the *FSHR* gene. Segregation analysis showed that the father (III.6), who also experienced delayed puberty, carried the heterozygous c.2237_2238dup [p.(Phe47I78)] variant, while the mother (III.7) carried the heterozygous c.820C>T [p.(Gln274*)] variant. The index patient's parents were unrelated.

***MCM9*_08**

The index patient (III.8), a mother of two daughters, carried the compound heterozygous variants c.3425A>G [p.(Lys1142Arg)] and c.1640T>C [p.(Leu547Pro)] in the *MCM9* gene, both classified as VUS per ACMG/AMP guidelines.[5, 6] At 42 years old, III.8 was diagnosed with metastatic poorly differentiated colorectal cancer (MMR proficient), which initially metastasized to the lymph nodes. Despite treatment including rectum resection, chemotherapy (5-fluorouracil/leucovorin), and

radiotherapy, the cancer spread to the liver, leading to her death at 43 years old. The index's brother died from metastatic pancreatic cancer at age 48, while a maternal aunt (II.8) succumbed to leukemia at age 73, and a paternal aunt (II.2) had myoepithelial adenocarcinoma of the left parotid gland at age 67, two primary colorectal cancers (at age 71 and 77), and lobular carcinoma of the right breast at age 76.

***MCM9*_09**

After experiencing a miscarriage (IV.7), the index patient (III.9) was diagnosed with POI before the age of 40. She and her partner (III.8), who is in good health, adopted two daughters. At age 46, III.9 was diagnosed with moderately differentiated colorectal cancer (microsatellite instability, MSH2-/MSH6-), and multiple colon polyps were subsequently found. By age 61, she developed poorly differentiated stomach adenocarcinoma (MMR proficient, microsatellite stable), treated with fluorouracil, oxaliplatin, and docetaxel (FLOT). WES revealed heterozygous variants c.1642C>T [p.(Arg548Trp)] and c.152A>T [p.(Asn51Ile)] in the *MCM9* gene, both classified as VUS. These variants were also detected in III.7, her brother, who developed well-differentiated colorectal cancer (MMR proficient, microsatellite stable) at 55, alongside over 70 colon polyps and melanoma at 60. III.7's son (IV.4) was born with an open abdomen, dextrocardia, and pulmonary agenesis. Another brother (III.2) of III.9, survived by his two children (IV.1, IV.2), died from small cell lung cancer at 59.

***MCM9*_10 (M2013)**

The index patient (III.1) from a consanguineous Syrian family consulted for infertility and was diagnosed with azoospermia at 28 years of age. Because of his small testicular volumes (7/8 mL, left/right, reference >12mL per testis) and significantly increased serum FSH (27.9 U/L, reference range 1-7 U/L), he most likely had spermatogenic failure. Indeed, an externally performed testicular punch biopsy showed spermatocytes as most advanced germ cells indicating an arrest at meiosis confirming that the infertility is due to non-obstructive azoospermia (NOA). Exome sequencing within the Male Reproductive Genomics (MERGE) study revealed that he carried the homozygous pathogenic loss of function variant c.394C>T [p.(Arg132*)] in the *MCM9* gene. The case/variants have been reported before in Wyrwoll et al.[7] No other family members underwent testing for this variant because they did not consent to be included in the study. His sister (III.7) was diagnosed with colorectal cancer at 35 years old, and another sister (III.10) was diagnosed with POI. One of his brothers (III.9) had congenital intellectual disability (microcephaly), and two cousins (III.12, III.13) passed away due to consequences of mental retardation.

***MCM8*_*MCM9*_01**

The index patient (III.2) carried a heterozygous c.832C>T, [p.(Arg278Cys)] VUS in the *MCM8* gene and a heterozygous c.3425A>G, [p.(Lys1142Arg)] VUS in the *MCM9* gene. She was diagnosed with a moderately differentiated colorectal cancer (RER-) at 52 years old. Despite undergoing left-sided hemicolectomy and treatment with 5-fluorouracil/levamisole, the cancer metastasized to the distal ileum, pelvis minor, and an ovary by age 54, leading to her death at 56. The index patient, who had two sons (IV.1, IV.2), also suffered from ovarian cysts and was diagnosed with an adenomatous polyp and a hyperplastic polyp in the colon at 53 years old. Her brother (III.2), father (II.3), and paternal uncle (II.1) were all diagnosed with colorectal cancer. The father (II.3) also developed stomach cancer at 79 years old, while the paternal grandfather (I.1) of the index had a history of either colorectal or stomach cancer (unclear based on medical records).

Supplemental figures

Figure S1

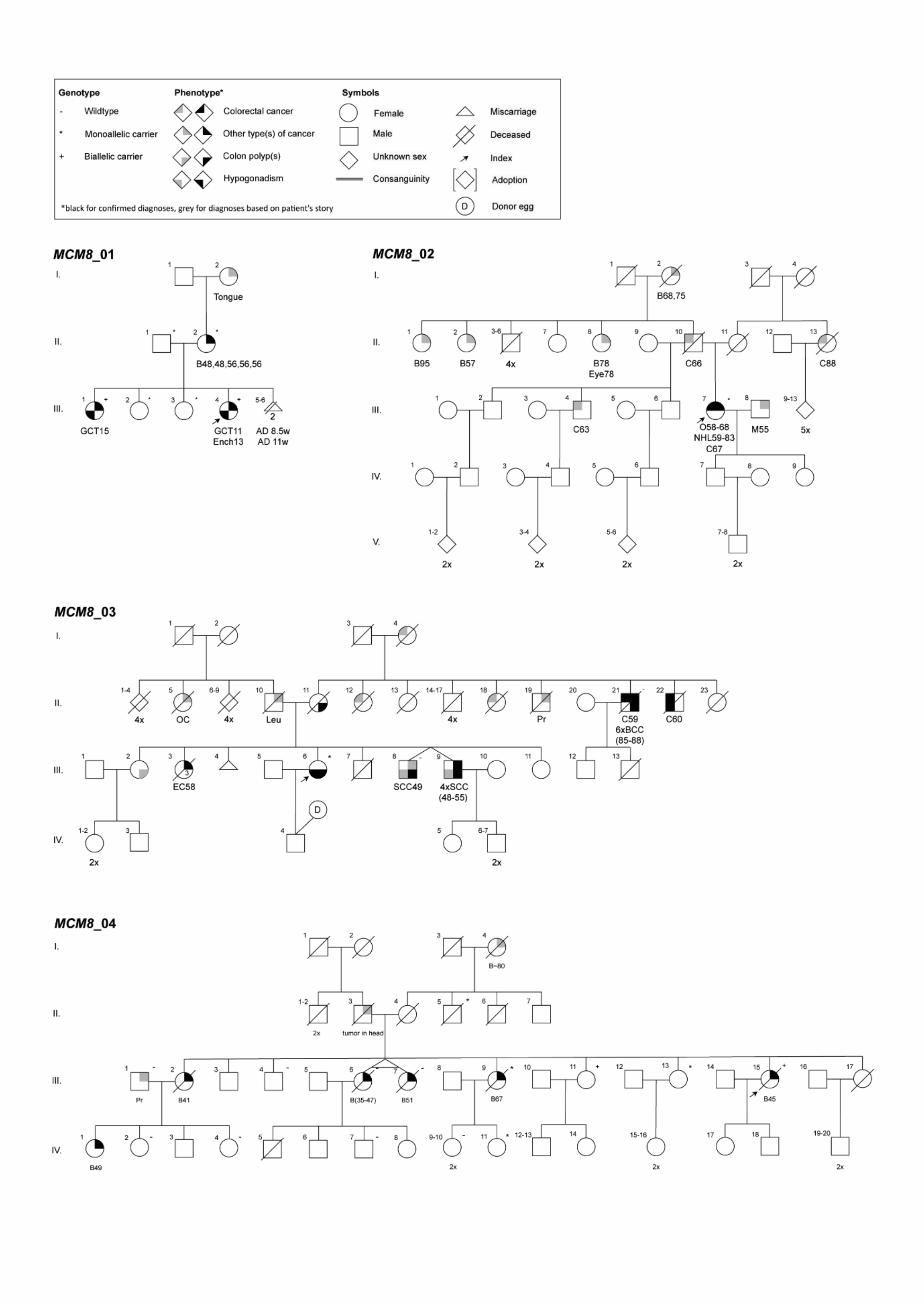

Figure S1 [continued]

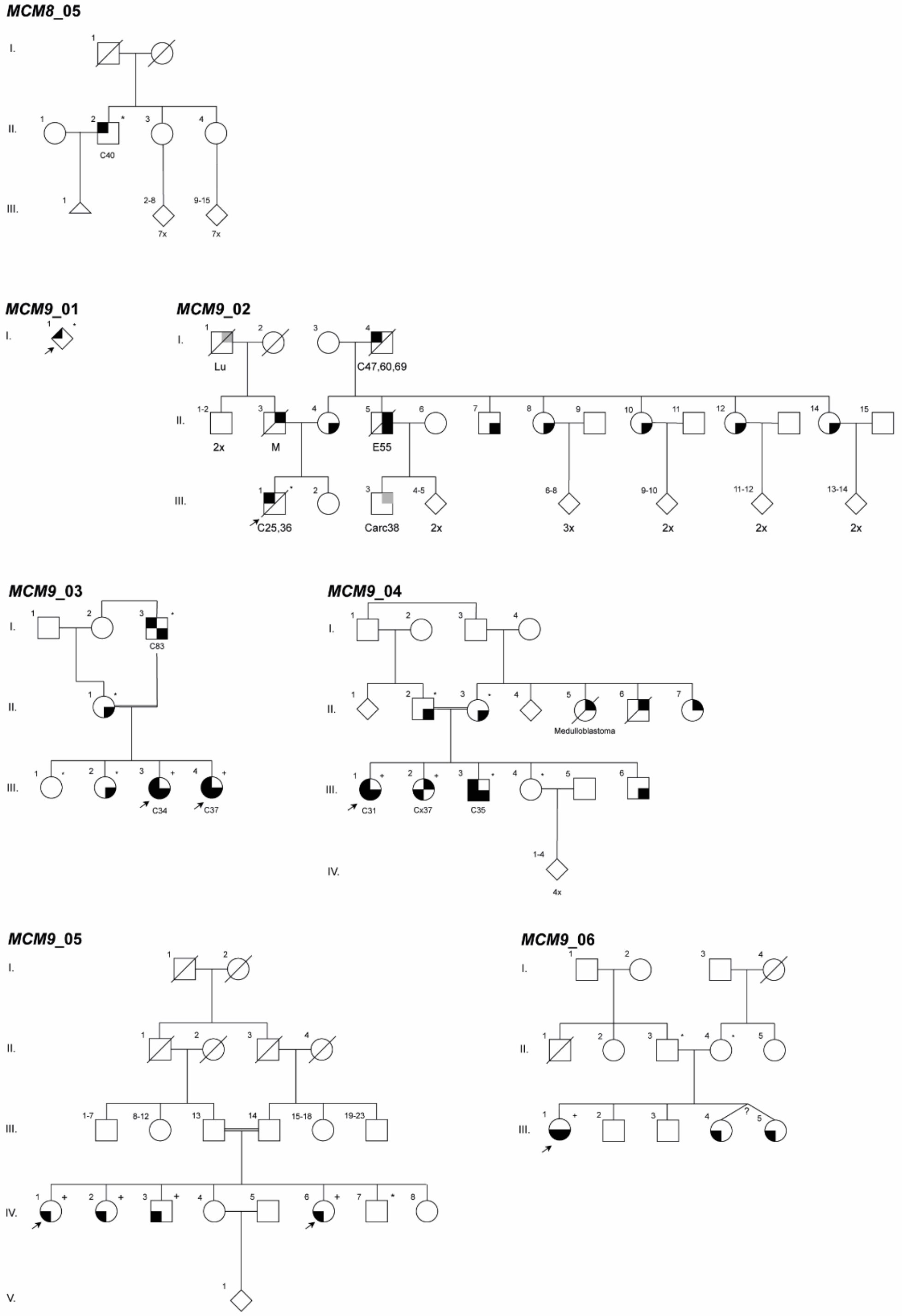

Figure S1 [continued]

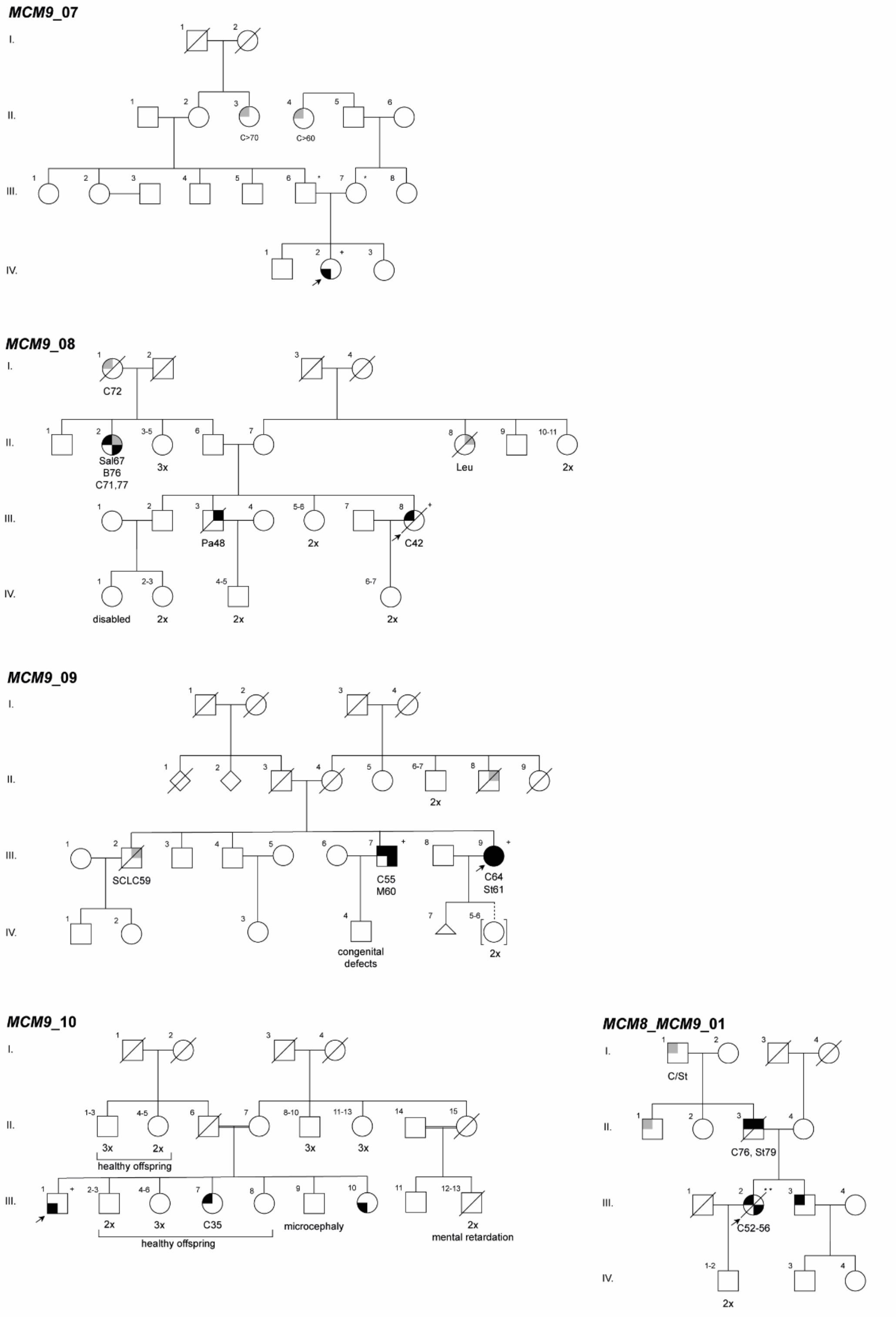

Figure S1. Pedigrees of all newly identified *MCM8/MCM9* variant carriers and previously documented carriers for whom we obtained updated clinical information. Only pedigrees of families

with variants meeting the pathogenicity-based filtering criteria are shown. *AD, amenorrhoea duration; B, breast cancer; BCC, basal cell carcinoma; C, colorectal cancer; Cx, cervical cancer; E, esophagus cancer; EC, endometrial cancer; Ench, enchondroma; GCT, germ cell tumor; Leu, leukemia; Lu, lung cancer; M, melanoma; NHL, non-Hodgkin lymphoma; O, ovarian cancer; Pa, pancreatic cancer; Pr, prostate cancer; Sal, salivary gland cancer; SC C, squamous cell carcinoma; SCL, small-cell lung cancer; St, stomach cancer.*

Figure S2

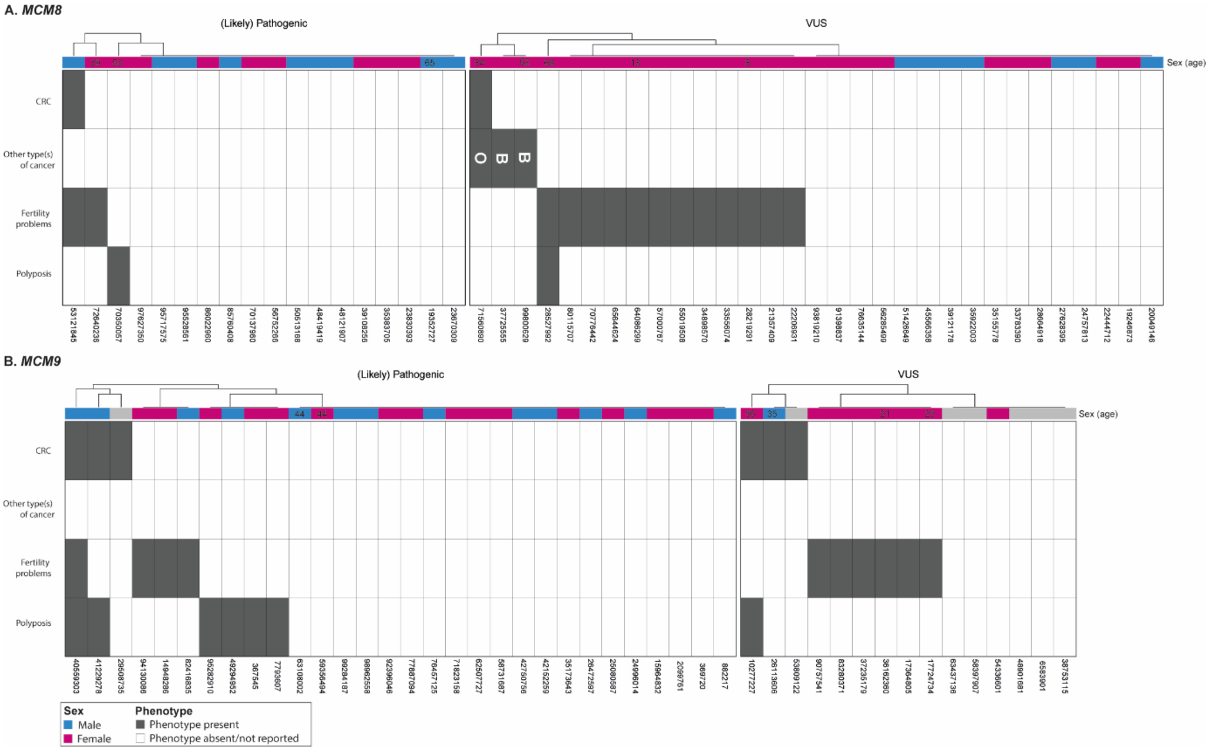

Figure S2. Phenotype of monoallelic *MCM8/MCM9* variant carriers. The phenotype is presented for all (**A**) monoallelic *MCM8* and (**B**) monoallelic *MCM9* variant carriers from our case series. Each column represents an individual, while each row corresponds to one of the four primary observed phenotypes: CRC, other type(s) of cancer, hypogonadism, and polyposis. Person IDs are provided below each column, whereas their corresponding ages, which represent the most recent reported age of each individual, are shown above every column (when available). *B, breast cancer; CRC, colorectal cancer; O, ovarian cancer; VUS, variant of uncertain significance.*

Figure S3

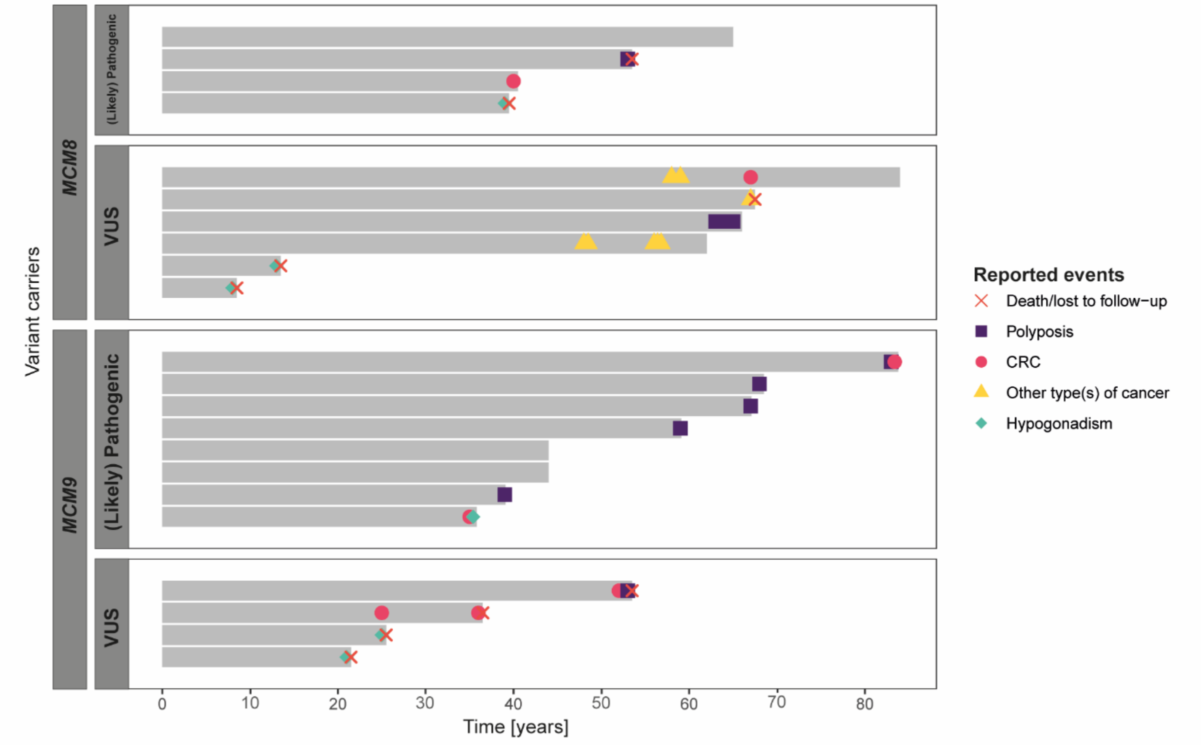

Figure S3. Disease onset in monoallelic *MCM8/MCM9* variant carriers. The onset of the four primary observed phenotypes (CRC, other type(s) of cancer, hypogonadism, and polyposis) is displayed for each monoallelic *MCM8/MCM9* variant carrier with available age details in our case series. Those without age details were excluded from the analysis. Individuals are ordered by ACMG/AMP classification (pathogenic or likely pathogenic, VUS)[55, 56] and current age or age at the time of death/lost to follow-up. *ACMG, American College of Medical Genetics and Genomics; AMP, Association for Molecular Pathology; CRC, colorectal cancer; VUS, variant of uncertain significance.*

Figure S4

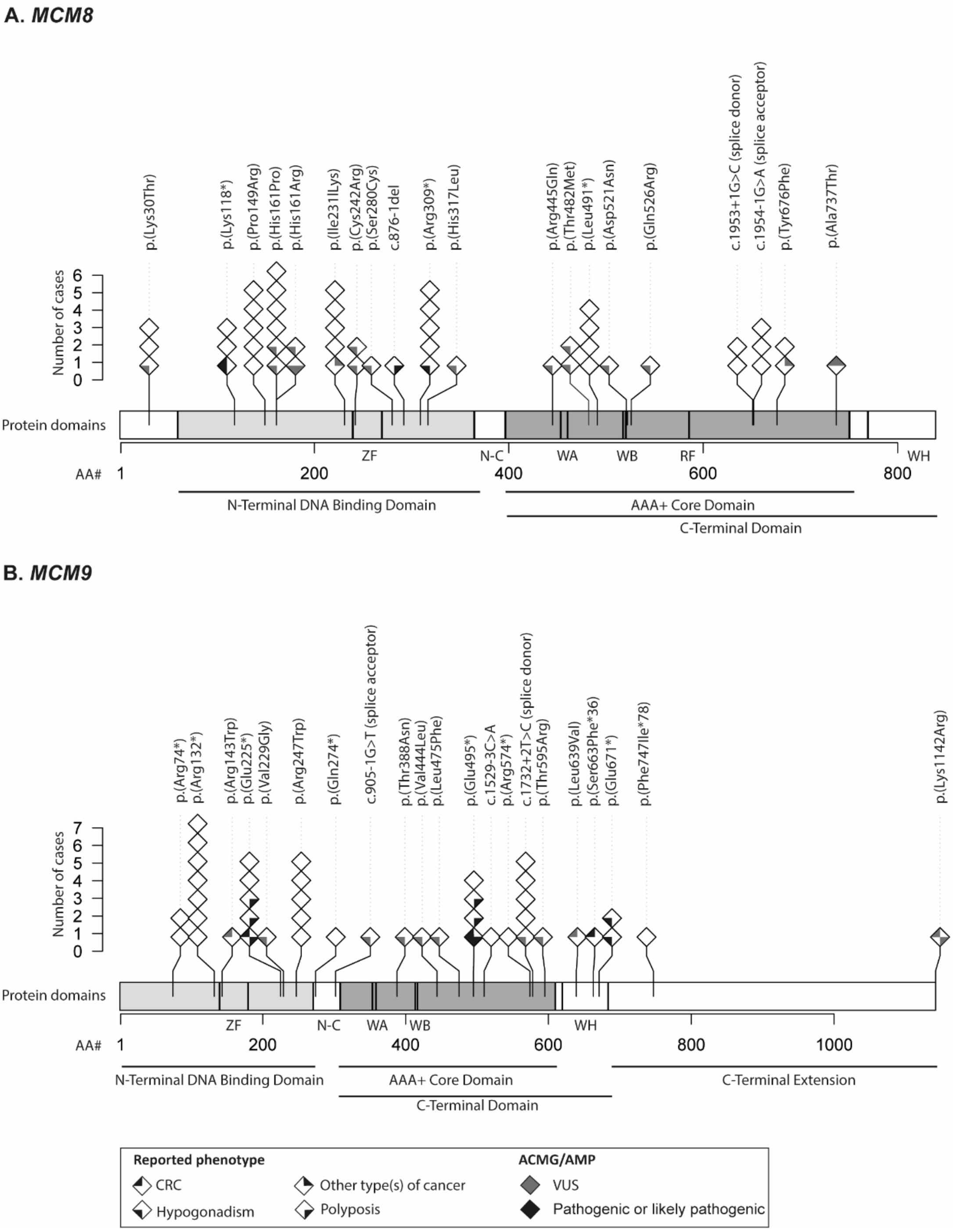

Figure S4. Monoallelic *MCM8/MCM9* variants mapped onto the respective protein domains. (**A**) A*MCM8* and (**B**) *MCM9* variants from all monoallelic variant carriers in our case series are mapped onto the domains of the MCM8 and MCM9 proteins, respectively. The fill and color of the diamond symbols correspond to the phenotype of the individual (CRC, other type(s) of cancer, hypogonadism, polyposis) and the ACMG/AMP classification of the variant (pathogenic or likely pathogenic, VUS)[55, 56], respectively. *ACMG, American College of Medical Genetics and Genomics; AMP, Association for*

Molecular Pathology; CRC, colorectal cancer; RF, arginine finger; VUS, variant of uncertain significance; WA, Walker A; WB, Walker B; WH, winged-helix; ZF, zinc-finger.

Figure S5

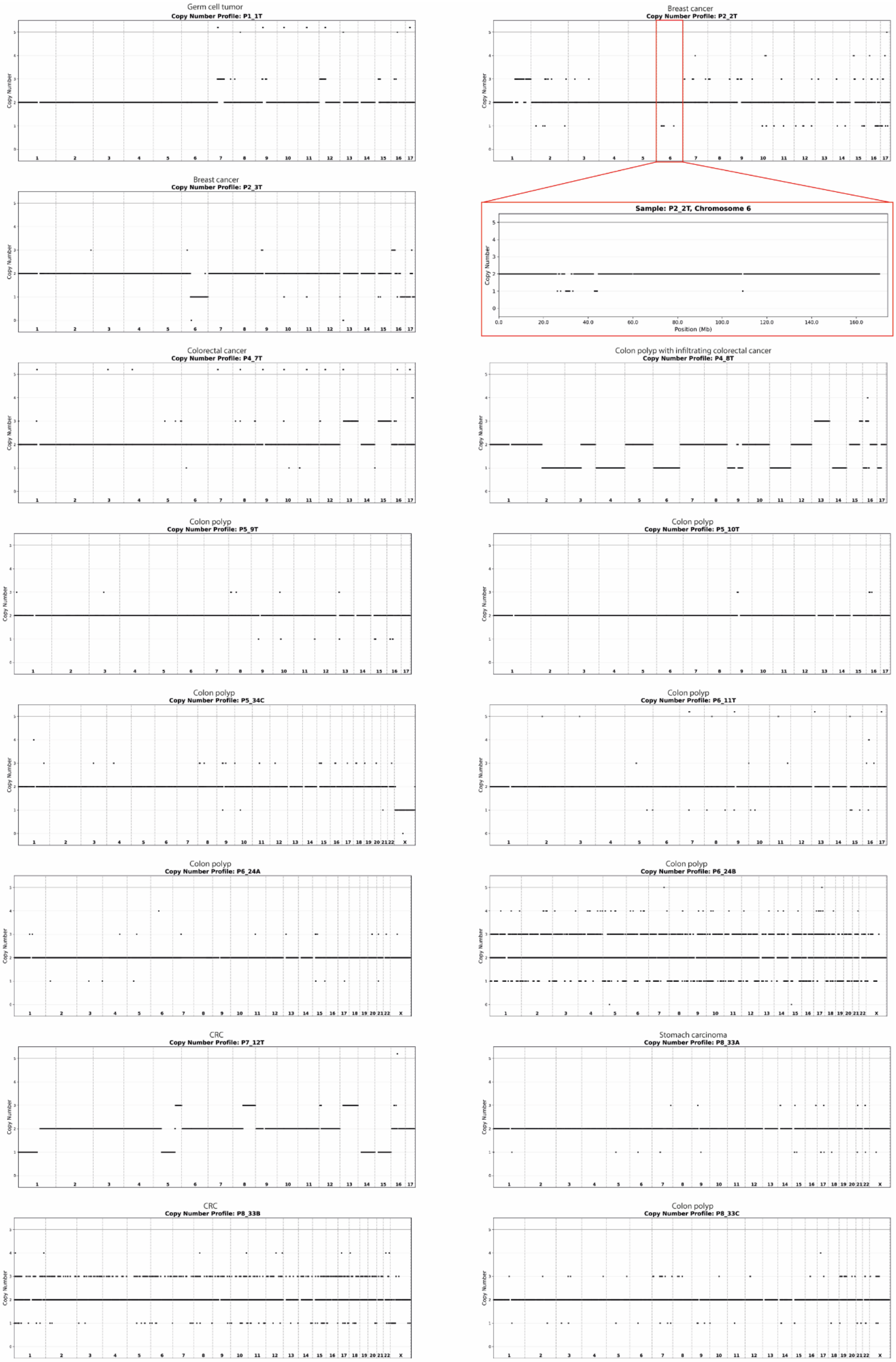
Germ cell tumor
Copy Number Profile: P1_1T
Breast cancer
Copy Number Profile: P2_2T
Breast cancer
Copy Number Profile: P2_3T
Sample: P2_2T, Chromosome 6
Position (Mb)
Colorectal cancer
Copy Number Profile: P4_7T
Colon polyp with infiltrating colorectal cancer
Copy Number Profile: P4_8T
Colon polyp
Copy Number Profile: P5_9T
Colon polyp
Copy Number Profile: P5_10T
Colon polyp
Copy Number Profile: P5_34C
Colon polyp
Copy Number Profile: P6_11T
Colon polyp
Copy Number Profile: P6_24A
Colon polyp
Copy Number Profile: P6_24B
CRC
Copy Number Profile: P7_12T
Stomach carcinoma
Copy Number Profile: P8_33A
CRC
Copy Number Profile: P8_33B
Colon polyp
Copy Number Profile: P8_33C

Figure S5. Copy number analysis of tumors from *MCM8/MCM9* variant carriers from our case series. Total copy number analysis was performed using CNVkit (v0.9.8) on WGS and WES data, processed separately. To improve visual clarity, segments smaller than 100 kb were excluded. Due to figure size constraints, some segments may appear to display two copy number values; however, this is neither a technical error nor artifact, as verified by the individual chromosome plots. A zoom-in of chromosome 6 from P2_2T (red box) illustrates that segments do not overlap. For enhanced readability, segments with copy number values above 5 are displayed above the grey reference bar. *WES, whole-exome sequencing; WGS, whole-genome sequencing.*

Figure S6

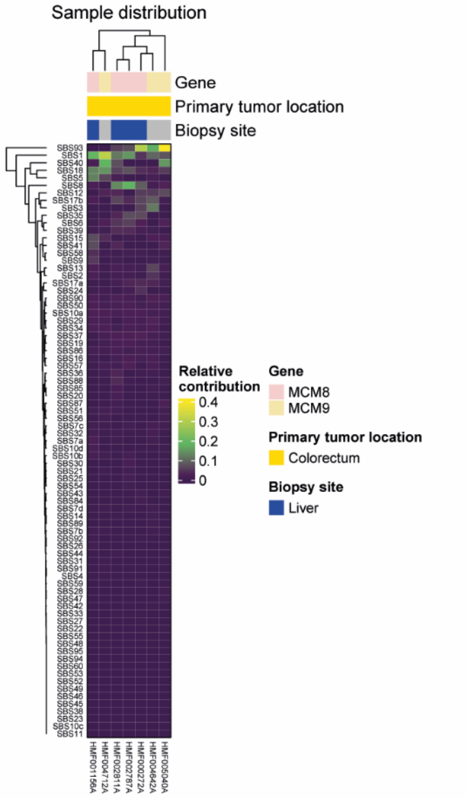

Figure S6. Mutational signature analysis of metastasized CRCs from monoallelic *MCM8/MCM9* variant carriers in the HMF cancer-specific cohort. Mutational signature analysis was conducted on metastasized CRCs from monoallelic *MCM8/MCM9* variant carriers in the HMF cancer-specific cohort. The heatmap displays unsupervised hierarchical clustering of the SBS mutational signature profiles. This analysis included tumors from four monoallelic *MCM8* and three monoallelic *MCM9* variant carriers without a second hit in the *MCM8/MCM9* genes. All germline *MCM8/MCM9* variants were classified as VUS per the ACMG/AMP classification for variant interpretation.[55, 56] The rows represent SBS mutational signatures, while the columns represent individual samples. The identification of SBS mutational signatures was achieved by fitting the counts of SNVs per 96 tri-nucleotide context to the COSMIC signatures[76], employing the MutationalPatterns tool.[77] *ACMG, American College of Medical Genetics and Genomics; AMP, Association for Molecular Pathology; HMF, Hartwig Medical Foundation; SBS, single base substitution; SNV, single nucleotide variant; VUS, variant of uncertain significance.*

Figure S7

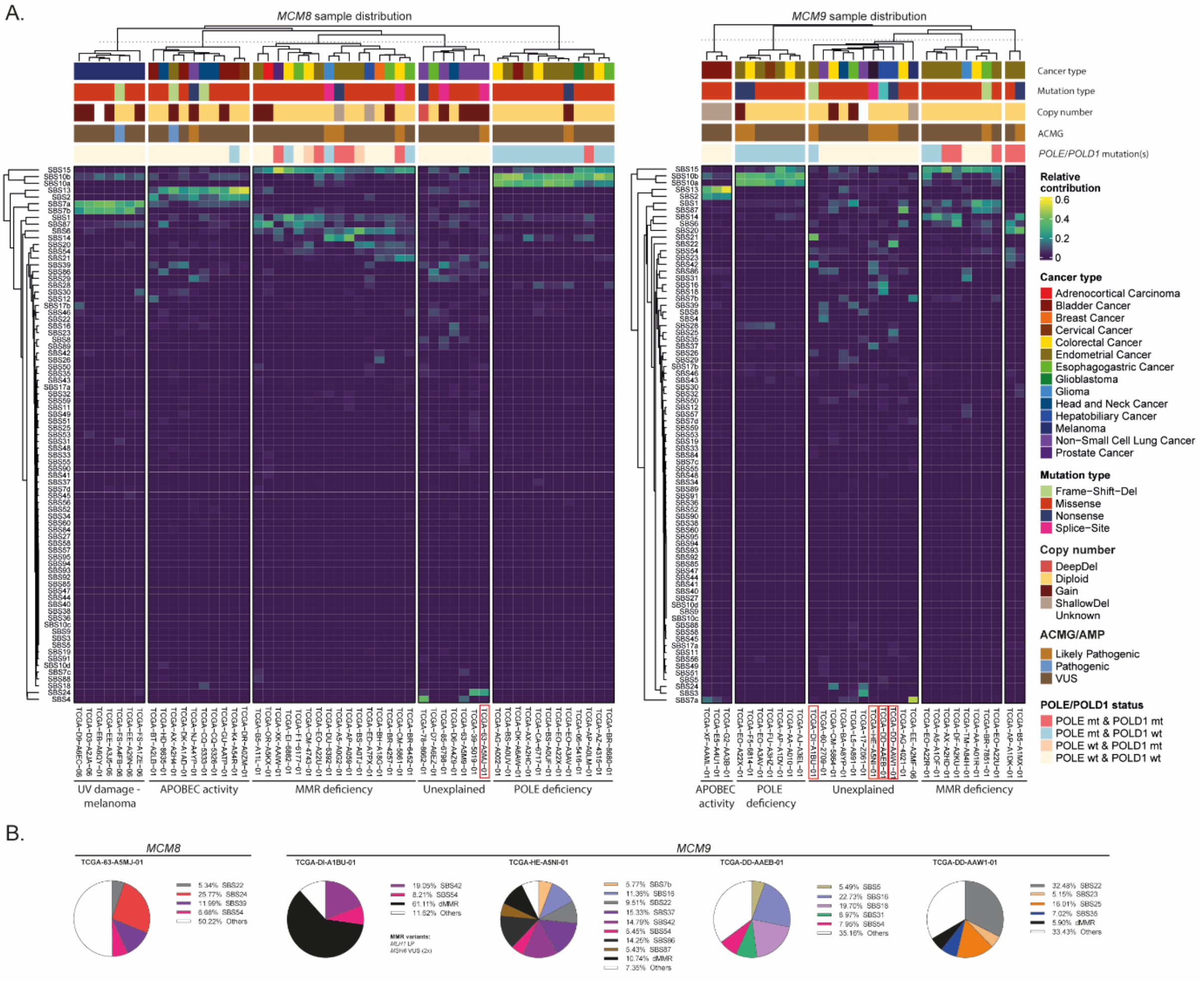

Figure S7. Mutational signature analysis on TCGA Pan-Cancer Atlas samples harboring somatic *MCM8/MCM9* mutation(s). (**A**) Heatmaps showing unsupervised hierarchic clustering of the SBS mutational signature profiles of TCGA Pan-Cancer Atlas tumors with somatic *MCM8/MCM9* mutation(s). Rows represent SBS mutational signatures, while columns represent individual samples. In clusters represented by SBS7a/b (UV damage), SBS2 and SBS13 (APOBEC activity), SBS6, SBS14, SBS15, SBS20, and SBS21 (MMR deficiency), or SBS10a/b (POLE deficiency), the somatic *MCM8/MCM9* variants were likely secondary to other mutational processes.[71, 72, 76, 85, 86] Tumors with likely pathogenic *MCM8* (n=1) or *MCM9* (n=4) variants in the unexplained clusters were marked by red squares. (**B**) In tumors with likely pathogenic *MCM8* (n=1) or *MCM9* (n=4) variants from the unexplained clusters, shared SBS mutational signatures included SBS22 (aristolochic acid exposure), SBS42 (haloalkane exposure), and SBS54 (sequencing artefact). Notably, SBS mutational signatures associated with MMR deficiency (SBS6, SBS14, SBS15, SBS20, SBS21) were also present in three of these five tumors. However, these could be explained by a likely pathogenic *MLH1* variant and two variants of unknown significance in the *MSH6* gene in one (TCGA-DI-A1BU-01) of these tumors. The TCGA Pan-Cancer Atlas was assessed via cBioPortal for Cancer Genomics (https://www.cbioportal.org/) in February-April, 2023. *ACMG, American College of Medical Genetics and Genomics; AMP, Association for Molecular Pathology; LP, likely pathogenic; mt, mutation; SBS, single base substitution; TCGA, The Cancer Genome Atlas; VUS, variant of uncertain significance; wt, wildtype.*

Supplemental tables

Table S1. ICD10 and ICD-O codes and corresponding phenotypes used to identify cohorts for variant enrichment analysis

ICD10 codes	Phenotype	100K Genomes Project		200K UK Biobank	
		n[a]	Median age (range)[b]	n[a]	Median age (range)[b]
K635	Colonic polyps	3051	70 (5-100)	9262	73 (45-84)
K621	Rectal polyps	1404	69 (5-98)	5164	72 (51-84)
D120,D121,D123,D124,D125,D126,D127,D128,D129	Colorectal adenomas	2877	72 (10-99)	10297	73 (45-84)
C180,C181,C182,C183,C184,C185,C186,C187,C188,C189,C19,C20	Colorectal cancer	3473	73 (17-102	3239	74 (42-84)
C509	Breast cancer	4438	66 (22-102)	6783	
C160, C161, C162, C163, C164, C165, C166, C168, C169	Gastric cancer	281	70 (18-97)	481	
C430, C431, C432, C433, C434, C435, C436, C437, C438, C439	Melanoma	714	70 (13-102)	1626	
C541, C542, C543, C548, C549, C55	Endometrial cancer	1107	71 (21-99)	938	
C56, C561, C562,C563, C569	Ovarian cancer	844		696	
C530, C531, C538, C539	Cervical cancer	135		145	
N979	Female infertility	434		495	57 (46-72)
E283	Primary Ovarian Insufficiency	71		24	
N46	Male infertility	20		33	
G400	Localization-related (focal) (partial) idiopathic epilepsy and epileptic syndromes with seizures of localized onset	218	27 (0-89)	25	
G401	Localization-related (focal) (partial) symptomatic epilepsy and epileptic syndromes with simple partial seizures	1413		124	
G402	Localization-related (focal) (partial) symptomatic epilepsy and epileptic syndromes with complex partial seizures	996		193	
G403	Generalized idiopathic epilepsy and epileptic syndromes	2251		313	
G404	Other generalized epilepsy and epileptic syndromes	724		8	
G405	Special epileptic syndromes	81		31	
E343	Short Stature	994		9	

E300	Delayed Puberty	129		0	
E039	Hypothyroidism	3323	63 (0-102)	10997	73 (44-84)
Q510	Absent/Infantile Uteri	13		0	
**ICD-O**	**Phenotype**	**100K Genomes Project**		**200K UK Biobank**	
		**n**[a]	**Median age (range)**[b]	**n**[a]	**Median age (range)**[b]
9061/3<br>9070/3<br>9071/3<br>9100/3<br>9080/0<br>9080/1<br>9085/3<br>9084/3<br>9086/3	Seminoma<br>Embryonal carcinoma<br>Yolk sac tumor<br>Choriocarcinoma<br>Mature teratoma<br>Immature teratoma of the yhymus<br>Mixed germ cell tumor<br>Teratoma with somatic type malignancies<br>Germ cell tumor with associated hematological malignancy	152		216	

[a] n = number of participants with each phenotype. Some participants had multiple ICD10 codes that were included in our search.

[b] The ages of the cases are provided for analyses where variant enrichment could be performed (i.e., at least one homozygous or compound heterozygous case and control was available).

ICD10, International Classification of Diseases 10th Revision; ICD-O, International Classification of Diseases for Oncology

Table S2. Overview of *MCM8/MCM9* variant carriers meeting pathogenicity-based filtering criteria, including sources

Current study ID	Current family ID (only for new cases from outpatient clinic or carriers from literature with updated data)	Source	Digital Object Identifier (DOI) of source (if applicable)	Study ID used in source (if applicable)	Germline *MCM8* variant(s)	Germline *MCM9* variant(s)
**Biallelic *MCM8* (VUS)**						
01143258		Carriers from literature - data from papers only	10.1016/j.fertnstert.2017.07.015	V-11	c.482A>C, p.(His161Pro)	
02328912	*MCM8*_04	Carriers from literature - updated data	10.1172/jci.insight.140698	SXS48	c.692T>A, p.(Ile231Lys); c.994A>G, p.(Thr332Ala)	
07868858		Carriers from literature - data from papers only	10.1016/j.ajhg.2022.01.011	P0281	c.482A>C, p.(His161Pro)	
15517012	*MCM8*_04	Outpatient clinic			c.692T>A, p.(Ile231Lys); c.994A>G, p.(Thr332Ala)	
28590785		Carriers from literature - data from papers only	10.1016/j.fertnstert.2017.07.015	V-5	c.482A>C, p.(His161Pro)	
39848302		Carriers from literature - data from papers only	10.1016/j.fertnstert.2017.07.015	V-9	c.482A>C, p.(His161Pro)	
48582770		Carriers from literature - data from papers only	10.1172/JCI78473	IV-9	c.446C>G, p.(Pro149Arg)	
55456091		Carriers from literature - data from papers only	10.1172/JCI78473	IV-6	c.446C>G, p.(Pro149Arg)	
71809414		Carriers from literature - data from papers only	10.1016/j.fertnstert.2017.07.015	V-10	c.482A>C, p.(His161Pro)	
92426276		Carriers from literature - data from papers only	10.1172/JCI78473	IV-1	c.446C>G, p.(Pro149Arg)	
96184605		Carriers from literature - data from papers only	10.1016/j.ajhg.2022.01.011	P0370	c.482A>C, p.(His161Pro)	
**Biallelic *MCM8* (pathogenic or likely pathogenic)**						
01130852		Carriers from literature - data from papers only	10.1210/jc.2019-00248	BAB7675	c.925C>T, p.(Arg309*)	
08232281		Carriers from literature - data from papers only	10.1136/jmedgenet-2014-102921	IV-6	c.1470_1471insTA, p.(Leu491fs)	
13147827		Carriers from literature - data from papers only	10.1002/mgg3.1165	IV-1	c.351_354del, p.(Lys118fs)	
23867510		Carriers from literature - data from papers only	10.1136/jmedgenet-2014-102921	V-1	c.1954-1G>A, splice acceptor	
25350218		Carriers from literature - data from papers only	10.1002/mgg3.1165	IV-3	c.351_354del, p.(Lys118fs)	
26298807	*MCM8*_01	Outpatient clinic			c.2027A>T, p.(Tyr676Phe); c.1953+1G>C, splice donor	

38595546		Carriers from literature - data from papers only	10.1136/jmedgenet-2014-102921	V-2	c.1954-1G>A, splice acceptor	
43675980		Carriers from literature - data from papers only	10.1136/jmedgenet-2014-102921	IV-3	c.1470_1471insTA, p.(Leu491fs)	
45685855		Carriers from literature - data from papers only	10.1210/clinem/dgaa155	IV-2	c.925C>T, p.(Arg309*)	
46951971		Carriers from literature - data from papers only	10.1210/jc.2019-00248	BAP767 5	c.925C>T, p.(Arg309*)	
54907964		Carriers from literature - data from papers only	10.1136/jmedgenet-2014-102921	IV-2	c.1470_1471insTA, p.(Leu491fs)	
57322856		Carriers from literature - data from papers only	10.1136/jmedgenet-2014-102921	IV-7	c.1470_1471insTA, p.(Leu491fs)	
74081581		Carriers from literature - data from papers only	10.1136/jmedgenet-2014-102921	IV-4	c.1470_1471insTA, p.(Leu491fs)	
80565678		Carriers from literature - data from papers only	10.1038/s41431-021-00977-9	1	c.1953+1G>C, splice donor	
82585307	*MCM8*_01	Outpatient clinic			c.2027A>T, p.(Tyr676Phe); c.1953+1G>C, splice donor	
**Biallelic *MCM9* (VUS)**						
31857966		Carriers from literature - data from papers only	10.1093/hmg/ddaa101	NOA-144		c.1301A>C, p.(Gln434Pro)
57170234		Carriers from literature - data from papers only	10.1007/s10815-021-02083-7	FS0054		c.1291A>G, p.(Met431Val); c.1157C>T, p.(Thr386Met)
58289086		Carriers from literature - data from papers only	10.1007/s10815-018-1349-4	FPOI38		c.1784C>G, p.(Thr595Arg); c.905-1G>T, splice acceptor
78493209	*MCM9*_09	Outpatient clinic				c.1642C>T, p.(Arg548Trp); c.152A>T, p.(Asn51Ile)
82082774	*MCM9*_09	Carriers from literature - updated data	10.1172/jci.insight.140698	011-69294-1		c.1642C>T, p.(Arg548Trp); c.152A>T, p.(Asn51Ile)
88176711	*MCM9*_08	Carriers from literature - updated data	10.1172/jci.insight.140698	MSS13-1961		c.3425A>G, p.(Lys1142Arg); c.1640T>C, p.(Leu547Pro)
**Biallelic *MCM9* (pathogenic or likely pathogenic)**						
08014933	*MCM9*_03	Carriers from literature - updated data	10.1016/j.cancergen.2015.10.001	III-4		c.672_673delinsC, p.(Glu225fs)
17276581	*MCM9*_04	Carriers from literature - updated data	10.1038/s41525-021-00242-4	IV-2		c.1483G>T, p.(Glu495*)
17419906		Carriers from literature - data from papers only	10.1515/jpem-2020-0590	P33		c.1732+2T>C, splice donor
30893196	*MCM9*_05	Carriers from literature - updated data	10.3390/jcm12030990	IV-2		c.394C>T, p.(Arg132*)
37139385		Carriers from literature - data from papers only	10.1016/j.ajhg.2014.11.002	AII-6		c.1732+2T>C, splice donor
40636194		Carriers from literature - data from papers only	10.1210/jc.2019-00248	BAB100 68		c.220C>T, p.(Arg74*)
42545284		Carriers from literature - data from papers only	10.1515/jpem-2020-0590	P32		c.1732+2T>C, splice donor

44283624	*MCM9*_04	Carriers from literature - updated data	10.1038/s41525-021-00242-4	IV-1		c.1483G>T, p.(Glu495*)
54365488	*MCM9*_06	Outpatient clinic				c.1720C>T, p.(Arg574*); c.1529-3C>A, splice donor
54601491		Carriers from literature - data from papers only	10.1111/cge.13803	POI-02		c.1473dup, p.(Thr492Tyrfs*4)
54840711	*MCM9*_05	Carriers from literature - updated data	10.3390/jcm12030990	IV-3		c.394C>T, p.(Arg132*)
67217467		Carriers from literature - data from papers only	10.1007/s10815-018-1349-4	FPOI24		c.1651C>T, p.(Gln551*)
69061260		Carriers from literature - data from papers only	10.1111/cge.13803	POI-03		c.1473dup, p.(Thr492Tyrfs*4)
74185426	*MCM9*_05	Carriers from literature - updated data	10.3390/jcm12030990	IV-5		c.394C>T, p.(Arg132*)
75503761		Carriers from literature - data from papers only	10.1210/jc.2019-00248	BAB943 5		c.394C>T, p.(Arg132*)
79778808		Carriers from literature - data from papers only	10.1210/jc.2016-2565			c.1651C>T, p.(Gln551*)
80899809	*MCM9*_05	Carriers from literature - updated data	10.3390/jcm12030990	IV-1		c.394C>T, p.(Arg132*)
80909502		Carriers from literature - data from papers only	10.1016/j.ajhg.2014.11.002	AII-4		c.1732+2T>C, splice donor
82941667		Carriers from literature - data from papers only	10.1016/j.ajhg.2014.11.002	BII-1		c.394C>T, p.(Arg132*)
91725927	*MCM9*_10	Outpatient clinic				c.394C>T, p.(Arg132*)
98796144	*MCM9*_03	Carriers from literature - updated data	10.1016/j.cancergen.2015.10.001	III-3		c.672_673delinsC, p.(Glu225fs)
17290073	*MCM9*_07	Outpatient clinic				c.820C>T, p.(Gln274*); c.2237_2238dup, p.(Phe747Ilefs*78)
**Monoallelic *MCM8* (VUS)**						
19246873	*MCM8*_04	Outpatient clinic			c.692T>A, p.(Ile231Lys)	
20049146		Carriers from literature - data from papers only	10.1172/JCI78473	IV-8	c.446C>G, p.(Pro149Arg)	
21357409		Carriers from literature - data from papers only	10.1186/s12920-020-00813-x	P32	c.839C>G, p.(Ser280Cys)	
22206931		Carriers from literature - data from papers only	10.1210/jc.2019-00248	BAP710 0	c.89A>C, p.(Lys30Thr); c.1330A>G, p.(Ile444Val)	
22444712		Carriers from literature - data from papers only	10.1210/jc.2019-00248		c.89A>C, p.(Lys30Thr)	
24757813		Carriers from literature - data from papers only	10.1016/j.fertnstert.2017.07.015	IV-3	c.482A>C, p.(His161Pro)	
27628395		Carriers from literature - data from papers only	10.1172/JCI78473	IV-2	c.446C>G, p.(Pro149Arg)	
28219291		Carriers from literature - data from papers only	10.1002/mgg3.1396	Proband 's sister	c.724T>C, p.(Cys242Arg); c.1334C>A, p.(Ala445Asp)	

28527992	*MCM8*_03	Outpatient clinic			c.482A>G, p.(His161Arg)	
28664918		Carriers from literature - data from papers only	10.1172/JCI78473	IV-5	c.446C>G, p.(Pro149Arg)	
33556074		Carriers from literature - data from papers only	10.1186/s12920-020-00813-x	P28	c.1445C>T, p.(Thr482Met)	
33783390	*MCM8*_04	Outpatient clinic			c.692T>A, p.(Ile231Lys)	
34898570		Carriers from literature - data from papers only	10.1210/jc.2016-2565		c.1577A>G, p.(Gln526Arg)	
35155778	*MCM8*_01	Outpatient clinic			c.2027A>T, p.(Tyr676Phe)	
35922003		Carriers from literature - data from papers only	10.1016/j.fertnstert.2017.07.015	V-8	c.482A>C, p.(His161Pro)	
37725555	*MCM8*_04	Outpatient clinic			c.692T>A, p.(Ile231Lys)	
39121178	*MCM8*_04	Outpatient clinic			c.692T>A, p.(Ile231Lys)	
45566358		Carriers from literature - data from papers only	10.1172/JCI78473	III-1	c.446C>G, p.(Pro149Arg)	
51426649		Carriers from literature - data from papers only	10.1210/jc.2019-00248		c.89A>C, p.(Lys30Thr); c.1330A>G, p.(Ile444Val)	
55019508		Carriers from literature - data from papers only	10.1016/j.fertnstert.2016.08.018	192	c.950A>T, p.(His317Leu); c.1801_1803del, p.(His601Arg)	
56285499		Carriers from literature - data from papers only	10.1016/j.fertnstert.2017.07.015	V-6	c.482A>C, p.(His161Pro)	
57000767		Carriers from literature - data from papers only	10.1210/jc.2016-2565		c.1561G>A, p.(Asp521Asn)	
64086299		Carriers from literature - data from papers only	10.1002/mgg3.1396	Proband	c.724T>C, p.(Cys242Arg); c.1334C>A, p.(Ala445Asp)	
65644624		Carriers from literature - data from papers only	10.1210/jc.2016-2565		c.482A>G, p.(His161Arg)	
70776442		Carriers from literature - data from papers only	10.1186/s12920-020-00813-x	P14	c.1445C>T, p.(Thr482Met)	
71560890	*MCM8*_02	Carriers from literature - updated data	10.1172/jci.insight.140698	MSS23-1939	c.2209G>A, p.(Ala737Thr)	
76635144		Carriers from literature - data from papers only	10.1016/j.fertnstert.2017.07.015	IV-4	c.482A>C, p.(His161Pro)	
80115707		Carriers from literature - data from papers only	10.1210/jc.2016-2565		c.1334G>A, p.(Arg445Gln)	
91398837		Carriers from literature - data from papers only	10.1172/JCI78473	III-2	c.446C>G, p.(Pro149Arg)	
93819210	*MCM8*_04	Outpatient clinic			c.692T>A, p.(Ile231Lys)	
99800629	*MCM8*_01	Outpatient clinic			c.2027A>T, p.(Tyr676Phe)	
**Monoallelic *MCM8* (pathogenic or likely pathogenic)**						

19352727	*MCM8*_01	Outpatient clinic			c.1953+1G>C, splice donor	
23670309		Carriers from literature - data from papers only	10.1002/mgg3.1165	III-1	c.351_354del, p.(Lys118fs)	
23830393		Carriers from literature - data from papers only	10.1136/jmedgenet-2014-102921	III-1	c.1470_1471insTA, p.(Leu491fs)	
35383705		Carriers from literature - data from papers only	10.1136/jmedgenet-2014-102921	IV-1	c.1954-1G>A, splice acceptor	
39108256		Carriers from literature - data from papers only	10.1210/jc.2019-00248		c.925C>T, p.(Arg309*)	
48121907		Carriers from literature - data from papers only	10.1136/jmedgenet-2014-102921	IV-5	c.1470_1471insTA, p.(Leu491fs)	
48419419		Carriers from literature - data from papers only	10.1136/jmedgenet-2014-102921	V-3	c.1954-1G>A, splice acceptor	
50513168		Carriers from literature - data from papers only	10.1210/jc.2019-00248		c.925C>T, p.(Arg309*)	
53121845	*MCM8*_05	Carriers from literature - updated data	10.1172/jci.insight.140698	LLS17	c.351_354del, p.(Lys118Glufs*5); c.414A>G, p.(Ile138Met)	
56752266		Carriers from literature - data from papers only	10.1002/mgg3.1165	III-2	c.351_354del, p.(Lys118fs)	
70137960		Carriers from literature - data from papers only	10.1136/jmedgenet-2014-102921	III-3	c.1470_1471insTA, p.(Leu491fs)	
70350057		Carriers from literature - data from papers only	10.3390/cancers13040929	AA3530	c.876-1del	
72640236		Carriers from literature - data from papers only	10.1210/clinem/dgaa155	III-2	c.925C>T, p.(Arg309*)	
85760408		Carriers from literature - data from papers only	10.1210/clinem/dgaa155	III-1	c.925C>T, p.(Arg309*)	
86022960	*MCM8*_01	Outpatient clinic			c.1953+1G>C, splice donor	
95528561		Carriers from literature - data from papers only	10.1136/jmedgenet-2014-102921	III-2	c.1470_1471insTA, p.(Leu491fs)	
95717575		Carriers from literature - data from papers only	10.1136/jmedgenet-2014-102921	IV-2	c.1954-1G>A, splice acceptor	
97627350		Carriers from literature - data from papers only	10.1210/clinem/dgaa155	IV-1	c.925C>T, p.(Arg309*)	
**Monoallelic *MCM9* (VUS)**						
06583901		Carriers from literature - data from papers only	10.1016/j.neurobiolaging.2021.12.004	III-4		c.739C>T, p.(Arg247Trp)
10277227	*MCM8_MCM9_01*	Carriers from literature - updated data	10.1172/jci.insight.140698	MSS2-1941	c.832C>T, p.(Arg278Cys)	c.3425A>G, p.(Lys1142Arg)
17364805		Carriers from literature - data from papers only	10.1210/jc.2016-2565			c.686T>G, p.(Val229Gly)
17724734		Carriers from literature - data from papers only	10.1016/j.fertnstert.2019.11.015	POI-1		c.1423C>T, p.(Leu475Phe)

26113606	*MCM9*_02	Carriers from literature - updated data	10.1172/jci.insight.140698	NA96-14		c.1915C>G, p.(Leu639Val)
36162360		Carriers from literature - data from papers only	10.1371/journal.pone.0240795	POI-25		c.1163C>A, p.(Thr388Asn)
37235179		Carriers from literature - data from papers only	10.1210/jc.2016-2565			c.1784C>G, p.(Thr595Arg)
38753115		Carriers from literature - data from papers only	10.1016/j.neurobiolaging.2021.12.004	IV-9		c.739C>T, p.(Arg247Trp)
48901681		Carriers from literature - data from papers only	10.1016/j.neurobiolaging.2021.12.004	III-8		c.739C>T, p.(Arg247Trp)
53809122		Carriers from literature - data from papers only	10.1002/humu.24057	F39		c.427C>T, p.(Arg143Trp)
54336601	*MCM9*_06	Outpatient clinic				c.1529-3C>A, splice donor
58397907		Carriers from literature - data from papers only	10.1016/j.neurobiolaging.2021.12.004	III-3		c.739C>T, p.(Arg247Trp)
63437136		Carriers from literature - data from papers only	10.1016/j.neurobiolaging.2021.12.004	III-1		c.739C>T, p.(Arg247Trp)
83280371		Carriers from literature - data from papers only	10.1186/s12920-020-00813-x	P26		c.1330G>C, p.(Val444Leu)
90757541		Carriers from literature - data from papers only	10.1210/jc.2016-2565			c.905-1G>T, splice acceptor
**Monoallelic *MCM9* (pathogenic or likely pathogenic)**						
00367545	*MCM9*_03	Carriers from literature - updated data	10.1016/j.cancergen.2015.10.001	III-2		c.672_673delinsC, p.(Glu225fs)
00369720		Carriers from literature - data from papers only	10.1515/jpem-2020-0590			c.1732+2T>C, splice donor
00882217		Carriers from literature - data from papers only	10.1016/j.ajhg.2014.11.002	AI-1		c.1732+2T>C, splice donor
02099761		Carriers from literature - data from papers only	10.1016/j.ajhg.2014.11.002	AI-2		c.1732+2T>C, splice donor
07793607	*MCM9*_03	Carriers from literature - updated data	10.1016/j.cancergen.2015.10.001	II-1		c.672_673delinsC, p.(Glu225fs)
14948286		Carriers from literature - data from papers only	10.1210/jc.2016-2565			c.2011G>T, p.(Glu671*)
15964832		Carriers from literature - data from papers only	10.1210/jc.2019-00248			c.220C>T, p.(Arg74*)
24996014		Carriers from literature - data from papers only	10.1016/j.ajhg.2014.11.002	BII-2		c.394C>T, p.(Arg132*)
25080587	*MCM9*_03	Carriers from literature - updated data	10.1016/j.cancergen.2015.10.001	III-1		c.672_673delinsC, p.(Glu225fs)
26472597		Carriers from literature - data from papers only	10.1016/j.ajhg.2014.11.002	BI-1		c.394C>T, p.(Arg132*)
29508735	*MCM9*_01	Carriers from literature - updated data	10.1172/jci.insight.140698	NA41-1		c.1987dup, p.(Ser663Phefs*36)
35173643		Carriers from literature - data from papers only	10.1016/j.ajhg.2014.11.002	AII-3		c.1732+2T>C, splice donor

40559303	*MCM9*_04	Carriers from literature - updated data	10.1038/s41525-021-00242-4	IV-3		c.1483G>T, p.(Glu495*)
41229278	*MCM9*_03	Carriers from literature - updated data	10.1016/j.cancergen.2015.10.001	II-2		c.672_673delinsC, p.(Glu225fs)
42152259		Carriers from literature - data from papers only	10.1210/jc.2019-00248			c.220C>T, p.(Arg74*)
42750756		Carriers from literature - data from papers only	10.1210/jc.2019-00248			c.394C>T, p.(Arg132*)
49294952	*MCM9*_04	Carriers from literature - updated data	10.1038/s41525-021-00242-4	III-2		c.1483G>T, p.(Glu495*)
58731687		Carriers from literature - data from papers only	10.1016/j.ajhg.2014.11.002	AII-2		c.1732+2T>C, splice donor
62507727		Carriers from literature - data from papers only	10.1210/jc.2019-00248			c.394C>T, p.(Arg132*)
71823158	*MCM9*_03	Carriers from literature - updated data	10.1016/j.cancergen.2015.10.001	I-1		c.672_673delGGinsC, p.(Glu225fs)
76457125		Carriers from literature - data from papers only	10.1016/j.ajhg.2014.11.002	BII-4		c.394C>T, p.(Arg132*)
77887094		Carriers from literature - data from papers only	10.1016/j.ajhg.2014.11.002	BI-2		c.394C>T, p.(Arg132*)
82416835		Carriers from literature - data from papers only	10.1515/jpem-2020-0590			c.1732+2T>C, splice donor
92396046	*MCM9*_04	Carriers from literature - updated data	10.1038/s41525-021-00242-4	IV-4		c.1483G>T, p.(Glu495*)
94130086		Carriers from literature - data from papers only	10.1210/jc.2016-2565			c.2011G>T, p.(Glu671*)
95282910	*MCM9*_04	Carriers from literature - updated data	10.1038/s41525-021-00242-4	III-3		c.1483G>T, p.(Glu495*)
98962558	*MCM9*_05	Carriers from literature - updated data	10.3390/jcm12030990	IV-6		c.394C>T, p.(Arg132*)
99284187	*MCM9*_06	Outpatient clinic				c.1720C>T, p.(Arg574*)
59356494	*MCM9*_07	Outpatient clinic				c.820C>T, p.(Gln274*)
63108002	*MCM9*_07	Outpatient clinic				c.2237_2238dup, p.(Phe747Ilefs*78)

Table S3. Driver mutations in CRC-related genes detected in tumors from our case series.

Sample	HUGO symbol	Driver mutation	Variant classification
P1_1T	*ARID1A*	c.5452C>T, p.(Gln1818*)	Nonsense mutation
	DICER1	c.1966C>T. p.(Arg656*)	Nonsense mutation
	EP300	-	Splice site
	GRIN2A	c.601C>T, p.(Gln201*)	Nonsense mutation
	LRP1B	c.5395C>T, p.(Gln1799*)	Nonsense mutation
	LRP1B	-	Splice site
	LRP1B	c.1453insA, p.(Gly485Trpfs*)	Frame shift ins
	MYH9	c.5014G>T, p.(Glu1672*)	Nonsense mutation
	SMARCA4	c.580C>T, p.(Gln194*)	Nonsense mutation
P2_2T	*CYLD*	c.686delT, p.(Pro229Leufs*)	Frame shift del
P4_7T	*CTNNB1*	c.110C>A, p.(Ser37Tyr)	Missense mutation
P4_8T	*CTNNB1*	c.110C>A, p.(Ser37Tyr)	Missense mutation
P4_8T	*KRAS*	c.35G>A, p.(Gly12Asp)	Missense mutation
P5_10T	*AMER1*	c.3329C>A, p.(Ser1110*)	Nonsense mutation
	FBXW7	c.1393C>T, p.(Arg465Cys)	Missense mutation
	ROBO2	c.727G>T, p.(Glu243*)	Nonsense mutation
	TP53	-	Splice site
P5_9T	*APC*	-	Splice site
	APC	c.2767A>T, p.(Arg923*)	Nonsense mutation
	LRP1B	c.6064G>T, p.(Gly2022*)	Nonsense mutation
P6_11T	*APC*	c.2674G>T, p.(Glu892*)	Nonsense mutation
	ROBO2	p.(Thr470Argfs*)	Frame shift del
P7_12T	*FBXW7*	c.37C>T, p.(Arg13*)	Nonsense mutation
	TP53	c.524G>A, p.(Arg175His)	Missense mutation
	TP53	-	Splice site

CRC, colorectal cancer

Supplemental references

1. Golubicki M, Bonjoch L, Acuna-Ochoa JG, et al. Germline biallelic Mcm8 variants are associated with early-onset Lynch-like syndrome. JCI Insight. 2020;5(18).
2. Goldberg Y, Halpern N, Hubert A, et al. Mutated MCM9 is associated with predisposition to hereditary mixed polyposis and colorectal cancer in addition to primary ovarian failure. Cancer Genet. 2015;208(12):621-4.
3. Goldberg Y, Aleme O, Peled-Perets L, et al. MCM9 is associated with germline predisposition to early-onset cancer-clinical evidence. NPJ Genom Med. 2021;6(1):78.
4. Potorac I, Laterre M, Malaise O, et al. The Role of MCM9 in the Etiology of Sertoli Cell-Only Syndrome and Premature Ovarian Insufficiency. J Clin Med. 2023;12(3).
5. Richards CS, Bale S, Bellissimo DB, et al. ACMG recommendations for standards for interpretation and reporting of sequence variations: Revisions 2007. Genet Med. 2008;10(4):294-300.
6. Richards S, Aziz N, Bale S, et al. Standards and guidelines for the interpretation of sequence variants: a joint consensus recommendation of the American College of Medical Genetics and Genomics and the Association for Molecular Pathology. Genet Med. 2015;17(5):405-24.
7. Wyrwoll MJ, Kockerling N, Vockel M, et al. Genetic Architecture of Azoospermia-Time to Advance the Standard of Care. Eur Urol. 2023;83(5):452-62.
