# Supplementary material for: Clinical syndromes linked to biallelic germline variants in MCM8 and MCM9
Source: HGG Adv. 2025 Jul 18;6(4):100480. doi: 10.1016/j.xhgg.2025.100480 (PMC12361757; doi:10.1016/j.xhgg.2025.100480)
Supplement: Document S2. Article plus supplemental information [file mmc3.pdf]

HGG Advances ARTICLE

Clinical syndromes linked to biallelic germline variants in *MCM8* and *MCM9*

Noah C. Helderman,[1] Ting Yang,[2,3,4] Claire Palles,[5] Diantha Terlouw,[6] Hailiang Mei,[7] Ruben H.P. Vorderman,[7] Davy Cats,[7] Marcos Díaz-Gay,[2,3,4,8] Marjolijn C.J. Jongmans,[9,10] Ashwin Ramdien,[1] Irma van de Beek,[11] Thomas F. Eleveld,[9] Andrew Green,[12] Frederik J. Hes,[13] Marry M. van den Heuvel-Eibrink,[9,14] Annelore Van Der Kelen,[13] Sabine Kliesch,[15] Roland P. Kuiper,[9] Inge M.M. Lakeman,[1] Lisa E.E.L.O. Lashley,[16] Leendert H.J. Looijenga,[9,17] Manon S. Oud,[18] Johanna Steingröver,[19] Yardena Tenenbaum-Rakover,[20] Carli M. Tops,[1] Frank Tüttelmann,[19] Richarda M. de Voer,[21] Dineke Westra,[21] Margot J. Wyrwoll,[22] Mariano Golubicki,[23] Marina Antelo,[23] Laia Bonjoch,[24] Mariona Terradas,[25] Laura Valle,[25] Ludmil B. Alexandrov,[2,3,4] Hans Morreau,[6] Tom van Wezel,[6] Sergi Castellví-Bel,[24,27] Yael Goldberg,[26,27] and Maartje Nielsen[1,28,*]

Summary

MCM8 and *MCM9* are newly proposed cancer predisposition genes, linked to polyposis and early-onset cancer, in addition to their previously established association with hypogonadism. Given the uncertain range of phenotypic manifestations and unclear cancer risk estimates, this study aimed to delineate the molecular and clinical characteristics of biallelic germline *MCM8/MCM9* variant carriers. We found significant enrichment of biallelic *MCM9* variants in individuals with colonic polyps (odds ratio [OR] 6.51, 95% confidence interval [CI] 1.24–34.11, $p = 0.03$), rectal polyps (OR 8.40, 95% CI 1.28–55.35, $p = 0.03$), and gastric cancer (OR 27.03, 95% CI 2.93–248.5; $p = 0.004$) in data from the 100000 Genomes Project, compared to controls. No similar enrichment was found for biallelic *MCM8* variants or in the 200000 UK Biobank. Likewise, in our case series, which included 26 *MCM8* and 28 *MCM9* variant carriers, we documented polyposis, gastric cancer, and early-onset colorectal cancer (CRC) in *MCM9* carriers but not in *MCM8* carriers. Moreover, our case series indicates that beyond hypogonadism, biallelic *MCM8* and *MCM9* variants are associated with early-onset germ cell tumors (occurring before age 15). Tumors from *MCM8/MCM9* variant carriers predominantly displayed clock-like mutational processes, without evidence of DNA repair deficiency-associated signatures. Collectively, our data indicate that biallelic *MCM9* variants are associated with polyposis, gastric cancer, and early-onset CRC, while both biallelic *MCM8* and *MCM9* variants are linked to hypogonadism and the early development of germ cell tumors. These findings underscore the importance of including *MCM8/MCM9* in diagnostic gene panels for certain clinical contexts and suggest that biallelic carriers may benefit from cancer surveillance.

Introduction

The identification of cancer predisposition syndromes plays a crucial role in preventing and surveilling malignancies at an early stage in affected individuals. Nevertheless, a significant proportion of familial cancer cases lack a clear explanation.[1] This poses challenges in developing personalized surveillance programs and highlights the

[1]Department of Clinical Genetics, Leiden University Medical Center, Leiden, the Netherlands; [2]Department of Cellular and Molecular Medicine, University of California, San Diego, La Jolla, CA 92093, USA; [3]Department of Bioengineering, University of California, San Diego, La Jolla, CA 92093, USA; [4]Moores Cancer Center, University of California, San Diego, La Jolla, CA 92037, USA; [5]Institute of Cancer and Genomic Sciences, University of Birmingham, Birmingham, UK; [6]Department of Pathology, Leiden University Medical Center, Leiden, the Netherlands; [7]Sequencing Analysis Support Core, Department of Biomedical Data Sciences, Leiden University Medical Center, Leiden, the Netherlands; [8]Digital Genomics Group, Structural Biology Program, Spanish National Cancer Research Center (CNIO), 28029 Madrid, Spain; [9]Princess Maxima Center for Pediatric Oncology, Utrecht, the Netherlands; [10]Department of Genetics, University Medical Center Utrecht, University of Utrecht, Utrecht, the Netherlands; [11]Department of Clinical Genetics, The Netherlands Cancer Institute, Amsterdam, the Netherlands; [12]Department of Clinical Genetics, Children's Health Ireland (CHI) at Crumlin, Dublin, Ireland; [13]Vrije Universiteit Brussel (VUB), Universitair Ziekenhuis Brussel (UZ Brussel), Clinical Sciences, Research Group Genetics, Reproduction, and Development, Centre for Medical Genetics, Brussels, Belgium; [14]Division of Childhealth, University Medical Center Utrecht, Utrecht, the Netherlands; [15]Centre of Reproductive Medicine and Andrology, Department of Clinical and Surgical Andrology, University Hospital Münster, Münster, Germany; [16]Department of Obstetrics and Gynecology, Leiden University Medical Center, Leiden, the Netherlands; [17]Department of Pathology, University Medical Center Utrecht, Utrecht, the Netherlands; [18]Department of Human Genetics, Donders Institute for Brain, Cognition, and Behaviour, Radboud University Medical Center, Nijmegen, the Netherlands; [19]Centre of Medical Genetics, Institute of Reproductive Genetics, University and University Hospital of Münster, Münster, Germany; [20]Pediatric Endocrinology, Clalit Health Services, Afula, Israel; The Ruth and Bruce Rappaport Faculty of Medicine, Technion, Haifa, Israel; [21]Department of Human Genetics, Radboud University Medical Center, Radboud Institute for Molecular Life Sciences, Nijmegen, the Netherlands; [22]Centre for Regenerative Medicine, Institute for Stem Cell Research, School of Biological Sciences, University of Edinburgh, Edinburgh, UK; [23]Oncology Section and Molecular Biology Laboratory, Hospital of Gastroenterology "Dr. C.B. Udaondo", Buenos Aires, Argentina; [24]Gastroenterology, Fundació de Recerca Clínic Barcelona-Institut d'Investigacions Biomèdiques August Pi I Sunyer (FRCB-IDIBAPS), CIBEREHD, Universitat de Barcelona, Clínic Barcelona, Barcelona, Spain; [25]Hereditary Cancer Program, Catalan Institute of Oncology, Oncobell Program, IDIBELL, CIBERONC, Hospitalet de Llobregat, Barcelona, Spain; [26]Raphael Recanati Genetic Institute, Rabin Medical Center-Beilinson Hospital, Petah Tikva, Israel
[27]These authors contributed equally
[28]Lead contact
*Correspondence: m.nielsen@lumc.nl
https://doi.org/10.1016/j.xhgg.2025.100480.
© 2025 The Author(s). Published by Elsevier Inc. on behalf of American Society of Human Genetics.
This is an open access article under the CC BY license (http://creativecommons.org/licenses/by/4.0/).

urgency of exploring and identifying novel cancer predisposition genes.

The minichromosome maintenance 8 homologous recombination repair factor (*MCM8*; NM_032485.6, ENST00000610722.4, OMIM: 608187) and minichromosome maintenance 9 homologous recombination repair factor (*MCM9*; NM_017696.3, ENST00000619706.5, OMIM: 610098) genes are two recently suggested cancer predisposition genes.[2–4] The proteins encoded by these genes form a helicase hexameric complex that is likely involved in DNA replication and the initiation of DNA replication,[5–9] meiosis,[7,10–13] homologous recombination,[14–20] and mismatch repair (MMR).[4,19,21]

Following their significant association with primary ovarian insufficiency (POI; HP:0008209),[2–4,22–40] biallelic germline variants of *MCM8/MCM9* were first linked to cancer in several families with polyposis (HP:0200063) and early-onset colorectal cancer (CRC; HP:0003003).[2–4] Subsequently, there have been reports of individuals with CRC carrying a monoallelic *MCM8/MCM9* variant,[2–4] as well as reports describing mono- and biallelic germline *MCM8/MCM9* variants in individuals with other nonmalignant pathologies, including short stature (HP:0004322),[29,34,35,38,39] delayed puberty (HP:0000823),[22,23,26,28,33,38–40] hypothyroidism (HP:0000821),[22,28] and absent or infantile uteri/ovaries.[22,23,26,27,29,31,33,35,37–40]

Due to the limited number of families with biallelic germline *MCM8/MCM9* variants described so far, the complete spectrum of phenotypic manifestations and accurate cancer risk estimates remains uncertain. As a result, the incorporation of the *MCM8/MCM9* genes into diagnostic gene panels is not widespread, and the respective syndrome(s) associated with both genes could easily be missed. This study, therefore, sought to delineate the molecular and clinical features of biallelic germline *MCM8/MCM9* variants and to establish recommendations for the clinical management of variant carriers.

Subjects, material, and methods

Ethics statement

This study was approved by the local institutional review board (IRB) and biobank committee of the Leiden University Medical Center in the Netherlands (protocol B18.007). Storage and management of clinical and molecular data and participant samples from our case series were supervised by the Leiden University Medical Center. Participant samples were handled according to the medical ethical guidelines described in the code of conduct for responsible use of human tissue in the context of health research (Federation of Dutch Medical Scientific Societies). Samples were coded/anonymized, and all individuals provided written informed consent for the use of tissue and data.

Population-based cohorts

Estimation of population allele and biallelic carrier frequencies in gnomAD version 2.1.1

The gnomAD version 2.1.1 database (https://gnomad.broadinstitute.org/), which comprises 125,748 exome sequences and 15,708 whole-genome sequences from a total of 141,456 unrelated individuals, was accessed in May 2023 to estimate the population allele frequencies (AFs) and biallelic carrier frequencies of *MCM8* and *MCM9* variants across diverse populations. We analyzed predicted loss of function (pLoF) variants—including splice acceptor, splice donor, frameshift, and stop gained variants—as well as missense variants, using variant annotations based on the Ensembl Variant Effect Predictor (VEP) classification (Figure 1)[41] Population AFs were derived from the combined exome and genome dataset and expressed as the number of cases per 100,000 individuals, unless stated otherwise. Biallelic carrier frequencies were estimated using the gnomAD variant co-occurrence tool (https://gnomad.broadinstitute.org/variant-cooccurrence?dataset=gnomad_r2_1), which enables phasing of variants and is restricted to the exome dataset. Biallelic carriers were defined as individuals harboring either homozygous or compound heterozygous variants in *MCM8* or *MCM9*. For compound heterozygosity, only individuals with variants in *trans* (on different alleles) were included, while those with variants in *cis* (on the same allele) were excluded from the analysis.

Identification of carriers and variant enrichment analysis in 200000 UK Biobank and 100000 Genomes Project datasets

Germline variants in *MCM8* and *MCM9* were identified from the 100000 Genomes Project (project code 1142, version 17) and the 200000 exomes release of the UK Biobank (project code 86977, released on November 17, 2021). Variants were annotated using VEP version 107.[41] We retained missense variants with a Combined Annotation-Dependent Depletion (CADD)[42] score $\geq$20 and a deleterious Condel score,[43] as well as pLoF variants (including splice acceptor, splice donor, frameshift, and stop gained variants), provided their AF was <1% in gnomAD version 2.1.1 (Figure 1). The impact of variants on the canonical transcripts was reported for *MCM8* (ENST00000610722.4) and *MCM9* (ENST00000619706.5).

The *International Classification of Diseases, 10th Revision* (ICD-10) codes from participants' diagnosis information (Participant Explorer in 100000 Genomes Project, field ID 41270 in 200000 UK Biobank), along with the *International Classification of Diseases for Oncology* (ICD-O) codes obtained from cancer histology and behavior fields (field ID 40011 and 40012 in 200000 UK Biobank), as shown in Table S1, were searched to identify participants with phenotypes associated with *MCM8/MCM9* variants, as selected based on the literature[44] as well as our case series.

We conducted case-control tests to assess whether potentially pathogenic biallelic (homozygous or compound heterozygous) *MCM8/MCM9* variants were

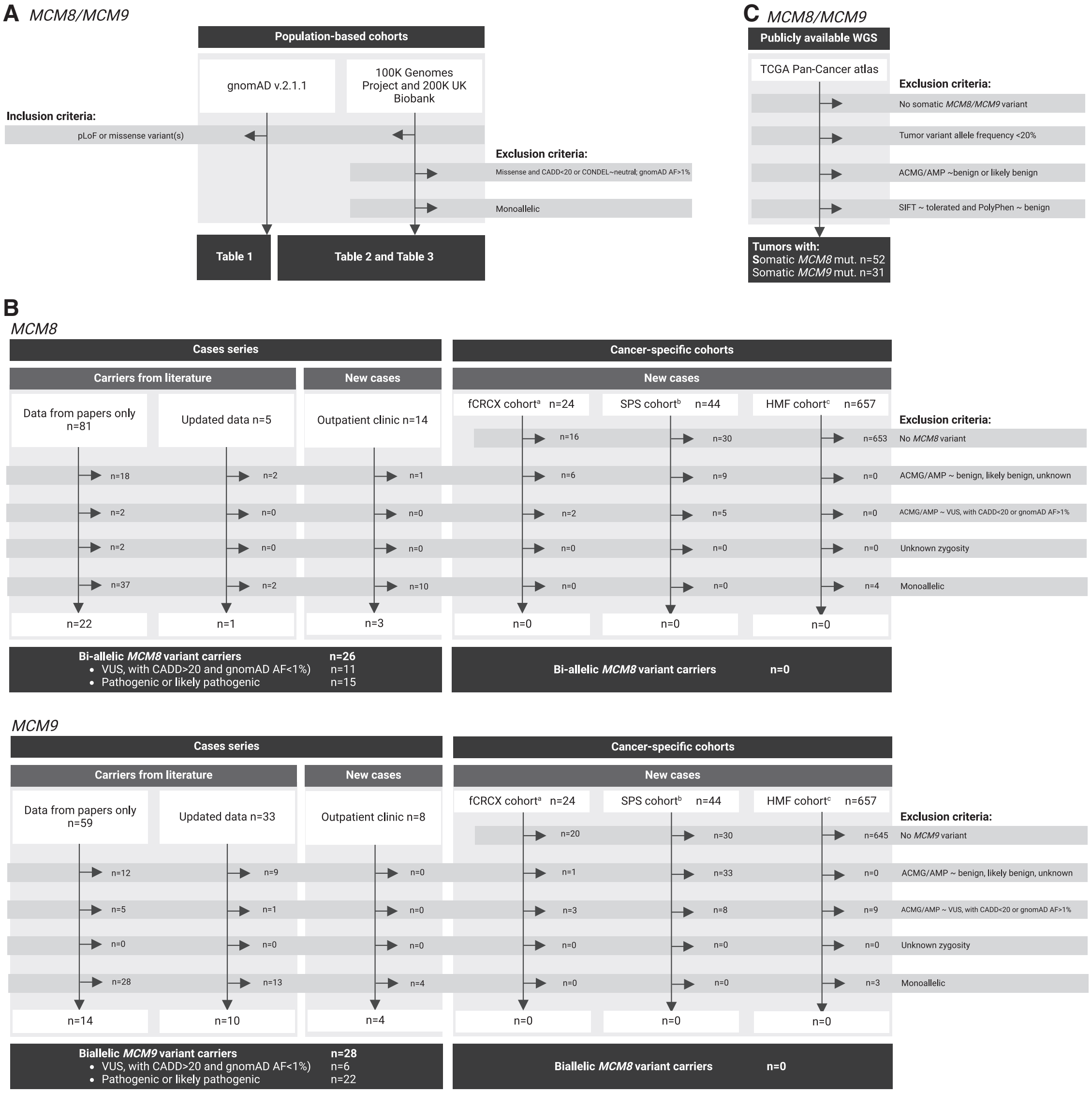

Figure 1. Flowchart of study approach

Pathogenicity-based filtering of (A) population-based cohorts, (B) our case series and cancer-specific cohorts, and (C) TCGA Pan-Cancer atlas dataset. [a]fCRCX cohort comprised 24 CRC-affected members of 16 Amsterdam-positive non-polyposis CRC families; [b]SPS cohort comprised 44 unrelated serrated polyposis families; [c]HMF cohort comprised 632 metastasized CRCs and 25 metastasized ECs. Tumors from TCGA Pan-Cancer Atlas were selected based on the presence of somatic *MCM8*/*MCM9* variants and are not related to germline variant carriers. ACMG/AMP, American College of Medical Genetics and Genomics; AF, allele frequency; CADD, Combined Annotation-Dependent Depletion; CRC, colorectal cancer; EC, endometrial cancer; pLoF, predicted loss of function; VUS, variant of uncertain significance.

enriched in participants with the phenotypes of interest compared to a control cohort. A total of 15,091 controls were identified from the 100000 Genomes Project dataset and 90,897 from the 200000 UK Biobank dataset. Controls were selected based on the absence of personal or family history of common cancers and any phenotypes listed in Table S1. To account for differences in age and ethnicity between cases and controls, association testing was performed using PLINK version 1.9, adjusting for both variables. Sex was included as a covariate in all analyses, except for breast cancer, endometrial cancer, and female infertility, where only female controls were considered.

Case series

First, we identified *MCM8/MCM9* variant carriers through multiple channels. On August 1, 2023, we conducted a comprehensive literature search for "MCM8" and "MCM9" in the NCBI PubMed database. This strategy yielded 116 studies discussing *MCM8* and 75 studies discussing *MCM9*. We included all studies in English and carefully examined them for any descriptions of *MCM8/MCM9* variant carriers. We excluded (systematic) reviews to avoid duplicate participant data. Participant data were sourced from the papers themselves[22–32,34,38–40,45–50] or updated data were obtained from the first or corresponding authors upon request.[2–4,51]

Second, as part of the European Reference Network for all participants with one of the rare genetic tumor risk syndromes (GENTURIS) initiative,[52] we identified *MCM8/MCM9* variant carriers not previously documented through outpatient clinics at various institutes across Europe. Participant data were sourced from genetic practitioners or retrieved from health records.

Pathogenicity-based filtering and classification of the identified MCM8/MCM9 *variant carriers*

The *MCM8/MCM9* variants identified in the case series and cancer-specific cohorts (see next section) were filtered based on their predicted pathogenicity (Figure 1). Initially, we annotated the *MCM8/MCM9* variants using the guidelines from the American College of Medical Genetics and Genomics (ACMG) and the Association for Molecular Pathology (AMP) for variant interpretation,[53,54] along with the CADD scoring[42] and gnomAD version 2.1.1 AF, accessed through Franklin.[55] We excluded from the analysis (1) carriers of benign or likely benign *MCM8/MCM9* variants and (2) variants of uncertain significance (VUS) with a CADD score <20 or a gnomAD AF higher than 1%.

Individuals with homozygous or compound heterozygous variants that met the pathogenicity-based filtering criteria were considered to be biallelic carriers. Conversely, compound heterozygous carriers with one variant meeting the criteria and one that did not were categorized as monoallelic carriers. Additionally, compound heterozygous carriers with a pathogenic or likely pathogenic variant and a VUS that met the pathogenicity-based filtering criteria were included in the pathogenic or likely pathogenic group (i.e., as biallelic carriers).

Cancer-specific cohorts

We ascertained several cancer-specific cohorts to search for variant carriers with cancer phenotypes associated with germline *MCM8/MCM9* variants. These included 44 nonrelated serrated polyposis patients (SPS cohort)[56] and 24 cancer-affected members of 16 nonpolyposis CRC families (fCRCX cohort) from which germline whole-exome sequencing (WES) data was available for the analysis of single-nucleotide variants (SNVs) and insertion or deletion (indel) mutations, as well as 632 metastasized CRCs and 25 metastasized endometrial carcinomas with available germline and tumor whole-genome sequencing (WGS) data, accessible upon request by the Hartwig Medical Foundation database (reference no. HMF-DR-288; https://www.hartwigmedicalfoundation.nl/). The identified *MCM8/MCM9* variant carriers were filtered and classified based on pathogenicity using the same criteria applied to the *MCM8/MCM9* variant carriers from the case series, as detailed in the previous section (Figure 1).

Tumor DNA analysis

DNA sequencing and bioinformatic analysis of tumors from the case series

Participants and samples. To explore single-base substitution (SBS) mutational signatures potentially associated with *MCM8/MCM9* deficiency, DNA was obtained from formalin-fixed paraffin-embedded (FFPE) tumor tissue from the following individuals of our case series: 1 individual (1 tumor) with biallelic *MCM8* variants, 2 individuals (5 tumors) with monoallelic *MCM8* variants, 3 individuals (8 tumors) with biallelic *MCM9* variants, and 1 individual (1 tumor) with monoallelic *MCM8* and *MCM9* variants.

Sample preparation and molecular evaluation. DNA extraction from FFPE tissue blocks was conducted using the NucleoSpin DNA FFPE XS kit (Machery-Nagel, Düren, Germany), and DNA concentrations were quantified using the Qubit Meter dsDNA High Sensitivity kit (Thermo Fisher Scientific, Waltham, MA). WGS or WES was performed specifically for the purpose of this study using the NovaSeq 6000 Sequencing System (Illumina, San Diego, CA).

Somatic mutation calling. FASTQ files were aligned to the human genome build GRCh38.d1.vd1 using the Burrows-Wheeler Aligner (BWA-MEM, version 0.7.17).[57] Picard MarkDuplicates (GATK version 4.1.4.1) (Picard Toolkit, http://broadinstitute.github.io/picard; Broad Institute, Cambridge, MA) was applied to mark all duplicated reads.[58] SBS were identified using Mutect2 (GATK version 4.1.4.1),[59] VarScan (version 2.4.3),[60] MuSE (version 1.0),[61] and Strelka (version 2.9.10)[62] and filtered by variant caller confidences scores. Only variants that were called from at least two of these four callers were selected for the following mutational signature analysis and additional filtering based on their mutation confidence scores was applied: tumor logarithm of the odds score $\geq$ 10 (Mutect2) and SomaticEVS $\geq$ 13 (Strelka2). Samples with no matched germline sequencing data (10 out of 15 samples) were applied only to Mutect2 for variant calling under tumor-only mode.

Driver mutation identification. To identify potential driver mutations, we applied three complementary approaches to both SBSs and indels.

(1) We matched mutations to known driver events from The Cancer Genome Atlas (TCGA) MC3 study[63] by aligning them based on protein position and amino acid change. To increase specificity, only mutations flagged in at least two of the following categories in the master driver mutation sheet for colon adenocarcinoma and rectal adenocarcinoma were retained: "New_Linear (cancer-focused) flag," "New_Linear (functional) flag," and "New_3D mutational hotspot flag."
(2) We identified truncating mutations—nonsense, frameshift, or splice-site changes—in genes annotated as tumor suppressors, by cross-referencing the 82 IntOGen driver genes[64] with the COSMIC Cancer Gene Census[65] to determine tumor suppressor gene classification.
(3) We included missense mutations in any of the 82 IntOGen driver genes if they were annotated as "oncogenic" or "likely oncogenic" by OncoKB (version 3.4.1).

Mutational signature analysis. Mutational signature assignment was performed using SigProfilerAssignment (version 0.0.32)[66] based on the COSMIC (version 3.3) SBS and small insertion and deletion (ID) reference signatures.[67–71] Treatment-associated signatures (SBS11, SBS25, SBS31, SBS32, SBS35, SBS86, SBS87, SBS90, and SBS99) were excluded from all samples before signature assignment (using the *exclude_signature_subgroups* option), except for sample ID P8_33A, who had history of neoadjuvant chemotherapy treatment.

Tumor mutational burden. Tumor mutational burden (TMB) in coding regions was calculated by intersecting filtered VCF files with coding exonic regions defined by the Agilent GRCh38 exome capture kit (no_overlap_CCDS_CodingExons_33M.bed). The total number of somatic mutations within these regions was summed and normalized to the target region size (~33 Mb) to yield TMB values expressed as the number of somatic mutations per megabase.

Total copy-number identification. Total copy-number analysis was performed using CNVkit (version 0.9.8)[72] on both WGS and WES data, which were processed separately.

Bioinformatic analysis of publicly available WGS data

To further evaluate potential SBS mutational signatures associated with *MCM8/MCM9* deficiency, we analyzed tumor WGS data from two publicly available sources. First, we examined tumor data from cases with germline monoallelic *MCM8/MCM9* variants from the HMF cancer-specific cohort, as described earlier. Second, we evaluated tumor data from TCGA Pan-Cancer Atlas, accessed through cBioPortal for Cancer Genomics (https://www.cbioportal.org/) between February and April 2023.

For TCGA Pan-Cancer Atlas samples, tumors from any cancer type were selected based on the presence of somatic *MCM8/MCM9* variant(s) that met the following criteria: (1) a tumor variant AF of ≥20% and (2) classified as pathogenic or likely pathogenic or as a VUS according to the ACMG/AMP recommendations for variant interpretation.[53,54] Additionally, variants were excluded in case they were assessed as tolerated by the Sorting Intolerant from Tolerant score[73] and deemed benign by the PolyPhen score (Figure 1).[74]

In both the HMF and TCGA WGS datasets, SBS mutational signatures were identified by fitting the counts of SNVs per 96 tri-nucleotide context to the COSMIC version 3.3 reference mutational signatures[75] using the MutationalPatterns tool.[76]

Statistical analysis

Clinical data were collected using Castor Electronic Data Capture (https://castoredc.com). Figures were created, and statistical analysis was performed using RStudio version 2022.02.3+492 (Team R, Integrated Development for R, Boston, MA, 2022) or PLINK version 1.9.

Results

Population-based cohorts

Individuals with (biallelic) germline MCM8/MCM9 *variants are rare in gnomAD version 2.1.1*

The occurrence of pLoF variants of *MCM8* in gnomAD (version 2.1.1) was 1.4 individuals per 100,000 persons across all populations, with the highest prevalence (5.5 individuals per 100,000 persons) in the African/African American population (Table 1). Regarding *MCM9*, the prevalence of a pLoF variant was 2.5 individuals per 100,000 persons across all populations, with the highest prevalence (5.7 individuals per 100,000 persons) found in the European Finnish population. The prevalence of missense *MCM8* and *MCM9* variants was 462.4 and 1,173.3 individuals per 100,000 persons, respectively. Twenty-three individuals (0.02%) were identified as biallelic carriers of missense variants or more severe mutations of the *MCM8* gene (Table 1). With respect to the *MCM9* gene, 22 (0.02%) individuals were predicted to be biallelic carriers, including 21 carriers of missense variants or worse and one carrier of a homozygous pLoF variant.

Biallelic MCM9 *variant carriers in the 100000 Genomes Project have an increased risk of polyposis and gastric cancer, while no enrichment was observed for biallelic* MCM8 *variants or in the 200000 UK Biobank dataset*

In the 100000 Genomes Project, we identified 51 biallelic carriers (21 homozygous and 30 compound heterozygous) and 2,782 monoallelic carriers of pLoF or predicted deleterious missense variants in the *MCM8* gene. Moreover, we found 64 biallelic carriers (21 homozygous and 43 compound heterozygous) and 3,166 monoallelic carriers of pLoF or predicted deleterious missense variants in the *MCM9* gene. Among the 51 biallelic *MCM8* variant carriers in the 100000 Genomes Project, 2 individuals (3.9%) had CRC, 3 (5.9%) had colonic polyps, 3 (5.9%) had colonic adenomas, 3 (5.9%) had rectal polyps, 2 (3.9%) had hypothyroidism, and 5 (9.8%) had breast cancer. Additionally, 1 individual (2.0%) had epilepsy, 1 had endometrial cancer, 1 had short stature, and 1 experienced delayed puberty. Among the 64 biallelic *MCM9* variant carriers in the 100000 Genomes Project, 3 individuals (4.7%) had CRC, 2 (3.1%) had colonic polyps, 2 (3.1%) had colonic adenomas, 2 (3.1%) had rectal polyps, 3 (4.7%) had hypothyroidism, 5 (7.8%) had breast cancer, and 2 (3.1%) had epilepsy. Additionally, 1 individual (1.6%) had melanoma, 1 had gastric cancer, and 1 had endometrial cancer. While no significant enrichment of biallelic *MCM8* pLoF or predicted deleterious missense variants were observed for any of these phenotypes compared to controls, we did observe significant associations between biallelic *MCM9* pLoF or predicted deleterious missense variants and colonic polyps (odds ratio [OR] 6.51, 95% confidence interval [CI] 1.24–34.11, $p = 0.03$), rectal polyps (OR 8.40, 95% CI 1.28–55.35, $p = 0.03$), and gastric cancer (OR 27.03, 95% CI 2.93–248.5, $p = 0.004$) (Table 2).

Table 1. Population allele and biallelic carrier frequencies in gnomAD version 2.1.1

Population		# of variant carriers per 100,000 persons[a,b]			
		MCM8		*MCM9*	
		pLoF (n=83)	missense (n=490)	pLoF (n=55)	missense (n=556)
All		1.4	462.4	2.5	1173.3
African/African American		5.5	1058.3	2.6	1223.8
Latino/Admixed American		1.0	520.4	2.3	1142.6
Ashkenazi Jewish		0.5	362.7	0.7	1218.7
East Asian		0.6	757.4	0.9	1065.6
European (Finnish)		0.6	266.3	5.7	1127.8
European (non-Finnish)		0.8	303.9	2.2	1202.8
South Asian		0.9	594.9	1.7	1151.3
Other		0.7	379.2	2.2	1182.0
Zygosity		# of variant carriers per 125,748 persons[c,d]			
		MCM8		*MCM9*	
Compound heterozygous[e]					
	pLof + pLof	0[f]		0[g]	
	missense or pLoF + missense or pLoF	1[h]		9[i]	
Homozygous					
	pLoF	0		1	
	missense or pLoF	22		13	

AF, allele frequency; gnomAD, Genome Aggregation Database; pLoF, predicted loss of function.
[a]Population AF of *MCM8/MCM9* variants calculated based on the gnomAD (version 2.1.1) database, accessed through https://gnomad.broadinstitute.org/ in May 2023.
[b]Color intensity of each cell is proportional to the population AF, in relation to the population AFs of cells from the same column.
[c]Data were extracted from the gnomAD version 2.1.1 database and were based on exomes only ($n = 125{,}748$). The gnomAD database was accessed through https://gnomad.broadinstitute.org/ in May 2023.
[d]Although highly uncommon, there is a possibility that an individual may be categorized in both the compound heterozygous group and the homozygous group. This situation arises when the individual carries a rare homozygous variant and simultaneously a rare heterozygous/heterozygous variant pair in the same gene.
[e]Only variants in *trans* (located on different copies of the gene) were considered.
[f]One individual had two unphased (unknown whether *cis* or *trans*) heterozygous variants.
[g]One individual had two unphased (unknown whether *cis* or *trans*) heterozygous variants.
[h]Ten individuals had two unphased (unknown whether *cis* or *trans*) heterozygous variants.
[i]Sixteen individuals had two unphased (unknown whether *cis* or *trans*) heterozygous variants.

In the 200,000 exomes release of the UK Biobank, we identified 110 biallelic carriers (47 homozygous and 63 compound heterozygous) and 8,453 monoallelic carriers of pLoF or predicted deleterious missense variants in the *MCM8* gene. Additionally, we found 74 biallelic carriers (15 homozygous and 59 compound heterozygous) and 4,991 monoallelic carriers of pLoF or predicted deleterious missense variants in the *MCM9* gene. Among the 110 biallelic *MCM8* variant carriers in the 200000 UK Biobank, 2 individuals (1.8%) were registered with CRC, 3 (2.7%) with colonic polyps, 4 (3.6%) with adenomas, 1 (0.9%) with female infertility, and 6 (5.5%) with hypothyroidism. Among the 74 biallelic *MCM9* variant carriers in the 200000 UK Biobank, 1 individual (1.4%) was registered with colorectal cancer (CRC), 3 (4%) with colonic polyps, 6 (8%) with adenomas, 1 (1.4%) with rectal polyps, and 2 (2.7%) with hypothyroidism. However, no significant enrichment of biallelic *MCM8/MCM9* pLoF or predicted deleterious missense variants was observed for any of these phenotypes compared to controls in the 200000 UK Biobank (Table 3).

None of the other phenotypes investigated (see Table S1) were registered among the biallelic *MCM8/MCM9* variant carriers, based on ICD-10/ICD-O registrations.

Case series

Phenotype of biallelic germline MCM8/MCM9 *variants carriers*

In our case series, we identified 26 biallelic *MCM8* variant carriers (including 15 with pathogenic or likely pathogenic variants and 11 with a VUS) and 28 biallelic *MCM9* variant carriers (including 22 with pathogenic or likely pathogenic variants and 6 with a VUS) that met the pathogenicity-based filtering criteria. This group included 3 biallelic *MCM8* and 4 biallelic *MCM9* variant carriers who had not been previously described (Figure 1). An overview of all identified *MCM8/MCM9* variant carriers, including their sources, is presented in Table S2. The supplemental information contain a detailed description of all newly identified *MCM8/MCM9* variant carriers and previously documented carriers for whom we obtained updated clinical information (individuals meeting the pathogenicity-based filtering criteria only), with the pedigrees being presented in Figure S1.

Biallelic MCM8/MCM9 *variant carriers often present with hypogonadism linked to impaired gonadal development.* The majority of individuals with biallelic *MCM8* (23 out of 26, 88%) or *MCM9* (26 out of 28, 93%) variants from our case series experienced hypogonadism (HP:0000815) (Figure 2).

Table 2. Enrichment analysis of biallelic *MCM8/MCM9* variants in 100000 Genomes Project, adjusting for age, sex, and ethnicity

Phenotype[a]	Potentially deleterious alleles in cases	Potentially deleterious alleles in controls	Non/unlikely deleterious alleles in cases	Non/unlikely deleterious alleles in controls	OR (95% CI)	*p*
MCM8						
Colonic	14	10	13,198	30,168	1.62 (0.38–6.86)	0.51
CRC	4	10	6,942	30,168	0.52 (0.04–6.08)	0.60
Colonic polyps	6	10	6,096	30,168	1.20 (0.20–7.11)	0.84
Colonic adenomas	6	10	5,748	30,168	2.37 (0.40–14.08)	0.34
Rectal polyps	6	10	2,802	30,168	2.41 (0.38–15.47)	0.35
Hypothyroidism	4	10	6,642	30,168	0.89 (0.14–5.83)	0.91
Breast cancer	10	6[b]	8,866	16,244[b]	1.04 (0.21–5.12)	0.96
Epilepsy	2	10	6,196	30,168	0.77 (0.03–19.72)	0.88
Endometrial cancer	2	6[b]	2,212	16,244[b]	0.83 (0.07–10.31)	0.89
Short stature	2	10	1,986	30,168	0.68 ($3.32x10^{-13}$ to $1.42x10^{12}$)	0.98
Delayed puberty	2	10	256	30,168	1.19 ($1.35x10^{-37}$ to $1.05x10^{37}$)	0.99
MCM9						
Colonic	12	10	13,200	30,168	3.68 (0.74–18.41)	0.11
CRC	6	10	6,940	30,168	1.49 (0.12–18.11)	0.75
Colonic polyps	6	10	6,096	30,168	**6.51 (1.24–34.11)**	**0.03**
Colonic adenomas	4	10	5,750	30,168	1.55 (0.13–18.33)	0.73
Rectal polyps	4	10	2,804	30,168	**8.40 (1.28–55.35)**	**0.03**
Hypothyroidism	6	10	6,640	30,168	3.88 (0.72–20.81)	0.11
Breast cancer	10	2[b]	8,866	16,242[b]	3.53 (0.33–37.2)	0.29
Epilepsy	4	10	6,194	30,168	0.40 (0.02–9.27)	0.57
Endometrial cancer	2	2[b]	2,212	16,242[b]	1.7 (0.01–270.4)	0.83
Melanoma	2	10	1,426	30,168	5.08 (0.43–59.06)	0.19
Gastric cancer	2	10	560	30,168	**27.03 (2.93–248.5)**	**0.004**

Boldface values indicate statistical significance. CI, confidence interval; CRC, colorectal cancer; OR, odds ratio.
[a]No cases with ovarian cancer, cervical cancer, female infertility, primary ovarian insufficiency, male infertility, absent or infantile uterus, or germ cell tumors were identified; as such, these phenotypes are not included in the table. Among the cases, short stature and delayed puberty were reported exclusively in biallelic *MCM8* carriers, while gastric cancer and melanoma were observed only in biallelic *MCM9* carriers.
[b]For the analysis of breast and endometrial cancers, only female controls were included for comparison.

Apart from five males (three with biallelic *MCM8* variants and two with biallelic *MCM9* variants) with azoospermia (no sperm in the semen; HP:0000027), these issues involved women affected by POI. Fourteen out of 20 (70%) individuals affected by POI and carrying biallelic *MCM8* variants had undetectable or small ovaries coupled with an infantile or absent uterus upon ultrasound in 13 (65%) of the affected individuals. Among the biallelic *MCM9* variant carriers affected by POI, 14 out of 23 (61%) exhibited invisible or small ovaries, and 12 out of 23 (52%) had infantile or absent uteri. Furthermore, osteoporosis or delayed bone age (HP:0000939) was reported in seven individuals with biallelic *MCM9* variants and one individual with biallelic *MCM8* variants, all of whom were affected by hypogonadism. In both the *MCM8* and *MCM9* groups, hypogonadism manifested at a relatively young age, typically between 10 and 30 years (Figure 3). Many of these individuals were part of earlier studies, with no updated clinical data available upon request, so most were lost to follow-up post-publication.

Biallelic MCM9 *variant carriers may face polyposis, gastric cancer, and early-onset CRC, while both biallelic* MCM8/MCM9 *carriers may face female germ cell tumors.* Polyposis (typically >20 polyps, including hyperplastic, adenomatous, and serrated types) was reported in 6 out of 28 (21%) biallelic *MCM9* variant carriers from our case series (Figure 2). Similarly, CRC was observed in 6 of 28 (21%) biallelic *MCM9* variant carriers in our case series. This includes three carriers of likely pathogenic variant(s) who developed CRC between the ages of 30 and 40 and three

Table 3. **Enrichment analysis of biallelic *MCM8/MCM9* variants in 200000 UK Biobank, adjusting for age, sex, and ethnicity**

Phenotype[a]	Potentially deleterious alleles in cases	Potentially deleterious alleles in controls	Non/unlikely deleterious alleles in cases	Non/unlikely deleterious alleles in controls	OR (95% CI)	*p*
MCM8						
Colonic	16	136	39,158	181,658	0.54 (0.26–1.14)	0.11
CRC	4	136	6,474	181,658	0.83 (0.20–3.41)	0.80
Colonic polyps	6	136	18,518	181,658	0.43 (0.14–1.39)	0.16
Colonic adenomas	8	136	20,586	181,658	0.52 (0.19–1.44)	0.21
Female infertility	2	74[b]	988	98,908[b]	2.68 (0.34–21.11)	0.35
Hypothyroidism	12	136	21,982	181,658	0.67 (0.29–1.58)	0.36
MCM9						
Colonic	16	84	39,158	181,710	-	-
CRC	2	84	6,476	181,710	0.82 (0.11–5.99)	0.84
Colonic polyps	6	84	18,518	181,710	0.80 (0.25–2.63)	0.72
Colonic adenomas	12	84	20,582	181,710	1.51 (0.63–3.61)	0.35
Rectal polyps	2	84	10,326	181,710	0.49 (0.07–3.59)	0.48
Hypothyroidism	4	84	21,990	181,710	0.46 (0.11–1.94)	0.29

CI, confidence interval; CRC, colorectal cancer; OR, odds ratio.
[a]No cases with breast cancer, gastric cancer, melanoma, endometrial cancer, ovarian cancer, cervical cancer, primary ovarian insufficiency, male infertility, epilepsy, short stature, delayed puberty, absent or infantile uterus, or germ cell tumors were identified; as such, these phenotypes are not included in the table. Among the cases, female infertility was reported exclusively in biallelic *MCM8* carriers, while rectal polyps were observed only in biallelic *MCM9* carriers.
[b]For the analysis of female infertility, only female controls were included for comparison.

carriers with a VUS diagnosed between 40 and 60 years (Figure 3). No CRC or polyp diagnoses were reported among the biallelic *MCM8* variant carriers. Three female carriers—two with biallelic *MCM8* variants and one with a biallelic *MCM9* variant—were diagnosed with germ cell tumors (HP:0100728) between the ages of 11 and 15 years. These included two endodermal sinus tumors originating from dysgerminomas, which themselves arose from gonadoblastomas, and one germ cell tumor of unspecified origin. Single biallelic *MCM9* variant carriers were diagnosed with gastric cancer (HP:0012126), a human papillomavirus-unrelated clear cell carcinoma of the cervix (HP:0031522), and melanoma (HP:0012056), whereas a biallelic *MCM8* variant carrier was diagnosed with breast cancer (HP:0003002).

Monoallelic MCM8/MCM9 *variants may experience hypogonadism.* During the pathogenicity-based filtering process of our case series, we filtered 49 monoallelic *MCM8* variant carriers and 45 monoallelic *MCM9* variant carriers. Out of these 49 monoallelic *MCM8* variant carriers, hypogonadism was noted in 14 (29%) individuals, with two having a likely pathogenic variant and 12 carrying a VUS (Figures S2 and S3). Two monoallelic *MCM8* variant carriers were diagnosed with CRC, another two with polyposis, and two individuals with a monoallelic *MCM8* variant were diagnosed with breast cancer.

Among the 45 monoallelic *MCM9* variant carriers from our case series, 10 (22%) were known to have hypogonadism, including 1 individual who was also diagnosed with CRC and polyposis (Figures S2 and S3). CRC and polyps were additionally reported in 5 and 6 other monoallelic *MCM9* variant carriers, respectively. No other types of cancer were reported in the monoallelic *MCM9* group.

Genotype-phenotype correlations reveal potential hotspot sites

Mapping of variants onto the MCM8 and MCM9 protein domains revealed that the variants in our case series clustered in two key regions: the N-terminal DNA binding domain, which is crucial for protein-DNA binding (6 of 11 *MCM8* variants in biallelic carriers, 55%; 4 of 20 *MCM9* variants in biallelic carriers, 20%), and the AAA+ core domain, essential for DNA helicase activity (5 of 11 *MCM8* variants in biallelic carriers, 45%; 12 of 20 *MCM9* variants in biallelic carriers, 60%) (Figures 4 and S4).[44] Additionally, several variants were found to be shared among multiple families with hypogonadism from our case series. For instance, the c.482A>C [p.(His161Pro)] VUS in the *MCM8* gene, previously linked to hypogonadism,[39,26,46,49,51] was shared by six biallelic carriers across two families. Similarly, the pathogenic c.394C>T [p.(Arg132*)] variant in the *MCM9* gene, also associated with hypogonadism,[39,49,51] was shared by seven biallelic carriers from four unrelated families.

Cancer-specific cohorts

No biallelic *MCM8/MCM9* variant carriers meeting the pathogenicity-based filtering criteria were identified in the SPS case group, fCRCX, and HMF (metastasized CRC and endometrial cancer case groups) cancer-specific

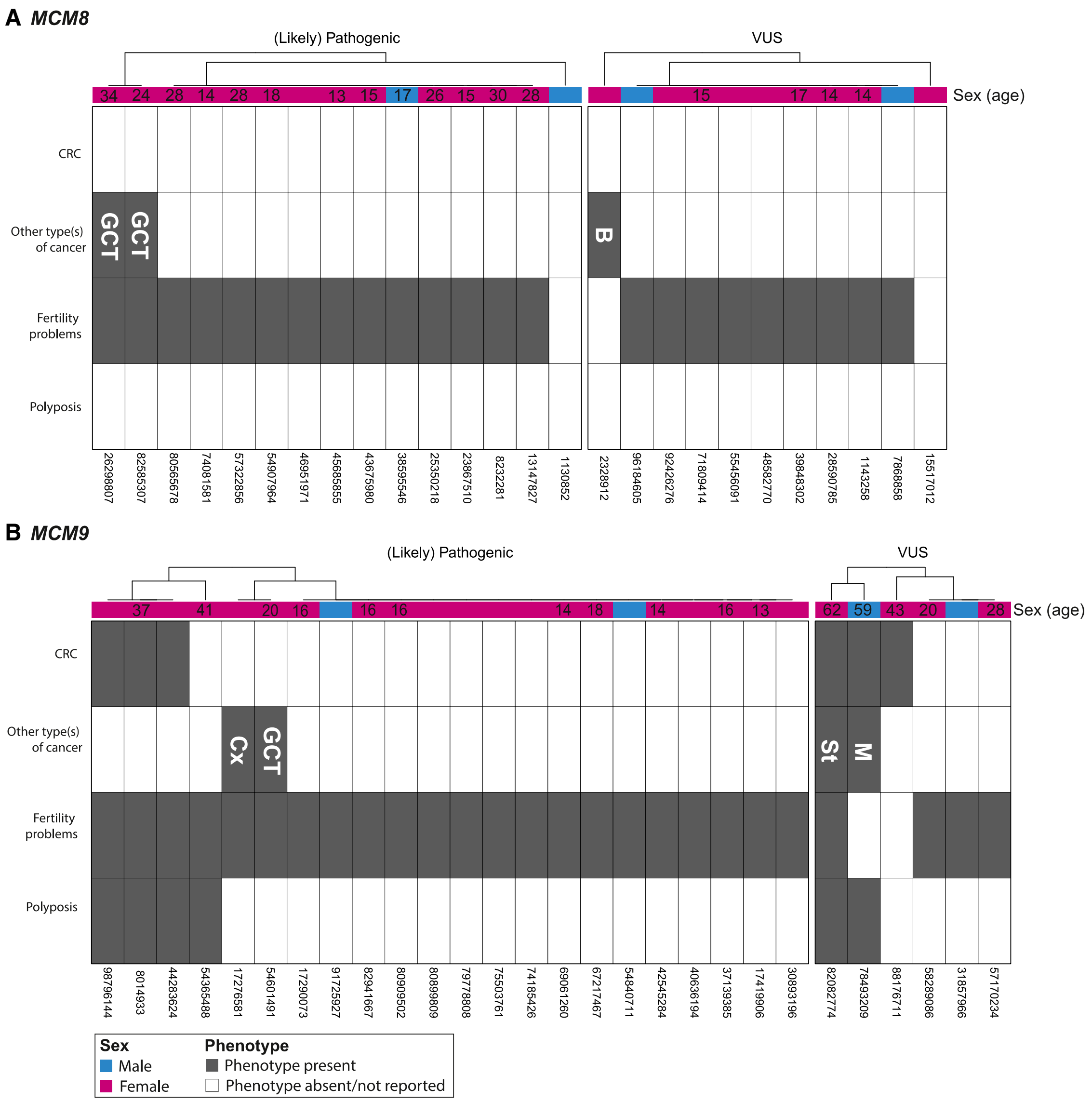

Figure 2. Phenotype of biallelic *MCM8/MCM9* variant carriers
The phenotype is presented for all (A) biallelic *MCM8* and (B) biallelic *MCM9* variant carriers from our case series. Each column represents an individual, while each row corresponds to one of the four primary observed phenotypes: CRC, other type(s) of cancer, hypogonadism, and polyposis. Person IDs are provided below each column, whereas their corresponding ages, which represent the most recent reported age of each individual, are shown above every column (when available). B, breast cancer; Cx, cervical cancer; GCT, germ cell tumor; M, melanoma; St, stomach cancer.

cohorts. In the HMF cancer-specific cohort, four monoallelic *MCM8* and three monoallelic *MCM9* variant carriers meeting the pathogenicity-based filtering criteria were identified with CRC.

Tumor DNA analysis

An overview of the analyzed tumors from the case series is provided in Figure 5A. Germline WES-based DNA analysis, performed using previously described methods,[77] revealed no pathogenic variants in other well-established CRC- or polyposis-associated genes in any of the corresponding participants. Of note, one participant who had three polyps included in the analysis (P6_11T, P6_24A, and P6_24B) carried biallelic VUS in *HROB*, which encodes a protein believed to support the function of *MCM8* and *MCM9*.[9,78,79]

Most tumors in the case series appear diploid; driver mutations were identified in a subset of samples

Copy-number analysis showed that most tumors in the case series were diploid, with no significant gains or losses in CRC-related genes (Figure S5). TMB ranged from less than 1 to 360.42 mutations per megabase, with a median of 27.83 mutations per megabase and an interquartile range of 0.13–27.83. Two tumors—P6_24B and P8_33B—exhibited highly fragmented copy-number profiles, fluctuating between values of 1 and 3. However, these results should be interpreted with caution due to low sequencing depth, which may have limited the ability of CNVkit to

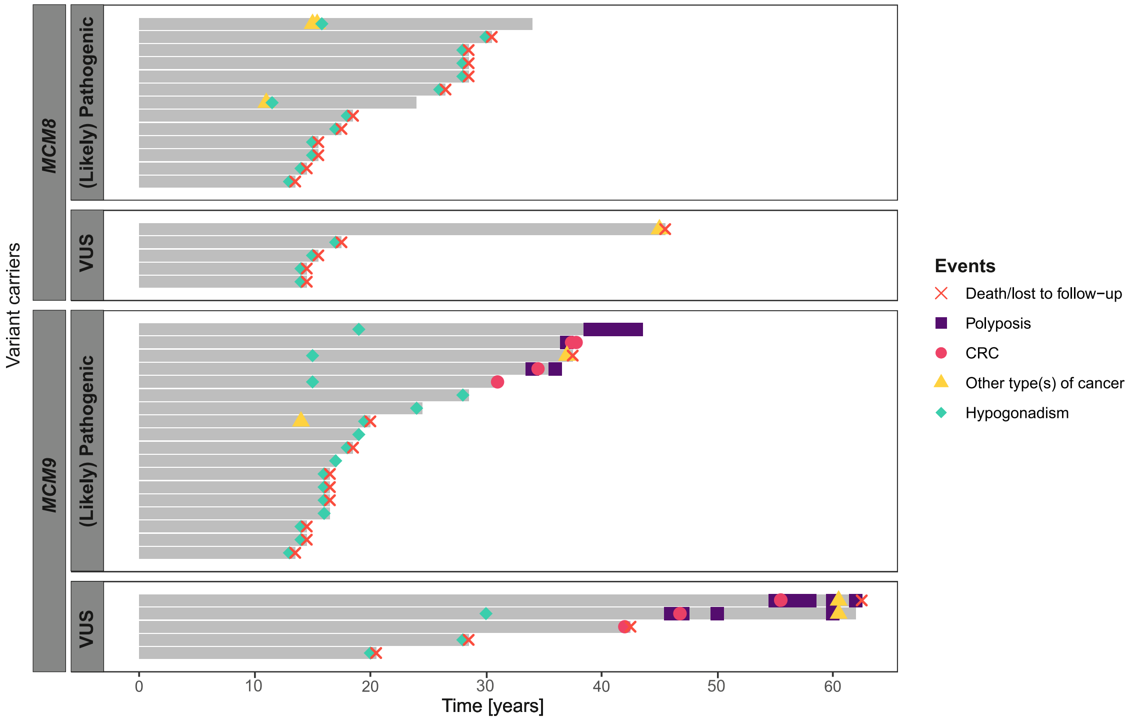

Figure 3. Disease onset in biallelic *MCM8/MCM9* variant carriers
The onset of the four primary observed phenotypes (CRC, other type[s] of cancer, hypogonadism, and polyposis) is displayed for each biallelic *MCM8/MCM9* variant carrier with available age details in our case series. Those without age details were excluded from the analysis. Individuals are ordered by ACMG/AMP classification (pathogenic or likely pathogenic, VUS)[53,54] and current age or age at the time of death/lost to follow-up.

accurately assign copy-number states. Driver mutations in CRC-related genes were detected in a subset of samples (Figure 5B), with detailed information on the specific mutations provided in Table S3.

Clock-like and unknown-etiology signatures dominate tumors from the case series and HMF cancer-specific cohort, while MMR and HR deficiency-associated signatures appear in only a minority of cases

SBS1 and SBS5, which reflect clock-like mutational processes,[80] were detected in all tumors from our case series with matched germline sequencing data, as well as in one CRC and two polyps from a wild-type control (Figure 5C). In addition, SBS1 and SBS5—alongside SBS93 and SBS40, both of unknown origin—were the most prominent signatures in metastasized CRCs from seven individuals with monoallelic *MCM8* or *MCM9* variants in the HMF cancer-specific cohort (Figure S6).

Tumors from our case series lacking matched germline sequencing data were dominated by sequencing artifact signatures (SBS45, SBS47, SBS50, SBS51, SBS54, SBS56, SBS58, and SBS95), limiting our ability to compare these to tumors with matched controls or to previously published cases.[67–71]

Signatures ID1 or ID2, which display a high number of indels in MMR-deficient cases, were detected in 8 of 15 tumors from our case series and in 2 control tumors. SBS26, similarly associated with MMR deficiency, was identified in one tumor (P2_2T), where it contributed to a minority of the mutations (269 out of 2,026, 13%).

Signatures associated with homologous recombination (HR) deficiency, including SBS3 and ID6, were each identified in one tumor (P1_1T and P6_11T, respectively) from two separate participants, both of whom lacked matched germline data. In addition, ID signatures of unknown etiology, including ID4, ID5, ID9, ID10, ID11, ID14, ID15, and ID16, were detected in all but two tumors from our case series and in all three control tumors.

Somatic* MCM8/MCM9 *mutations may occur as a result of other DNA repair deficiencies and mutational processes, potentially involving copy-number variations

In TCGA Pan-Cancer Atlas dataset, insights into the somatic mutational behavior of *MCM8* and *MCM9* were gained through the observation of copy-number alterations in both genes. Furthermore, unsupervised hierarchical clustering of SBS mutational signature profiles revealed clusters characterized by signatures such as SBS7a/b (UV damage), SBS2 and SBS13 (APOBEC activity), SBS6, SBS14, SBS15, SBS20, and SBS21 (MMR deficiency), and SBS10a/b (POLE deficiency),[67–71] which suggest that somatic *MCM8/MCM9* variants may be secondary to other DNA repair deficiencies and mutational processes (Figure S7).

Discussion

Following the initial discovery of biallelic germline *MCM8/MCM9* variants in families with CRC, polyposis,

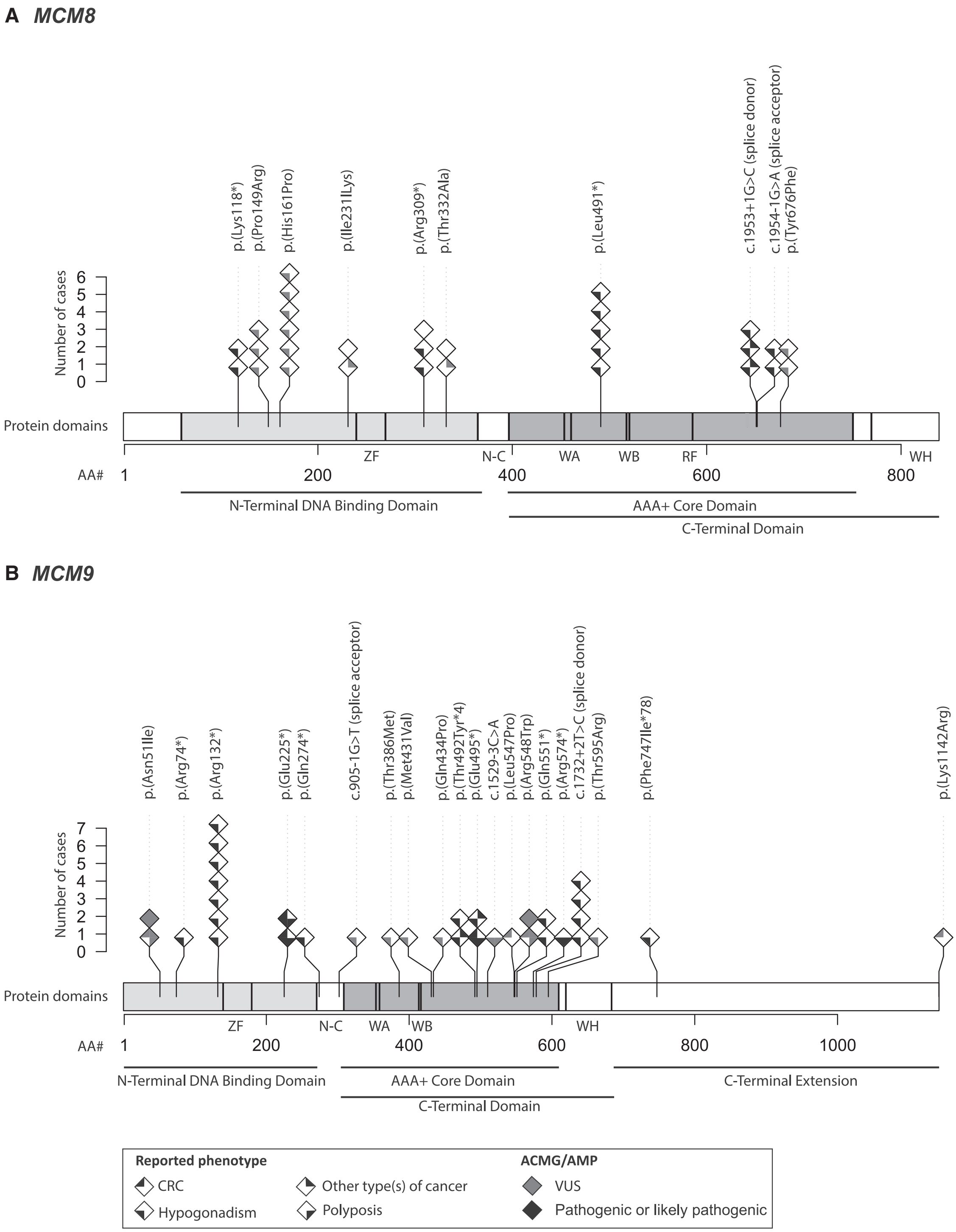

Figure 4. Biallelic *MCM8/MCM9* variants mapped onto the respective protein domains
(A) *MCM8* and (B) *MCM9* variants from all biallelic variant carriers in our case series are mapped onto the domains of the MCM8 and MCM9 proteins, respectively. Each homozygote variant carrier corresponds to one diamond symbol, whereas for compound heterozygous variant carriers, both variants are separately plotted. The fill and color of the diamond symbols correspond to the phenotype of the individual (CRC, other type[s] of cancer, hypogonadism, polyposis) and the ACMG/AMP classification of the variant (pathogenic or likely pathogenic, VUS),[53,54] respectively. N-C, N-C linker domain; RF, arginine finger; VUS, variant of uncertain significance; WA, Walker A; WB, Walker B; WH, winged-helix; ZF, zinc finger.

and hypogonadism,[2–4] we present a comprehensive clinical and molecular characterization of biallelic *MCM8/MCM9* variant carriers from multiple sources. Our analysis of the 100000 Genomes Project reveals that biallelic *MCM9* variant carriers are at increased risk for polyposis and gastric cancer, a pattern not observed in biallelic

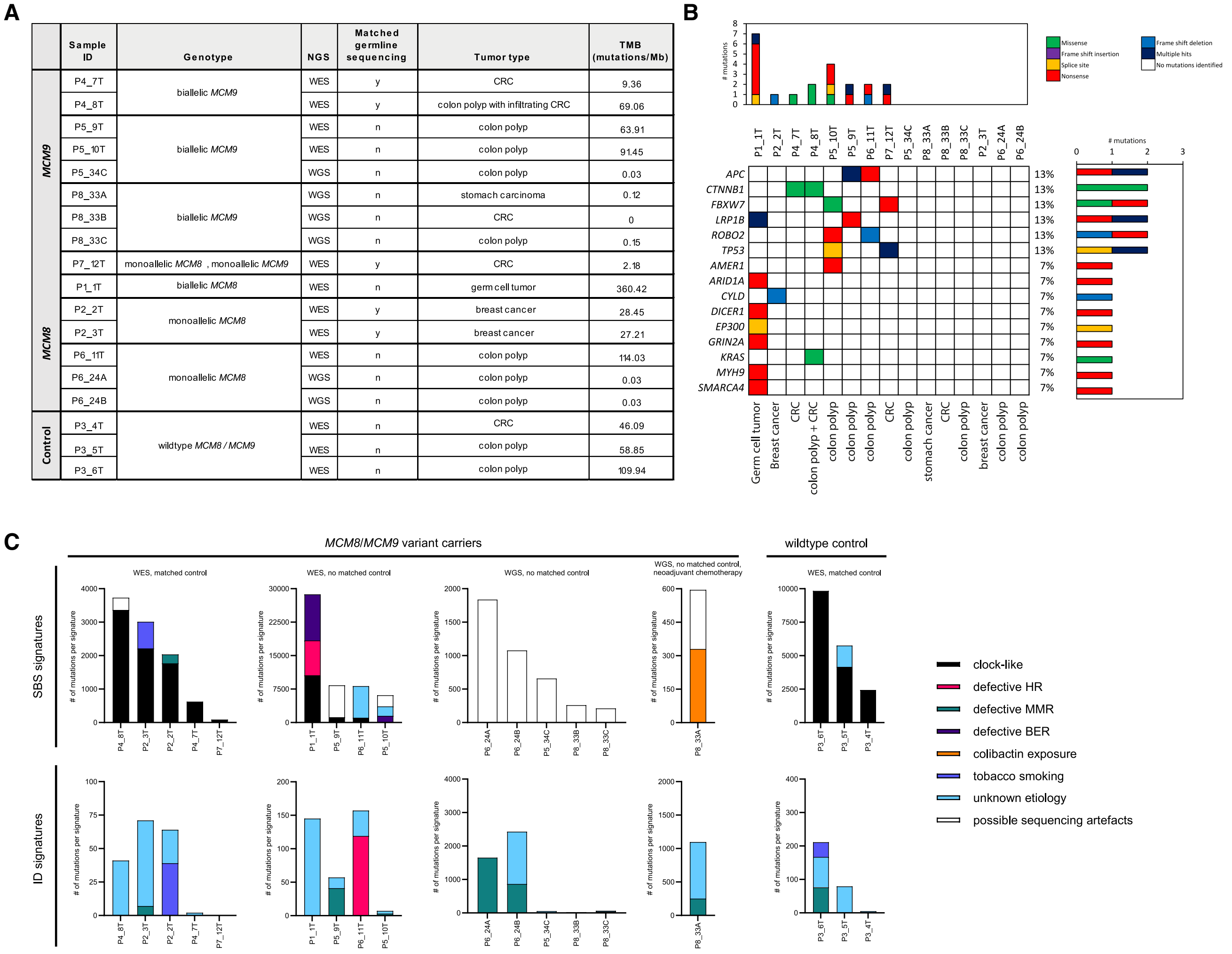

	Sample ID	Genotype	NGS	Matched germline sequencing	Tumor type	TMB (mutations/Mb)
MCM9	P4_7T	biallelic *MCM9*	WES	y	CRC	9.36
	P4_8T		WES	y	colon polyp with infiltrating CRC	69.06
	P5_9T	biallelic *MCM9*	WES	n	colon polyp	63.91
	P5_10T		WES	n	colon polyp	91.45
	P5_34C		WGS	n	colon polyp	0.03
	P8_33A	biallelic *MCM9*	WGS	n	stomach carcinoma	0.12
	P8_33B		WGS	n	CRC	0
	P8_33C		WGS	n	colon polyp	0.15
MCM8	P7_12T	monoallelic *MCM8* , monoallelic *MCM9*	WES	y	CRC	2.18
	P1_1T	biallelic *MCM8*	WES	n	germ cell tumor	360.42
	P2_2T	monoallelic *MCM8*	WES	y	breast cancer	28.45
	P2_3T		WES	y	breast cancer	27.21
	P6_11T	monoallelic *MCM8*	WES	n	colon polyp	114.03
	P6_24A		WGS	n	colon polyp	0.03
	P6_24B		WGS	n	colon polyp	0.03
Control	P3_4T	wildtype *MCM8 / MCM9*	WES	n	CRC	46.09
	P3_5T		WES	n	colon polyp	58.85
	P3_6T		WES	n	colon polyp	109.94

Figure 5. Mutational landscape of tumors from *MCM8/MCM9* variant carriers from our case series
(A) Sample overview of the tumors that were available from our case series for mutational signature analysis, including the corresponding genotype, next-generation sequencing (NGS) approach, the availability of normal control tissue, and the tumor type. Control tissue originated from an individual who tested negative for germline *MCM8/MCM9* variants. TMB was defined as the number of somatic mutations per megabase. For WGS samples, only somatic mutations located within coding exonic regions were included in the TMB calculation, unlike in the mutational signature analyses, where all somatic mutations were considered. Of note, TMB values derived from WGS samples were lower than those from WES samples. This difference may reflect factors such as WGS sample contamination leading to the exclusion of true variants by variant callers, differences in sequencing depth and coverage, or underlying biological differences between the samples. (B) Oncoplot visualizing the detected driver mutations for every tumor. (C) The number of mutations in each signature is presented for every tumor. Mutational signature assignment was performed using SigProfilerAssignment (version 0.0.32)[66] based on the COSMIC version 3.3 single-base substitution (SBS) and insertion and deletion (ID) reference signatures. SBS1 and SBS5 were classified as clock-like mutational signatures. SBS3 and ID6 were considered to be caused by defective homologous repair (HR). SBS26, ID1, and ID2 were linked to defective MMR, and SBS30 and SBS36 were associated with defective base excision repair (BER). SBS88 was attributed to colibactin exposure, and SBS92 and ID3 were attributed to tobacco smoking. SBS37, SBS40, SBS94, ID4, ID5, ID9, ID10, ID11, ID14, ID15, and ID16 were considered to be of unknown etiology, while SBS40, SBS45, SBS50, SBS51, SBS54, SBS56, SBS58, and SBS95 were considered possible sequencing artifacts. ID, insertion and deletion; MMR, mismatch repair; TMB, tumor mutational burden; WES, whole-exome sequencing; WGS, whole-genome sequencing.

MCM8 carriers. This finding is further supported by our case series, which included 26 biallelic *MCM8* and 28 biallelic *MCM9* variant carriers, including 7 previously unreported cases. Furthermore, the case series indicates that in addition to the previously established association with hypogonadism due to impaired gonadal development, biallelic *MCM8* and *MCM9* variants are linked to the development of germ cell tumors, with biallelic *MCM9* variants potentially associated with early-onset CRC. These findings highlight the importance of including *MCM8* and *MCM9* in diagnostic gene panels for relevant clinical contexts and suggest that biallelic carriers may benefit from cancer surveillance.

Gaining an unbiased understanding of the phenotype of biallelic *MCM8/MCM9* variant carriers is currently challenging. This difficulty arises mainly from the limited inclusion of *MCM8/MCM9* genes in current diagnostic gene panels for cancer and polyposis, constraining our

case series, and the relative rarity of germline *MCM8/MCM9* variants in the general population, as reflected by our investigations in gnomAD version 2.1.1, the 100000 Genomes Project, and the 200,000 exome release of the UK Biobank. This rarity may have contributed to the absence of biallelic *MCM8/MCM9* variants in the cancer-specific cohorts and could have influenced the enrichment analysis of these variants in the 100000 Genomes Project and 200000 UK Biobank. Aside from the increased risk of polyposis and gastric cancer associated with biallelic *MCM9* variants in the 100000 Genomes Project, the lack of enrichment for biallelic *MCM8/MCM9* variants in other phenotypes and in the 200000 UK Biobank may be attributed to one of two factors: (1) these variants may not actually contribute to studied phenotypes, or (2) there may be limitations in the analysis itself, such as reliance on the accuracy and consistency of ICD-10/ICD-O registrations and the variant filtering approach, which, partly due to the relative novelty of both genes, relied primarily on *in silico* prediction tools. In regard to our case series, we acknowledge an ascertainment bias, contributing to the high frequency of hypogonadism in our cohort since most individuals examined were from studies primarily focused on fertility problems rather than cancer. In contrast, the occurrence of cancer and polyposis among biallelic *MCM8/MCM9* variant carriers may be underestimated because many individuals in our case series are still young, potentially too young to have developed cancer, and because colonoscopies are not typically recommended for biallelic *MCM8/MCM9* variant carriers. Moreover, the prevalence of the associated phenotypes might be underestimated due to our variant filtering approach, being dependent on limited *in silico* prediction algorithms and data from previous studies, for instance in regard to segregation analysis and variant phasing. This may have led to misclassification of individuals as (biallelic) variant carriers, thereby potentially diluting the observed prevalence of phenotypes in our analyses.

Despite its limitations, our population-based analysis and case series describe the most extensive collection of individuals with biallelic *MCM8/MCM9* variants to date, underscoring the importance of considering these variants in specific clinical contexts. We recommend considering biallelic *MCM9* variants in individuals and families with unexplained polyposis, gastric cancer, germ cell tumors, or (early-onset) CRC, particularly in cases of recessive inheritance and known hypogonadism, until more data are available. Similarly, biallelic *MCM8* variants should be considered in cases of unexplained germ cell tumors, especially when accompanied by recessive inheritance or hypogonadism. Additionally, given previous reports linking biallelic *MCM8* variants to CRC[4] and the potential underestimation of cancer and polyposis in our case series, it may be prudent to consider biallelic *MCM8* variants in cases of unexplained CRC or polyposis until further data are available. As these genes become more integrated into diagnostic gene panels and more families are identified, larger sample sizes and longer follow-up periods will allow for more accurate cancer risk assessments.

Given the range of malignancies observed in our case series, surveillance for these individuals could be considered within a shared decision-making framework, taking into account the current evidence until more data become available. Similar to the *NTHL1*- and *MUTYH*-deficiency syndromes,[81–83] which are associated with CRC and polyposis, the *MCM9*-deficiency syndrome observed in our population-based analysis and case series may warrant comparable surveillance protocols. Established colon surveillance guidelines for *NTHL1*- and *MUTYH*-deficiency syndromes,[81–83] which recommend (bi)annual colonoscopy beginning around 18–20 years of age, could potentially be extended to individuals carrying biallelic *MCM9* variants. However, given the observed onset age of 30–60 years in our series, initiating colonoscopy at 25 years may be more appropriate. Additionally, due to the potential increased risk of gastric cancer, concurrent gastroscopy could be considered. Considering the prevalence of germ cell tumors in female biallelic *MCM8/MCM9* variant carriers, annual ultrasound screening starting at age 10 could be considered, given the early onset of 11–15 years observed in our case series. Further evaluation of cancer risks and the cost-effectiveness of surveillance measures is necessary to develop comprehensive surveillance guidelines.

In contrast to biallelic *MCM8/MCM9* variant carriers, our current data suggest that the phenotype of monoallelic *MCM8*/*MCM9* variant carriers may primarily be limited to hypogonadism, with no clear evidence of an increased cancer risk, which does not seem to justify cancer surveillance for these individuals. Although the prevalence of hypogonadism among monoallelic carriers in our case series (29% for *MCM8*, 22% for *MCM9*) appears higher than the global prevalence (e.g., 3.5% for POI[84]), the potential ascertainment bias in our study, as previously discussed, highlights the need for further research to more fully characterize the phenotype of monoallelic *MCM8/MCM9* variant carriers.

To gain potential causal evidence for a role of *MCM8/MCM9* deficiency in the development of polyps and cancer, future studies exploring the mutational landscape of tumors from *MCM8/MCM9* variant carriers are essential. In the mutational signature analysis from our case series, we observed that clock-like mutational signatures SBS1 and SBS5 dominate in tumors from *MCM8/MCM9* variant carriers with matched germline sequencing data available. However, these clock-like mutational processes, commonly found in most CRCs without specific DNA repair defects and in many other cancer types,[67–71] were not more prevalent in tumors from *MCM8/MCM9* variant carriers than in those from our wild-type control. Mutational signatures associated with HR and MMR deficiency, both linked to MCM8 and MCM9 dysfunction,[4,14–21] were observed in only a minority of tumors, predominantly those lacking matched germline sequencing data. In contrast, ID signatures of

unknown etiology were present in nearly all tumors from our case series. Further studies are therefore needed to determine whether tumors from *MCM8/MCM9* variant carriers are molecularly similar to sporadic cases, or whether additional, unrecognized mutational signatures may be associated with *MCM8/MCM9* deficiency.

In conclusion, our study offers a detailed clinical and molecular characterization of biallelic *MCM8/MCM9* variant carriers from various sources. Our data suggest that biallelic *MCM9* variants are associated with polyposis, gastric cancer, and early-onset CRC, while both biallelic *MCM8* and *MCM9* variants are linked to hypogonadism and the early development of germ cell tumors. These findings support the inclusion of *MCM8/MCM9* in diagnostic gene panels for specific clinical contexts and indicate that carriers might benefit from cancer surveillance. Further studies are essential to accurately assess cancer risk and determine the causative role of *MCM8/MCM9* deficiency in cancer predisposition.

Data and code availability

Original/source data for the population-based analyses presented in the paper are available from the following public repositories: https://gnomad.broadinstitute.org/, https://www.ukbiobank.ac.uk/, and https://www.genomicsengland.co.uk/. Original/source data for the bioinformatic analyses of publicly available WGS datasets is accessible via https://www.hartwigmedicalfoundation.nl/ and https://www.cbioportal.org/.

The datasets supporting the analysis of the case series and cancer-specific cohorts in this study have not been deposited in a public repository due to restrictions from our IRB. However, they are available from the corresponding author upon reasonable request and subject to a data transfer agreement.

Acknowledgments

Please see the supplemental information.

Author contributions

N.C.H.: conceptualization, methodology, formal analysis, investigation, writing – original draft, and visualization. D.C.: formal analysis and writing – review & editing. M.C.J.J., I.v.d.B., T.F.E., A.G., F.J.H., M.M.v.d.H.-E., A.V.D.K., S.K., R.P.K., I.M.M.L., L.E. E.L.O.L., L.H.J.L., M.S.O., J.S., Y.T.-R., C.M.T., F.T., R.M.d.V., D. W., and M.J.W.: investigation and writing – review & editing. C.P., D.T., H.M., R.H.P.V., A.R., M.G., M.A., L.B., M.T., and L.V.: formal analysis, investigation, and writing – review & editing. T.Y., M.D.G., L.B.A., H.M., and T.v.W.: methodology, formal analysis, investigation, and writing – review & editing. S.C.-B. and Y.G.: conceptualization, methodology, formal analysis, investigation, and writing – review & editing. M.N.: conceptualization, methodology, formal analysis, investigation, writing – review & editing, and supervision.

Declaration of interests

The authors declare no competing interests.

Supplemental information

Supplemental information can be found online at https://doi.org/10.1016/j.xhgg.2025.100480.

Web resources

Castor: https://castoredc.com/
cBioPortal: https://www.cbioportal.org/
gnomAD: https://gnomad.broadinstitute.org/
gnomAD Browser Variant Co-occurrence: https://gnomad.broadinstitute.org/variant-cooccurrence?dataset=gnomad_r2_1
Hartwig Medical Foundation: https://www.hartwigmedicalfoundation.nl/
Online Mendelian Inheritance in Man: http://www.omim.org/
Picard: http://broadinstitute.github.io/picard
VariantValidator: https://www.variantvalidator.org/
UK Biobank: https://www.ukbiobank.ac.uk/

Received: December 16, 2024
Accepted: July 14, 2025

References

1. You, Y.N., Borras, E., Chang, K., Price, B.A., Mork, M., Chang, G.J., Rodriguez-Bigas, M.A., Bednarski, B.K., Meric-Bernstam, F., and Vilar, E. (2019). Detection of Pathogenic Germline Variants Among Patients With Advanced Colorectal Cancer Undergoing Tumor Genomic Profiling for Precision Medicine. Dis. Colon Rectum *62*, 429–437.
2. Goldberg, Y., Aleme, O., Peled-Perets, L., Castellvi-Bel, S., Nielsen, M., and Shalev, S.A. (2021). MCM9 is associated with germline predisposition to early-onset cancer-clinical evidence. NPJ Genom. Med. *6*, 78.
3. Goldberg, Y., Halpern, N., Hubert, A., Adler, S.N., Cohen, S., Plesser-Duvdevani, M., Pappo, O., Shaag, A., and Meiner, V. (2015). Mutated MCM9 is associated with predisposition to hereditary mixed polyposis and colorectal cancer in addition to primary ovarian failure. Cancer Genet. *208*, 621–624.
4. Golubicki, M., Bonjoch, L., Acuna-Ochoa, J.G., Díaz-Gay, M., Muñoz, J., Cuatrecasas, M., Ocaña, T., Iseas, S., Mendez, G., Cisterna, D., et al. (2020). Germline biallelic Mcm8 variants are associated with early-onset Lynch-like syndrome. JCI Insight *5*, e140698.
5. Lutzmann, M., and Méchali, M. (2008). MCM9 binds Cdt1 and is required for the assembly of prereplication complexes. Mol. Cell *31*, 190–200.
6. Lutzmann, M., and Méchali, M. (2009). How to load a replicative helicase onto chromatin: a more and more complex matter during evolution. Cell Cycle *8*, 1309–1313.
7. Hartford, S.A., Luo, Y., Southard, T.L., Min, I.M., Lis, J.T., and Schimenti, J.C. (2011). Minichromosome maintenance helicase paralog MCM9 is dispensible for DNA replication but functions in germ-line stem cells and tumor suppression. Proc. Natl. Acad. Sci. USA *108*, 17702–17707.
8. Volkening, M., and Hoffmann, I. (2005). Involvement of human MCM8 in prereplication complex assembly by recruiting hcdc6 to chromatin. Mol. Cell Biol. *25*, 1560–1568.
9. Acharya, A., Bret, H., Huang, J.W., Mütze, M., Göse, M., Kissling, V.M., Seidel, R., Ciccia, A., Guérois, R., and Cejka, P. (2024). Mechanism of DNA unwinding by MCM8-9 in complex with HROB. Nat. Commun. *15*, 3584.

10. Blanton, H.L., Radford, S.J., McMahan, S., Kearney, H.M., Ibrahim, J.G., and Sekelsky, J. (2005). REC, Drosophila MCM8, drives formation of meiotic crossovers. PLoS Genet. *1*, e40.
11. Crismani, W., Portemer, V., Froger, N., Chelysheva, L., Horlow, C., Vrielynck, N., and Mercier, R. (2013). MCM8 is required for a pathway of meiotic double-strand break repair independent of DMC1 in Arabidopsis thaliana. PLoS Genet. *9*, e1003165.
12. Kohl, K.P., Jones, C.D., and Sekelsky, J. (2012). Evolution of an MCM complex in flies that promotes meiotic crossovers by blocking BLM helicase. Science *338*, 1363–1365.
13. Hartmann, M., Kohl, K.P., Sekelsky, J., and Hatkevich, T. (2019). Meiotic MCM Proteins Promote and Inhibit Crossovers During Meiotic Recombination. Genetics *212*, 461–468.
14. Lee, K.Y., Im, J.S., Shibata, E., Park, J., Handa, N., Kowalczykowski, S.C., and Dutta, A. (2015). MCM8-9 complex promotes resection of double-strand break ends by MRE11-RAD50-NBS1 complex. Nat. Commun. *6*, 7744.
15. Lutzmann, M., Grey, C., Traver, S., Ganier, O., Maya-Mendoza, A., Ranisavljevic, N., Bernex, F., Nishiyama, A., Montel, N., Gavois, E., et al. (2012). MCM8- and MCM9-deficient mice reveal gametogenesis defects and genome instability due to impaired homologous recombination. Mol. Cell *47*, 523–534.
16. Natsume, T., Nishimura, K., Minocherhomji, S., Bhowmick, R., Hickson, I.D., and Kanemaki, M.T. (2017). Acute inactivation of the replicative helicase in human cells triggers MCM8-9-dependent DNA synthesis. Genes Dev. *31*, 816–829.
17. Nishimura, K., Ishiai, M., Horikawa, K., Fukagawa, T., Takata, M., Takisawa, H., and Kanemaki, M.T. (2012). Mcm8 and Mcm9 form a complex that functions in homologous recombination repair induced by DNA interstrand crosslinks. Mol. Cell *47*, 511–522.
18. Park, J., Long, D.T., Lee, K.Y., Abbas, T., Shibata, E., Negishi, M., Luo, Y., Schimenti, J.C., Gambus, A., Walter, J.C., and Dutta, A. (2013). The MCM8-MCM9 complex promotes RAD51 recruitment at DNA damage sites to facilitate homologous recombination. Mol. Cell Biol. *33*, 1632–1644.
19. McKinzey, D.R., Gomathinayagam, S., Griffin, W.C., Klinzing, K.N., Jeffries, E.P., Rajkovic, A., and Trakselis, M.A. (2021). Motifs of the C-terminal domain of MCM9 direct localization to sites of mitomycin-C damage for RAD51 recruitment. J. Biol. Chem. *296*, 100355.
20. Traver, S., Coulombe, P., Peiffer, I., Hutchins, J.R.A., Kitzmann, M., Latreille, D., and Méchali, M. (2015). MCM9 Is Required for Mammalian DNA Mismatch Repair. Mol. Cell *59*, 831–839.
21. Liu, K., Wang, Y., Zhu, Q., Li, P., Chen, J., Tang, Z., Shen, Y., Cheng, X., Lu, L.Y., and Liu, Y. (2020). Aberrantly expressed HORMAD1 disrupts nuclear localization of MCM8-MCM9 complex and compromises DNA mismatch repair in cancer cells. Cell Death Dis. *11*, 519.
22. AlAsiri, S., Basit, S., Wood-Trageser, M.A., Yatsenko, S.A., Jeffries, E.P., Surti, U., Ketterer, D.M., Afzal, S., Ramzan, K., Faiyaz-Ul Haque, M., et al. (2015). Exome sequencing reveals MCM8 mutation underlies ovarian failure and chromosomal instability. J. Clin. Investig. *125*, 258–262.
23. Tenenbaum-Rakover, Y., Weinberg-Shukron, A., Renbaum, P., Lobel, O., Eideh, H., Gulsuner, S., Dahary, D., Abu-Rayyan, A., Kanaan, M., Levy-Lahad, E., et al. (2015). Minichromosome maintenance complex component 8 (MCM8) gene mutations result in primary gonadal failure. J. Med. Genet. *52*, 391–399.
24. Dou, X., Guo, T., Li, G., Zhou, L., Qin, Y., and Chen, Z.J. (2016). Minichromosome maintenance complex component 8 mutations cause primary ovarian insufficiency. Fertil. Steril. *106*, 1485–1489.e2.
25. Desai, S., Wood-Trageser, M., Matic, J., Chipkin, J., Jiang, H., Bachelot, A., Dulon, J., Sala, C., Barbieri, C., Cocca, M., et al. (2017). MCM8 and MCM9 Nucleotide Variants in Women With Primary Ovarian Insufficiency. J. Clin. Endocrinol. Metab. *102*, 576–582.
26. Bouali, N., Francou, B., Bouligand, J., Imanci, D., Dimassi, S., Tosca, L., Zaouali, M., Mougou, S., Young, J., Saad, A., and Guiochon-Mantel, A. (2017). New MCM8 mutation associated with premature ovarian insufficiency and chromosomal instability in a highly consanguineous Tunisian family. Fertil. Steril. *108*, 694–702.
27. Zhang, Y.X., He, W.B., Xiao, W.J., Meng, L.L., Tan, C., Du, J., Lu, G.X., Lin, G., and Tan, Y.Q. (2020). Novel loss-of-function mutation in MCM8 causes premature ovarian insufficiency. Mol. Genet. Genomic Med. *8*, e1165.
28. Heddar, A., Beckers, D., Fouquet, B., Roland, D., and Misrahi, M. (2020). A Novel Phenotype Combining Primary Ovarian Insufficiency Growth Retardation and Pilomatricomas With MCM8 Mutation. J. Clin. Endocrinol. Metab. *105*, dgaa155.
29. Wang, F., Guo, S., and Li, P. (2020). Two novel mutations in the MCM8 gene shared by two Chinese siblings with primary ovarian insufficiency and short stature. Mol. Genet. Genomic Med. *8*, e1396.
30. Jin, H., Ahn, J., Park, Y., Sim, J., Park, H.S., Ryu, C.S., Kim, N. K., and Kwack, K. (2020). Identification of potential causal variants for premature ovarian failure by whole exome sequencing. BMC Med. Genom. *13*, 159.
31. Tucker, E.J., Bell, K.M., Robevska, G., van den Bergen, J., Ayers, K.L., Listyasari, N., Faradz, S.M., Dulon, J., Bakhshalizadeh, S., Sreenivasan, R., et al. (2022). Meiotic genes in premature ovarian insufficiency: variants in HROB and REC8 as likely genetic causes. Eur. J. Hum. Genet. *30*, 219–228.
32. Alvarez-Mora, M.I., Todeschini, A.L., Caburet, S., Perets, L.P., Mila, M., Younis, J.S., Shalev, S., and Veitia, R.A. (2020). An exome-wide exploration of cases of primary ovarian insufficiency uncovers novel sequence variants and candidate genes. Clin. Genet. *98*, 293–298.
33. Fauchereau, F., Shalev, S., Chervinsky, E., Beck-Fruchter, R., Legois, B., Fellous, M., Caburet, S., and Veitia, R.A. (2016). A non-sense MCM9 mutation in a familial case of primary ovarian insufficiency. Clin. Genet. *89*, 603–607.
34. Franca, M.M., Funari, M.F.A., Lerario, A.M., Santos, M.G., Nishi, M.Y., Domenice, S., Moraes, D.R., Costalonga, E.F., Maciel, G.A., Maciel-Guerra, A.T., et al. (2020). Screening of targeted panel genes in Brazilian patients with primary ovarian insufficiency. PLoS One *15*, e0240795.
35. Guo, T., Zheng, Y., Li, G., Zhao, S., Ma, J., and Qin, Y. (2020). Novel pathogenic mutations in minichromosome maintenance complex component 9 (MCM9) responsible for premature ovarian insufficiency. Fertil. Steril. *113*, 845–852.
36. Liu, H., Wei, X., Sha, Y., Liu, W., Gao, H., Lin, J., Li, Y., Tang, Y., Wang, Y., Wang, Y., and Su, Z. (2020). Whole-exome sequencing in patients with premature ovarian insufficiency: early detection and early intervention. J. Ovarian Res. *13*, 114.

37. Shen, J., Qu, D., Gao, Y., Sun, F., Xie, J., Sun, X., Wang, D., Ma, X., Cui, Y., Liu, J., and Diao, F. (2021). Genetic etiologic analysis in 74 Chinese Han women with idiopathic premature ovarian insufficiency by combined molecular genetic testing. J. Assist. Reprod. Genet. *38*, 965–978.
38. Turkyilmaz, A., Cayir, A., Yarali, O., Kurnaz, E., Kartal Baykan, E., Arslan Ates, E., and Demirbilek, H. (2021). Clinical characteristics and molecular genetic analysis of a cohort with idiopathic congenital hypogonadism. J. Pediatr. Endocrinol. Metab. *34*, 771–780.
39. Wood-Trageser, M.A., Gurbuz, F., Yatsenko, S.A., Jeffries, E.P., Kotan, L.D., Surti, U., Ketterer, D.M., Matic, J., Chipkin, J., Jiang, H., et al. (2014). MCM9 mutations are associated with ovarian failure, short stature, and chromosomal instability. Am. J. Hum. Genet. *95*, 754–762.
40. Yang, X., Touraine, P., Desai, S., Humphreys, G., Jiang, H., Yatsenko, A., and Rajkovic, A. (2019). Gene variants identified by whole-exome sequencing in 33 French women with premature ovarian insufficiency. J. Assist. Reprod. Genet. *36*, 39–45.
41. McLaren, W., Gil, L., Hunt, S.E., Riat, H.S., Ritchie, G.R.S., Thormann, A., Flicek, P., and Cunningham, F. (2016). The Ensembl Variant Effect Predictor. Genome Biol. *17*, 122.
42. Rentzsch, P., Witten, D., Cooper, G.M., Shendure, J., and Kircher, M. (2019). CADD: predicting the deleteriousness of variants throughout the human genome. Nucleic Acids Res. *47*, D886–D894.
43. Gonzalez-Perez, A., and Lopez-Bigas, N. (2011). Improving the assessment of the outcome of nonsynonymous SNVs with a consensus deleteriousness score, Condel. Am. J. Hum. Genet. *88*, 440–449.
44. Helderman, N.C., Terlouw, D., Bonjoch, L., Golubicki, M., Antelo, M., Morreau, H., van Wezel, T., Castellví-Bel, S., Goldberg, Y., and Nielsen, M. (2023). Molecular functions of MCM8 and MCM9 and their associated pathologies. iScience *26*, 106737.
45. Terradas, M., Munoz-Torres, P.M., Belhadj, S., Aiza, G., Navarro, M., Brunet, J., Capellá, G., and Valle, L. (2019). Contribution to colonic polyposis of recently proposed predisposing genes and assessment of the prevalence of NTHL1- and MSH3-associated polyposes. Hum. Mutat. *40*, 1910–1923.
46. Kherraf, Z.E., Cazin, C., Bouker, A., Fourati Ben Mustapha, S., Hennebicq, S., Septier, A., Coutton, C., Raymond, L., Nouchy, M., Thierry-Mieg, N., et al. (2022). Whole-exome sequencing improves the diagnosis and care of men with non-obstructive azoospermia. Am. J. Hum. Genet. *109*, 508–517.
47. Bally, J.F., Zhang, M., Dwosh, E., Sato, C., Rutka, J., Lang, A. E., and Rogaeva, E. (2022). Genomic study of a large family with complex neurological phenotype including hearing loss, imbalance and action tremor. Neurobiol. Aging *113*, 137–142.
48. Chen, S., Wang, G., Zheng, X., Ge, S., Dai, Y., Ping, P., Chen, X., Liu, G., Zhang, J., Yang, Y., et al. (2020). Whole-exome sequencing of a large Chinese azoospermia and severe oligospermia cohort identifies novel infertility causative variants and genes. Hum. Mol. Genet. *29*, 2451–2459.
49. Jolly, A., Bayram, Y., Turan, S., Aycan, Z., Tos, T., Abali, Z.Y., Hacihamdioglu, B., Coban Akdemir, Z.H., Hijazi, H., Bas, S., et al. (2019). Exome Sequencing of a Primary Ovarian Insufficiency Cohort Reveals Common Molecular Etiologies for a Spectrum of Disease. J. Clin. Endocrinol. Metab. *104*, 3049–3067.
50. Soares de Lima, Y., Arnau-Collell, C., Diaz-Gay, M., Bonjoch, L., Franch-Exposito, S., Munoz, J., Moreira, L., Ocana, T., Cuatrecasas, M., Herrera-Pariente, C., et al. (2021). Germline and Somatic Whole-Exome Sequencing Identifies New Candidate Genes Involved in Familial Predisposition to Serrated Polyposis Syndrome. Cancers (Basel) *13*, 929.
51. Potorac, I., Laterre, M., Malaise, O., Nechifor, V., Fasquelle, C., Colleye, O., Detrembleur, N., Verdin, H., Symoens, S., De Baere, E., et al. (2023). The Role of MCM9 in the Etiology of Sertoli Cell-Only Syndrome and Premature Ovarian Insufficiency. J. Clin. Med. *12*, 990.
52. ERN-GENTURIS (2022). Role of *MCM8/9* variants in (colorectal) cancer predisposition. https://www.genturis.eu/l=eng/research/research-projects/mcm8-9-jun-2022.html.
53. Richards, S., Aziz, N., Bale, S., Bick, D., Das, S., Gastier-Foster, J., Grody, W.W., Hegde, M., Lyon, E., Spector, E., et al. (2015). Standards and guidelines for the interpretation of sequence variants: a joint consensus recommendation of the American College of Medical Genetics and Genomics and the Association for Molecular Pathology. Genet. Med. *17*, 405–424.
54. Richards, C.S., Bale, S., Bellissimo, D.B., Das, S., Grody, W.W., Hegde, M.R., Lyon, E., Ward, B.E.; and Molecular Subcommittee of the ACMG Laboratory Quality Assurance Committee (2008). ACMG recommendations for standards for interpretation and reporting of sequence variations: Revisions 2007. Genet. Med. *10*, 294–300.
55. Genoox. Franklin. https://franklin.genoox.com/clinical-db/home.
56. Quintana, I., Terradas, M., Mur, P., Te Paske, I.B.A.W., Peters, S., Spier, I., Steinke-Lange, V., Maestro, C., Torrents, D., Puiggròs, M., et al. (2023). Wnt genes in colonic polyposis predisposition. Genes Dis. *10*, 753–757.
57. Li, H., and Durbin, R. (2009). Fast and accurate short read alignment with Burrows-Wheeler transform. Bioinformatics *25*, 1754–1760.
58. Van der Auwera, G.A., and O'Connor, B.D. (2020). Genomics in the Cloud: Using Docker, GATK, and WDL in Terra, 1st Edition (O'Reilly Media).
59. Benjamin, D., Sato, T., Cibulskis, K., Getz, G., Stewart, C., and Lichtenstein, L. (2019). Calling Somatic SNVs and Indels with Mutect2. Preprint at bioRxiv. https://doi.org/10.1101/861054.
60. Koboldt, D.C., Zhang, Q., Larson, D.E., Shen, D., McLellan, M.D., Lin, L., Miller, C.A., Mardis, E.R., Ding, L., and Wilson, R.K. (2012). VarScan 2: somatic mutation and copy number alteration discovery in cancer by exome sequencing. Genome Res. *22*, 568–576.
61. Fan, Y., Xi, L., Hughes, D.S.T., Zhang, J., Zhang, J., Futreal, P. A., Wheeler, D.A., and Wang, W. (2016). MuSE: accounting for tumor heterogeneity using a sample-specific error model improves sensitivity and specificity in mutation calling from sequencing data. Genome Biol. *17*, 178.
62. Kim, S., Scheffler, K., Halpern, A.L., Bekritsky, M.A., Noh, E., Källberg, M., Chen, X., Kim, Y., Beyter, D., Krusche, P., and Saunders, C.T. (2018). Strelka2: fast and accurate calling of germline and somatic variants. Nat. Methods *15*, 591–594.
63. Bailey, M.H., Tokheim, C., Porta-Pardo, E., Sengupta, S., Bertrand, D., Weerasinghe, A., Colaprico, A., Wendl, M.C., Kim, J., Reardon, B., et al. (2018). Comprehensive Characterization of Cancer Driver Genes and Mutations. Cell *173*, 371–385.e18.
64. Martinez-Jimenez, F., Muinos, F., Sentis, I., Deu-Pons, J., Reyes-Salazar, I., Arnedo-Pac, C., Mularoni, L., Pich, O.,

Bonet, J., Kranas, H., et al. (2020). A compendium of mutational cancer driver genes. Nat. Rev. Cancer *20*, 555–572.
65. Sondka, Z., Bamford, S., Cole, C.G., Ward, S.A., Dunham, I., and Forbes, S.A. (2018). The COSMIC Cancer Gene Census: describing genetic dysfunction across all human cancers. Nat. Rev. Cancer *18*, 696–705.
66. Diaz-Gay, M., Vangara, R., Barnes, M., Wang, X., Islam, S.A., Vermes, I., Duke, S., Narasimman, N.B., Yang, T., Jiang, Z., et al. (2023). Assigning mutational signatures to individual samples and individual somatic mutations with SigProfilerAssignment. Bioinformatics *39*, btad756.
67. Sondka, Z., Dhir, N.B., Carvalho-Silva, D., Jupe, S., Madhumita, McLaren, K., Starkey, M., Ward, S., Wilding, J., Ahmed, M., et al. (2024). COSMIC: a curated database of somatic variants and clinical data for cancer. Nucleic Acids Res. *52*, D1210–D1217.
68. Otlu, B., Díaz-Gay, M., Vermes, I., Bergstrom, E.N., Zhivagui, M., Barnes, M., and Alexandrov, L.B. (2023). Topography of mutational signatures in human cancer. Cell Rep. *42*, 112930.
69. Alexandrov, L.B., Kim, J., Haradhvala, N.J., Huang, M.N., Tian Ng, A.W., Wu, Y., Boot, A., Covington, K.R., Gordenin, D.A., Bergstrom, E.N., et al. (2020). The repertoire of mutational signatures in human cancer. Nature *578*, 94–101.
70. Alexandrov, L.B., Nik-Zainal, S., Wedge, D.C., Aparicio, S.A.J. R., Behjati, S., Biankin, A.V., Bignell, G.R., Bolli, N., Borg, A., Børresen-Dale, A.L., et al. (2013). Signatures of mutational processes in human cancer. Nature *500*, 415–421.
71. Islam, S.M.A., Diaz-Gay, M., Wu, Y., Barnes, M., Vangara, R., Bergstrom, E.N., He, Y., Vella, M., Wang, J., Teague, J.W., et al. (2022). Uncovering novel mutational signatures by *de novo* extraction with SigProfilerExtractor. Cell Genom *2*, 100179.
72. Talevich, E., Shain, A.H., Botton, T., and Bastian, B.C. (2016). CNVkit: Genome-Wide Copy Number Detection and Visualization from Targeted DNA Sequencing. PLoS Comput. Biol. *12*, e1004873.
73. Ng, P.C., and Henikoff, S. (2001). Predicting deleterious amino acid substitutions. Genome Res. *11*, 863–874.
74. Adzhubei, I.A., Schmidt, S., Peshkin, L., Ramensky, V.E., Gerasimova, A., Bork, P., Kondrashov, A.S., and Sunyaev, S.R. (2010). A method and server for predicting damaging missense mutations. Nat. Methods *7*, 248–249.
75. Tate, J.G., Bamford, S., Jubb, H.C., Sondka, Z., Beare, D.M., Bindal, N., Boutselakis, H., Cole, C.G., Creatore, C., Dawson, E., et al. (2019). COSMIC: the Catalogue Of Somatic Mutations In Cancer. Nucleic Acids Res. *47*, D941–D947.
76. Manders, F., Brandsma, A.M., de Kanter, J., Verheul, M., Oka, R., van Roosmalen, M.J., van der Roest, B., van Hoeck, A., Cuppen, E., and van Boxtel, R. (2022). MutationalPatterns: the one stop shop for the analysis of mutational processes. BMC Genom. *23*, 134.
77. Schubert, S.A., Ruano, D., Joruiz, S.M., Stroosma, J., Glavak, N., Montali, A., Pinto, L.M., Rodríguez-Girondo, M., Barge-Schaapveld, D.Q.C.M., Nielsen, M., et al. (2024). Germline variant affecting p53beta isoforms predisposes to familial cancer. Nat. Commun. *15*, 8208.
78. Huang, J.W., Acharya, A., Taglialatela, A., Nambiar, T.S., Cuella-Martin, R., Leuzzi, G., Hayward, S.B., Joseph, S.A., Brunette, G.J., Anand, R., et al. (2020). MCM8IP activates the MCM8-9 helicase to promote DNA synthesis and homologous recombination upon DNA damage. Nat. Commun. *11*, 2948.
79. Hustedt, N., Saito, Y., Zimmermann, M., Álvarez-Quilón, A., Setiaputra, D., Adam, S., McEwan, A., Yuan, J.Y., Olivieri, M., Zhao, Y., et al. (2019). Control of homologous recombination by the HROB-MCM8-MCM9 pathway. Genes Dev. *33*, 1397–1415.
80. Alexandrov, L.B., Jones, P.H., Wedge, D.C., Sale, J.E., Campbell, P.J., Nik-Zainal, S., and Stratton, M.R. (2015). Clock-like mutational processes in human somatic cells. Nat. Genet. *47*, 1402–1407.
81. Belhadj, S., Mur, P., Navarro, M., González, S., Moreno, V., Capellá, G., and Valle, L. (2017). Delineating the Phenotypic Spectrum of the NTHL1-Associated Polyposis. Clin. Gastroenterol. Hepatol. *15*, 461–462.
82. Nielsen, M., Morreau, H., Vasen, H.F.A., and Hes, F.J. (2011). MUTYH-associated polyposis (MAP). Crit. Rev. Oncol. Hematol. *79*, 1–16.
83. Grolleman, J.E., de Voer, R.M., Elsayed, F.A., Nielsen, M., Weren, R.D.A., Palles, C., Ligtenberg, M.J.L., Vos, J.R., Ten Broeke, S.W., de Miranda, N.F.C.C., et al. (2019). Mutational Signature Analysis Reveals NTHL1 Deficiency to Cause a Multi-tumor Phenotype. Cancer Cell *35*, 256–266.e5.
84. Li, M., Zhu, Y., Wei, J., Chen, L., Chen, S., and Lai, D. (2023). The global prevalence of premature ovarian insufficiency: a systematic review and meta-analysis. Climacteric *26*, 95–102.

HGGA, Volume *6*

Supplemental information

Clinical syndromes linked to biallelic germline variants in *MCM8* and *MCM9*

Noah C. Helderman, Ting Yang, Claire Palles, Diantha Terlouw, Hailiang Mei, Ruben H.P. Vorderman, Davy Cats, Marcos Díaz-Gay, Marjolijn C.J. Jongmans, Ashwin Ramdien, Irma van de Beek, Thomas F. Eleveld, Andrew Green, Frederik J. Hes, Marry M. van den Heuvel-Eibrink, Annelore Van Der Kelen, Sabine Kliesch, Roland P. Kuiper, Inge M.M. Lakeman, Lisa E.E.L.O. Lashley, Leendert H.J. Looijenga, Manon S. Oud, Johanna Steingröver, Yardena Tenenbaum-Rakover, Carli M. Tops, Frank Tüttelmann, Richarda M. de Voer, Dineke Westra, Margot J. Wyrwoll, Mariano Golubicki, Marina Antelo, Laia Bonjoch, Mariona Terradas, Laura Valle, Ludmil B. Alexandrov, Hans Morreau, Tom van Wezel, Sergi Castellví-Bel, Yael Goldberg, and Maartje Nielsen

Table of Contents

Acknowledgements 2

Supplemental case reports 3

Supplemental figures 7

Figure S1 7

Figure S2 11

Figure S3 12

Figure S4 13

Figure S5 15

Figure S6 17

Figure S7 18

Supplemental tables 19

Table S1. 19

Table S2. 21

Table S3. 28

Supplemental references 29

Acknowledgements

The authors sincerely thank all individuals and their families for their participation in this study. This research has been conducted using data from UK Biobank (project code 86977), a major biomedical database (www.ukbiobank.ac.uk). Moreover, this research was made possible through access to data in the National Genomic Research Library (project code 1142), which is managed by Genomics England Limited (a wholly owned company of the Department of Health and Social Care). The National Genomic Research Library holds data provided by patients and collected by the NHS as part of their care and data collected as part of their participation in research. The National Genomic Research Library is funded by the National Institute for Health Research and NHS England. The Wellcome Trust, Cancer Research UK and the Medical Research Council have also funded research infrastructure. Moreover, this work is supported (not financially) by the European Reference Network on Genetic Tumour Risk Syndromes (ERN GENTURIS). ERN GENTURIS is funded by the European Union. Finally, this publication and the underlying study have been made possible partly based on data that Hartwig Medical Foundation and the Center of Personalised Cancer Treatment (CPCT) have made available to the study through the Hartwig Medical Database (reference number HMF-DR-288).

MD-G was supported by a fellowship within the "Generación D" initiative, Red.es, Ministerio para la Transformación Digital y de la Función Pública, for talent attraction (C005/24-ED CV1), being by the European Union NextGenerationEU funds, through PRTR. MG and MA were supported by Foundation Nelia et Amadeo Barletta and the Argentinian National Cancer Institute. LB and SCB were supported by Fondo de Investigación Sanitaria/FEDER (20/00113, 23/00189), Fundació La Marató de TV3 (2019-202008-10), Fundación Científica de la Asociación Española contra el Cáncer (PRYGN211085CAST), "la Caixa" Banking Foundation, CERCA Program (Generalitat de Catalunya), and Agència de Gestió d'Ajuts Universitaris i de Recerca (Generalitat de Catalunya, GRPRE 2017SGR21). CIBEREHD and CIBERONC are funded by the Instituto de Salud Carlos III. The work was carried out (in part) at the Esther Koplowitz Centre, Barcelona. MT and LV's research activity is funded by the Spanish Ministry of Science and Innovation (Agencia Estatal de Investigación), co-funded by FEDER funds (PID2020-112595RB-I00). JS was funded by the German Federal Ministry for Education and Research (BMBF) as part of the Junior Scientist Research Centre 'ReproTrackMS' (grant 01GR2303). FT was supported by grants from the German Research Foundation in the frame of the Clinical Research Unit 'Male Germ Cells' (DFG CRU326, project number 329621271). MJW was supported by the DFG Walter Benjamin Programme (award WY 215/1-1). The funders had no role in the study design, data acquisition and analysis, decision to publish, or preparation of the manuscript.

Supplemental case reports

*MCM8*_01

In a Dutch, non-consanguineous family, two daughters (III.1, age 15; III.4, age 11) presented sequentially with streak ovaries (lacking primordial follicles) and a type II germ cell tumor. Both germ cell tumors were endodermal sinus tumors originating from dysgerminomas, which in turn arose from gonadoblastomas. At age 13, III.4 also developed an enchondroma in the left femur. Germline genetic testing revealed a pathogenic c.1953+1G>C splice donor and a 2027A>T [p.(Tyr676Phe)] VUS in the *MCM8* gene, both present in the daughters. Segregation analysis showed the pathogenic splice variant was inherited from the healthy father (II.1), and the missense variant from the mother (II.2). II.2 was diagnosed with a well-differentiated infiltrating ductal carcinoma (ER+/PR+) and ductal carcinoma in situ (grade I) at age 48, and with three primary invasive carcinoma NSTs (2xER+/PR+; 1xER+/PR-) at age 56. II.1 and II.2 also had two healthy daughters (III.2, III.3) and two abortions, one spontaneous at 11 weeks. III.2 and III.3 are heterozygous carriers of the missense and splice variants, respectively.

*MCM8*_02

The index patient (III.7) is a heterozygous carrier of the c.2209G>A [p.(Ala737Thr)] VUS in the *MCM8* gene and the benign c.1865C>T [p.(Ala622Val)] variant in the *BRCA1* gene. She has two children (IV.7, IV.9) and was diagnosed with ovarian cancer at age 58, which was treated with debulking and adjuvant chemotherapy. At age 59, she developed non-Hodgkin lymphoma, and at age 67, she was diagnosed with moderately differentiated colorectal cancer (MSS). The ovarian cancer metastasized when she was 68, and the non-Hodgkin lymphoma metastasized when she was 83. Her father (II.10), paternal half-brother (III.4), and maternal aunt (II.13) were diagnosed with colorectal cancer at ages 66, 63, and 88, respectively. Her paternal grandmother (I.2) and three paternal aunts (II.1, II.2, II.8) were diagnosed with breast cancer at ages 68-75, 95, 57, and 78, respectively.

*MCM8*_03

The index patient (III.6) presented to the clinical genetics department after the detection of 30 polyps (tubular adenomas with low-grade dysplasia) at age 63. Whole exome sequencing (WES) identified a heterozygous c.482A>G [p.(His161Arg)] variant of uncertain significance in the *MCM8* gene, and three heterozygous variants in the *HROB* gene, including the likely pathogenic c.1267C>T [p.(Gln423*)] variant and two missense variants, c.1363C>G [p.(Leu455Val)] and c.1318A>G [p.(Ser440Gly)]. The latter two variants were in cis on one haplotype and classified as variants of uncertain significance according to the American College of Medical Genetics and Genomics guidelines. Notably, the c.1318A>G [p.(Ser440Gly)] variant may introduce a cryptic splice site leading to protein disruption.

Besides polyps, III.6 had a son (IV.4) via a donor egg due to dysfunctional ovaries lacking oocytes. She experienced nocturnal generalized epileptic seizures between ages 42-46, low bone density at age 53, and COPD (GOLD II) at age 55. Her unrelated Dutch parents (II.10, II.11) included her father, who had acute myelocytic leukemia and died at 84, and her mother, who had a tubulovillous adenoma at 70±1 and died at 83. They had seven children, including five daughters (III.2, III.3, III.6, III.10, III.11) and three sons (III.7, III.8, III.9), with the youngest two being fraternal twins.

Genetic testing of two relatives, a brother (III.8) and a maternal uncle (II.21), revealed that both did not have the *MCM8* variant. The brother carried all three *HROB* variants, developed a squamous cell carcinoma on his ear at 49, was diagnosed with over 150 polyps from age 59, was infertile (azoospermia), and experienced partial seizures before age 29, controlled with carbamazepine. The uncle, negative for *MCM8* and *HROB* variants, developed moderately differentiated rectal adenocarcinoma (Dukes C2; MMR proficient) at 59, treated with abdominoperineal rectum amputation and radiotherapy. He also had multiple (>45) adenomatous polyps and six facial basal cell carcinomas at ages 85 and 88.

Other family members, not tested for *MCM8/HROB* variants, were diagnosed with polyps (II.22, III.2, III.9) and/or cancer (I.4, II.12, II.18, II.19, II.22, III.3, III.9).

***MCM8*_04**

Multiple family members (I.4, III.2, III.6, III.7, III.9, III.15, IV.1) were affected by (early-onset) breast cancer. Germline whole exome sequencing analysis of the index patient (III.15), who was diagnosed with breast cancer at 45 and died from unspecified colon pathology, revealed two heterozygous VUS in the *MCM8* gene: c.692T>A [p.(Ile231Lys)] and c.994A>G [p.(Thr332Ala)]. Additionally, a heterozygous pathogenic variant in the *CHEK2* gene, c.1100del [p.(Thr367Met*15)], was identified. Neither the *MCM8* nor *CHEK2* variants fully segregated with the breast cancer phenotype. The *CHEK2* variant was found in a daughter (IV.1) of the index's sister (III.1; obligate *CHEK2* carrier), both of whom had breast cancer at ages 49 and 41, respectively, but was absent in other breast cancer-affected family members (III.6, III.7, III.9). The *MCM8* c.692T>A [p.(Ile231Lys)] variant was present in a maternal uncle (II.5) and four sisters (III.9, III.11, III.13, III.17) of the index, with only one (III.9) affected by breast cancer at age 67. The *MCM8* c.1100del [p.(Thr367Met*15)] variant was identified in one sister of the index (III.11), who, like the index, is a compound heterozygous carrier of both *MCM8* variants but had no reported cancer or other pathologies. Both *MCM8* variants were absent in III.1, III.4, III.6, III.7, IV.2, IV.7, IV.9, and IV.10.

***MCM8*_05**

A 40-year-old male, previously reported by Golubicki et al.[1], was diagnosed with stage IIIB (T4N1M0) left-sided colon cancer. The tumor was a well-differentiated, mucinous adenocarcinoma. The patient reported no family history of cancer but did mention fertility problems, including his spouse experiencing an advanced pregnancy miscarriage. Genetic testing revealed the pathogenic c.351_354del [p.(Lys118Glu*5)] and benign c.414A>G [p.(Ile138Met)] variants in the *MCM8* gene. Previous tumor analysis in this patient showed an MSI-positive (microsatellite instability) phenotype with the loss of MLH1/PMS2 protein expression, while *BRAF* V600E was wildtype, and there was no somatic *MLH1* promotor hypermethylation. Somatic WES indicated a high tumor mutational burden of 77 single nucleotide variants per megabase, with a significant contribution from the MMR deficiency-associated mutational signature SBS15.

***MCM9*_01**

A patient with MMR proficient CRC was found to carry a heterozygous pathogenic c.1987dup [p.(Ser663Phe*36)] variant in the *MCM9* gene. This patient also had a family history of CRC.

***MCM9*_02**

In a Dutch family with multiple cancer and polyposis cases, the index patient (III.1) was found to carry a c.1915C>G [p.(Leu639Val)] VUS in the *MCM9* gene. This patient was diagnosed with MMR proficient adenocarcinoma of the sigmoid colon at 25 years old and metastasized signet ring cell carcinoma at 36, resulting in his death. The patient's father (II.3) and paternal grandfather (I.1) had melanoma and lung cancer, respectively, while his mother (II.4) and all maternal uncles (II.5, II.7) and aunts (II.8, II.10, II.12, II.14) had polyps. One maternal uncle (II.5) also had a diffuse growing adenocarcinoma of the distal esophagus and stomach at 56, and the maternal grandfather (I.4) had primary colorectal carcinomas (MMR proficient) at 47, 60, and 69.

***MCM9*_03**

In a consanguineous Ashkenazi family, previously described by Goldberg et al.[2, 3], two female siblings (III.3, III.4) were initially evaluated for hypergonadotropic hypogonadism and POI. Both were diagnosed with multiple polyps (>20) and CRC at young ages (34 and 37 years, respectively) but responded well to FOLFOX chemotherapy, with no current evidence of disease. Germline genetic analysis revealed a homozygous c.672_673delinsC [p.(Glu225*)] variant in the *MCM9* gene, considered likely pathogenic. The siblings' parents (I.3, II.1), their two other female siblings (III.1, III.2), and their maternal grandmother/paternal aunt (I.2) were heterozygous carriers of this variant. The mother (II.1) was diagnosed with over ten polyps between the ages of 53-65, and the father (II.2) was diagnosed with CRC and a polyp at age 83. The other siblings (III.1, III.2) did not have CRC, though III.2 had two polyps detected at age 39.

***MCM9*_04**

In a consanguineous family of Middle Eastern Arabic origin, previously described by Goldberg et al.[3], multiple carriers of the pathogenic c.1483G>T [p.(Glu495*)] variant in the *MCM9* gene were identified. The related parents (II.2, II.3) were both heterozygous carriers of this variant and each had three polyps diagnosed between the ages of 66-68 years. They had three daughters (III.1, III.2, III.4) and two sons (III.3, III.6). Two daughters (III.1, III.2), who were homozygous carriers of the c.1483G>T [p.(E495*)] variant, were diagnosed with POI around age 15. One of these daughters (III.1) was also diagnosed with CRC at age 31, while the other (III.2) was diagnosed with clear cell carcinoma of the cervix at age 37. One son (III.3), a heterozygous carrier of the variant, was diagnosed with severe oligozoospermia and microsatellite stable (MSS) CRC at age 35. The third daughter (III.4), also a heterozygous carrier, had four children.

***MCM9*_05**

This family was previously described by Potorac et al.[4] Two sisters (IV.1, age 24; IV.6, age 17) from consanguineous Syrian parents (II.13, II.14) were diagnosed with POI, characterized by absent ovaries and no or hypotrophic uteri. Genetic analysis revealed a homozygous pathogenic c.394C>T [p.(Arg132*)] variant in the *MCM9* gene in both sisters. Another sister (IV.2) and a brother (IV.3), who later presented with POI and non-obstructive azoospermia, respectively, were also homozygous carriers of this variant. A second brother (IV.7), a heterozygous carrier, had normal puberty. Additionally, III.13, III.14, IV.1, IV.3, and IV.6 all suffered from severe osteoporosis. Genetic panel analysis for osteoporosis in IV.3 revealed a heterozygous c.106G>A [p.(Gly36Arg)] variant in the *COL1A2* gene, although this variant did not fully segregate with the bone pathology (III.13 was wildtype, while III.14 and IV.6 were heterozygous carriers).

***MCM9*_06**

The index patient (III.1) was diagnosed with POI at 19 years old, along with her twin sisters (III.4, III.5), who never experienced puberty and exhibited streak ovaries and a small uterus. Their father (II.3) is Irish, and their unrelated mother (II.4) is Hungarian, with no reported family history of cancer and normal colonoscopy results for both parents. The index patient's brothers (III.2, III.3) had normal puberty and health, although they have not had children. During her first pregnancy at age 39 (using a donor egg), the index patient (III.1) developed severe diarrhea, leading to the discovery of a likely pathogenic c.1720C>T [p.(Arg574*)] variant and a c.1529-3C>A VUS in the *MCM9* gene through germline genetic analysis. Segregation analysis revealed that the father (II.3) carries the heterozygous c.1720C>T [p.(Arg574*)] variant, while the mother (II.4) carries the heterozygous c.1529-3C>A variant. Since identifying the biallelic *MCM9* variants, the index patient (III.1) has undergone colonoscopies every six months, with 1-2 polyps removed per procedure. The siblings of the index patient did not undergo genetic testing or colonoscopies.

***MCM9*_07**

At 16 years old, the index patient (IV.2) from a Belgian family presented with primary amenorrhea and delayed puberty. Genetic testing revealed compound heterozygous variants in the *MCM9* gene: c.820C>T [p.(Gln274*)] and c.2237_2238dup [p.(Phe47I78)], both classified as likely pathogenic, along with the likely pathogenic c.1222G>T [p.(Asp408Tyr)] variant in the *FSHR* gene. Segregation analysis showed that the father (III.6), who also experienced delayed puberty, carried the heterozygous c.2237_2238dup [p.(Phe47I78)] variant, while the mother (III.7) carried the heterozygous c.820C>T [p.(Gln274*)] variant. The index patient's parents were unrelated.

***MCM9*_08**

The index patient (III.8), a mother of two daughters, carried the compound heterozygous variants c.3425A>G [p.(Lys1142Arg)] and c.1640T>C [p.(Leu547Pro)] in the *MCM9* gene, both classified as VUS per ACMG/AMP guidelines.[5, 6] At 42 years old, III.8 was diagnosed with metastatic poorly differentiated colorectal cancer (MMR proficient), which initially metastasized to the lymph nodes. Despite treatment including rectum resection, chemotherapy (5-fluorouracil/leucovorin), and

radiotherapy, the cancer spread to the liver, leading to her death at 43 years old. The index's brother died from metastatic pancreatic cancer at age 48, while a maternal aunt (II.8) succumbed to leukemia at age 73, and a paternal aunt (II.2) had myoepithelial adenocarcinoma of the left parotid gland at age 67, two primary colorectal cancers (at age 71 and 77), and lobular carcinoma of the right breast at age 76.

***MCM9*_09**

After experiencing a miscarriage (IV.7), the index patient (III.9) was diagnosed with POI before the age of 40. She and her partner (III.8), who is in good health, adopted two daughters. At age 46, III.9 was diagnosed with moderately differentiated colorectal cancer (microsatellite instability, MSH2-/MSH6-), and multiple colon polyps were subsequently found. By age 61, she developed poorly differentiated stomach adenocarcinoma (MMR proficient, microsatellite stable), treated with fluorouracil, oxaliplatin, and docetaxel (FLOT). WES revealed heterozygous variants c.1642C>T [p.(Arg548Trp)] and c.152A>T [p.(Asn51Ile)] in the *MCM9* gene, both classified as VUS. These variants were also detected in III.7, her brother, who developed well-differentiated colorectal cancer (MMR proficient, microsatellite stable) at 55, alongside over 70 colon polyps and melanoma at 60. III.7's son (IV.4) was born with an open abdomen, dextrocardia, and pulmonary agenesis. Another brother (III.2) of III.9, survived by his two children (IV.1, IV.2), died from small cell lung cancer at 59.

***MCM9*_10 (M2013)**

The index patient (III.1) from a consanguineous Syrian family consulted for infertility and was diagnosed with azoospermia at 28 years of age. Because of his small testicular volumes (7/8 mL, left/right, reference >12mL per testis) and significantly increased serum FSH (27.9 U/L, reference range 1-7 U/L), he most likely had spermatogenic failure. Indeed, an externally performed testicular punch biopsy showed spermatocytes as most advanced germ cells indicating an arrest at meiosis confirming that the infertility is due to non-obstructive azoospermia (NOA). Exome sequencing within the Male Reproductive Genomics (MERGE) study revealed that he carried the homozygous pathogenic loss of function variant c.394C>T [p.(Arg132*)] in the *MCM9* gene. The case/variants have been reported before in Wyrwoll et al.[7] No other family members underwent testing for this variant because they did not consent to be included in the study. His sister (III.7) was diagnosed with colorectal cancer at 35 years old, and another sister (III.10) was diagnosed with POI. One of his brothers (III.9) had congenital intellectual disability (microcephaly), and two cousins (III.12, III.13) passed away due to consequences of mental retardation.

***MCM8*_*MCM9*_01**

The index patient (III.2) carried a heterozygous c.832C>T, [p.(Arg278Cys)] VUS in the *MCM8* gene and a heterozygous c.3425A>G, [p.(Lys1142Arg)] VUS in the *MCM9* gene. She was diagnosed with a moderately differentiated colorectal cancer (RER-) at 52 years old. Despite undergoing left-sided hemicolectomy and treatment with 5-fluorouracil/levamisole, the cancer metastasized to the distal ileum, pelvis minor, and an ovary by age 54, leading to her death at 56. The index patient, who had two sons (IV.1, IV.2), also suffered from ovarian cysts and was diagnosed with an adenomatous polyp and a hyperplastic polyp in the colon at 53 years old. Her brother (III.2), father (II.3), and paternal uncle (II.1) were all diagnosed with colorectal cancer. The father (II.3) also developed stomach cancer at 79 years old, while the paternal grandfather (I.1) of the index had a history of either colorectal or stomach cancer (unclear based on medical records).

Supplemental figures

Figure S1

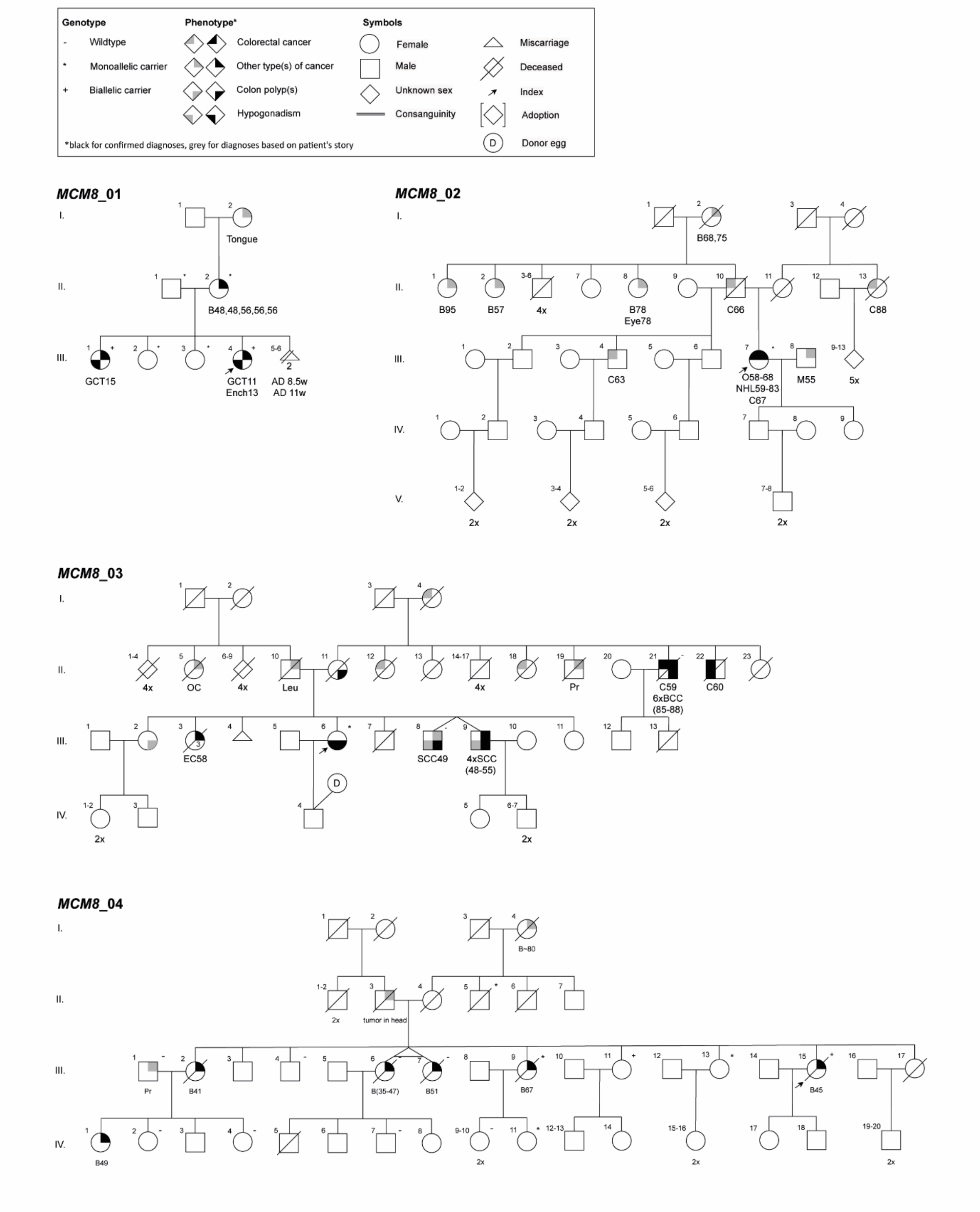

Figure S1 [continued]

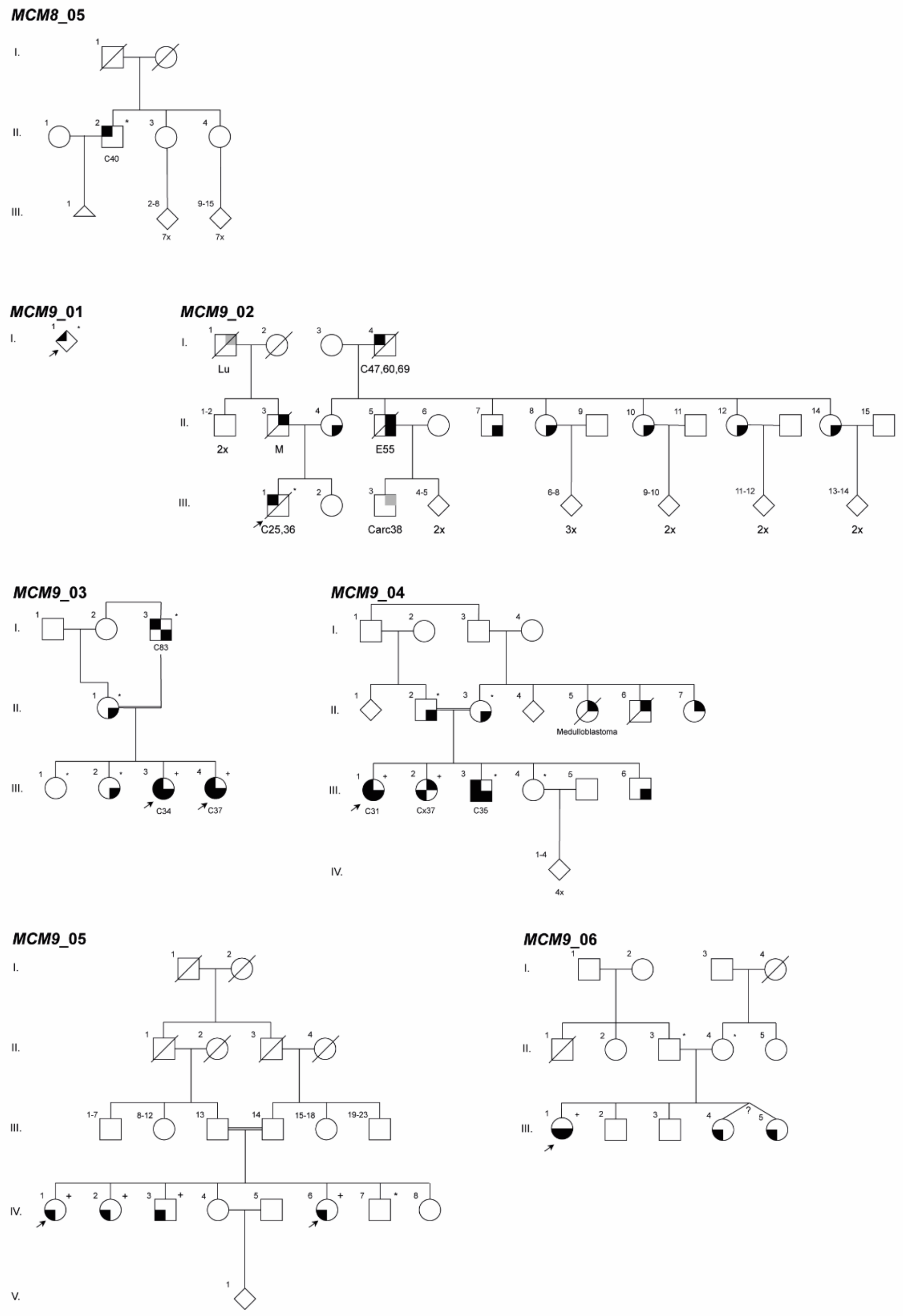

Figure S1 [continued]

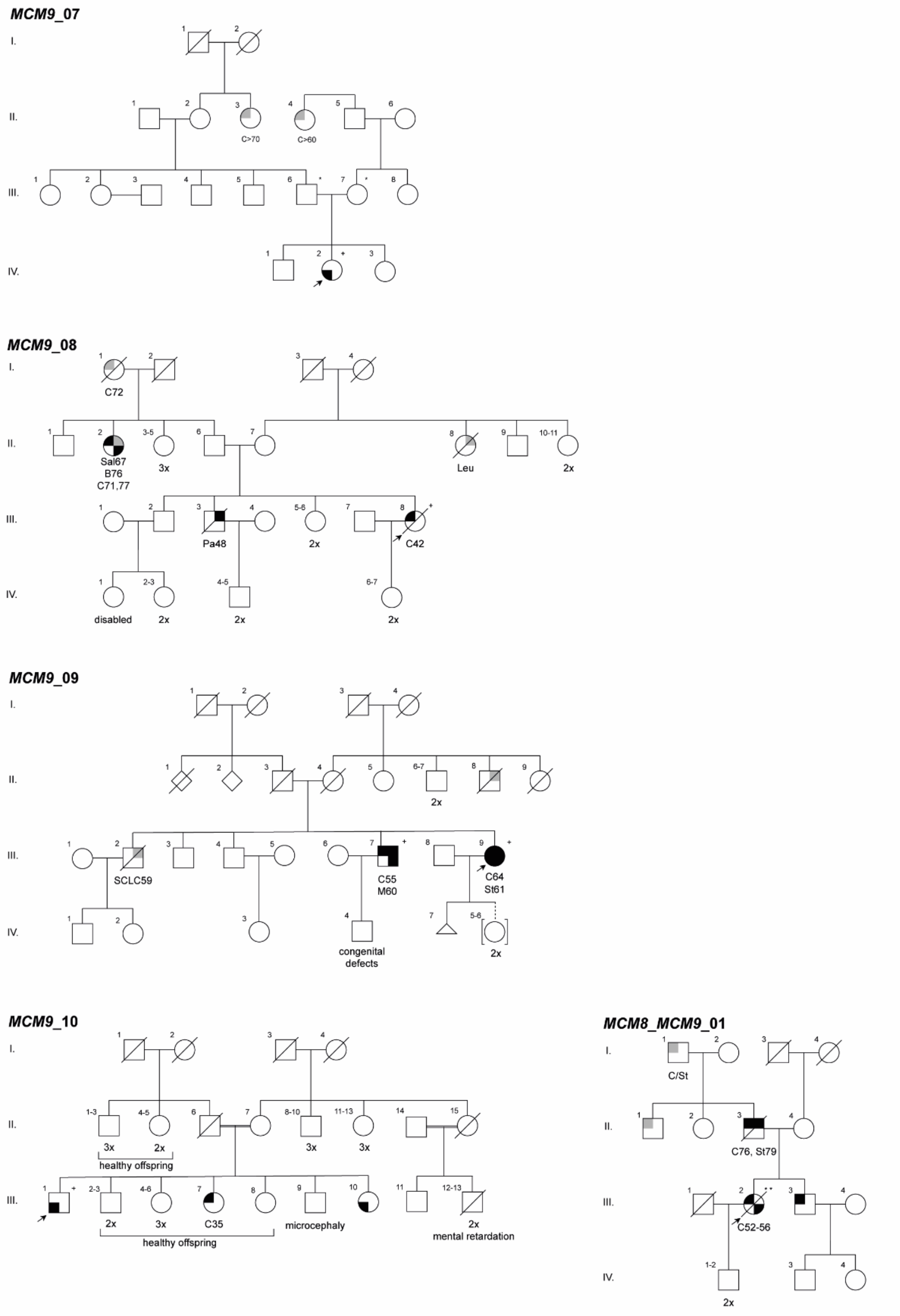

Figure S1. Pedigrees of all newly identified *MCM8/MCM9* variant carriers and previously documented carriers for whom we obtained updated clinical information. Only pedigrees of families

with variants meeting the pathogenicity-based filtering criteria are shown. *AD, amenorrhoea duration; B, breast cancer; BCC, basal cell carcinoma; C, colorectal cancer; Cx, cervical cancer; E, esophagus cancer; EC, endometrial cancer; Ench, enchondroma; GCT, germ cell tumor; Leu, leukemia; Lu, lung cancer; M, melanoma; NHL, non-Hodgkin lymphoma; O, ovarian cancer; Pa, pancreatic cancer; Pr, prostate cancer; Sal, salivary gland cancer; SC C, squamous cell carcinoma; SCL, small-cell lung cancer; St, stomach cancer.*

Figure S2

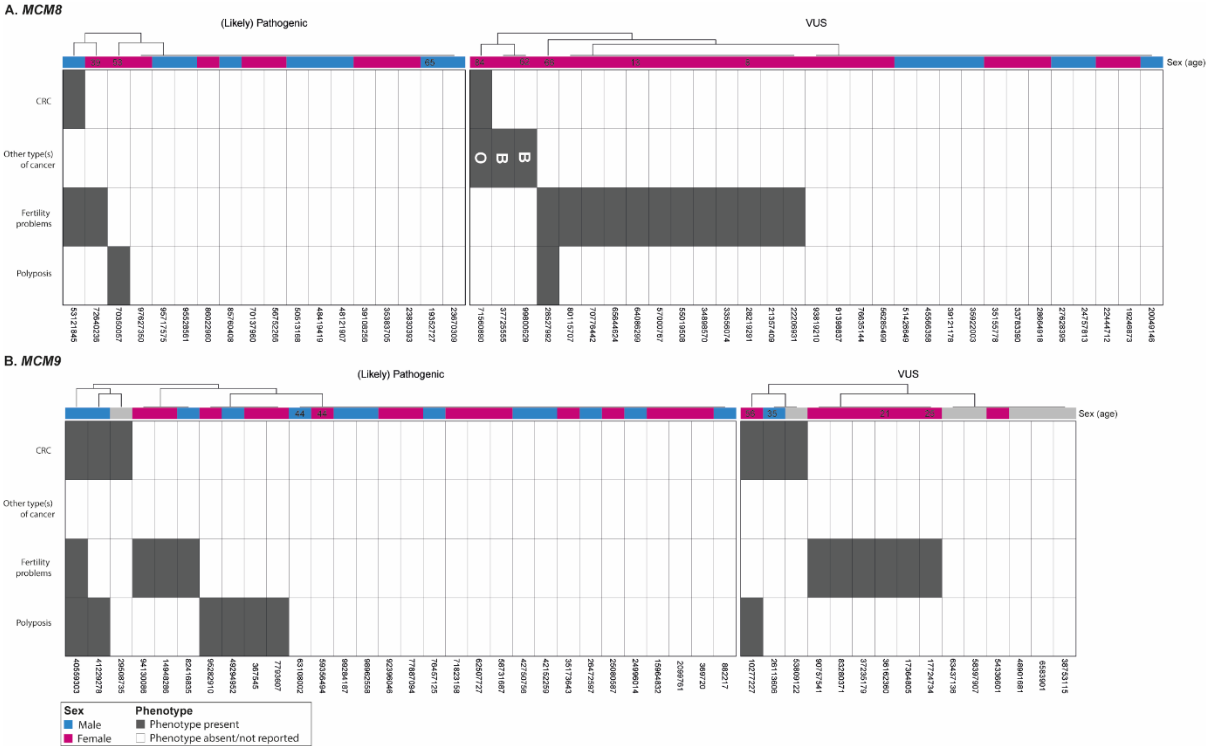

Figure S2. Phenotype of monoallelic *MCM8/MCM9* variant carriers. The phenotype is presented for all (**A**) monoallelic *MCM8* and (**B**) monoallelic *MCM9* variant carriers from our case series. Each column represents an individual, while each row corresponds to one of the four primary observed phenotypes: CRC, other type(s) of cancer, hypogonadism, and polyposis. Person IDs are provided below each column, whereas their corresponding ages, which represent the most recent reported age of each individual, are shown above every column (when available). *B, breast cancer; CRC, colorectal cancer; O, ovarian cancer; VUS, variant of uncertain significance.*

Figure S3

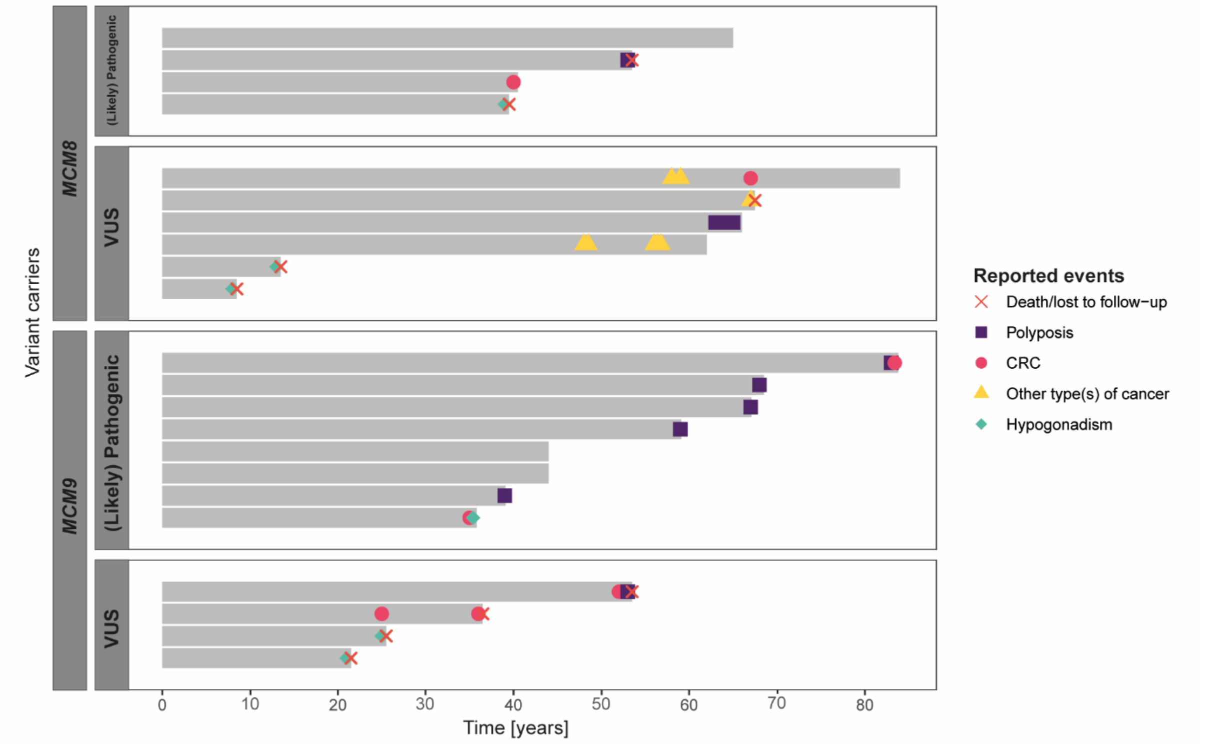

Figure S3. Disease onset in monoallelic *MCM8/MCM9* variant carriers. The onset of the four primary observed phenotypes (CRC, other type(s) of cancer, hypogonadism, and polyposis) is displayed for each monoallelic *MCM8/MCM9* variant carrier with available age details in our case series. Those without age details were excluded from the analysis. Individuals are ordered by ACMG/AMP classification (pathogenic or likely pathogenic, VUS)[55, 56] and current age or age at the time of death/lost to follow-up. *ACMG, American College of Medical Genetics and Genomics; AMP, Association for Molecular Pathology; CRC, colorectal cancer; VUS, variant of uncertain significance.*

Figure S4

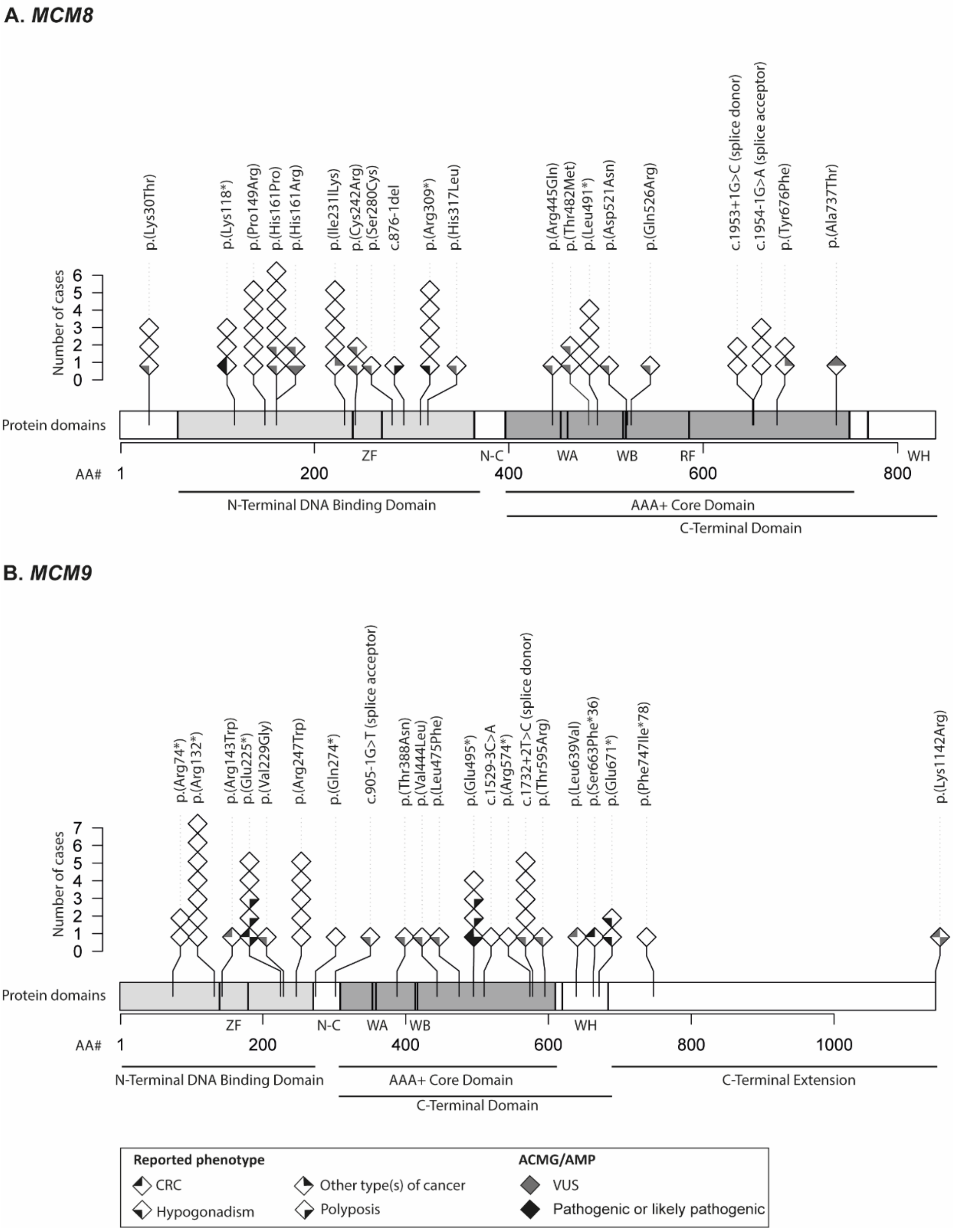

Figure S4. Monoallelic *MCM8/MCM9* variants mapped onto the respective protein domains. (**A**) A*MCM8* and (**B**) *MCM9* variants from all monoallelic variant carriers in our case series are mapped onto the domains of the MCM8 and MCM9 proteins, respectively. The fill and color of the diamond symbols correspond to the phenotype of the individual (CRC, other type(s) of cancer, hypogonadism, polyposis) and the ACMG/AMP classification of the variant (pathogenic or likely pathogenic, VUS)[55, 56], respectively. *ACMG, American College of Medical Genetics and Genomics; AMP, Association for*

Molecular Pathology; CRC, colorectal cancer; RF, arginine finger; VUS, variant of uncertain significance; WA, Walker A; WB, Walker B; WH, winged-helix; ZF, zinc-finger.

Figure S5

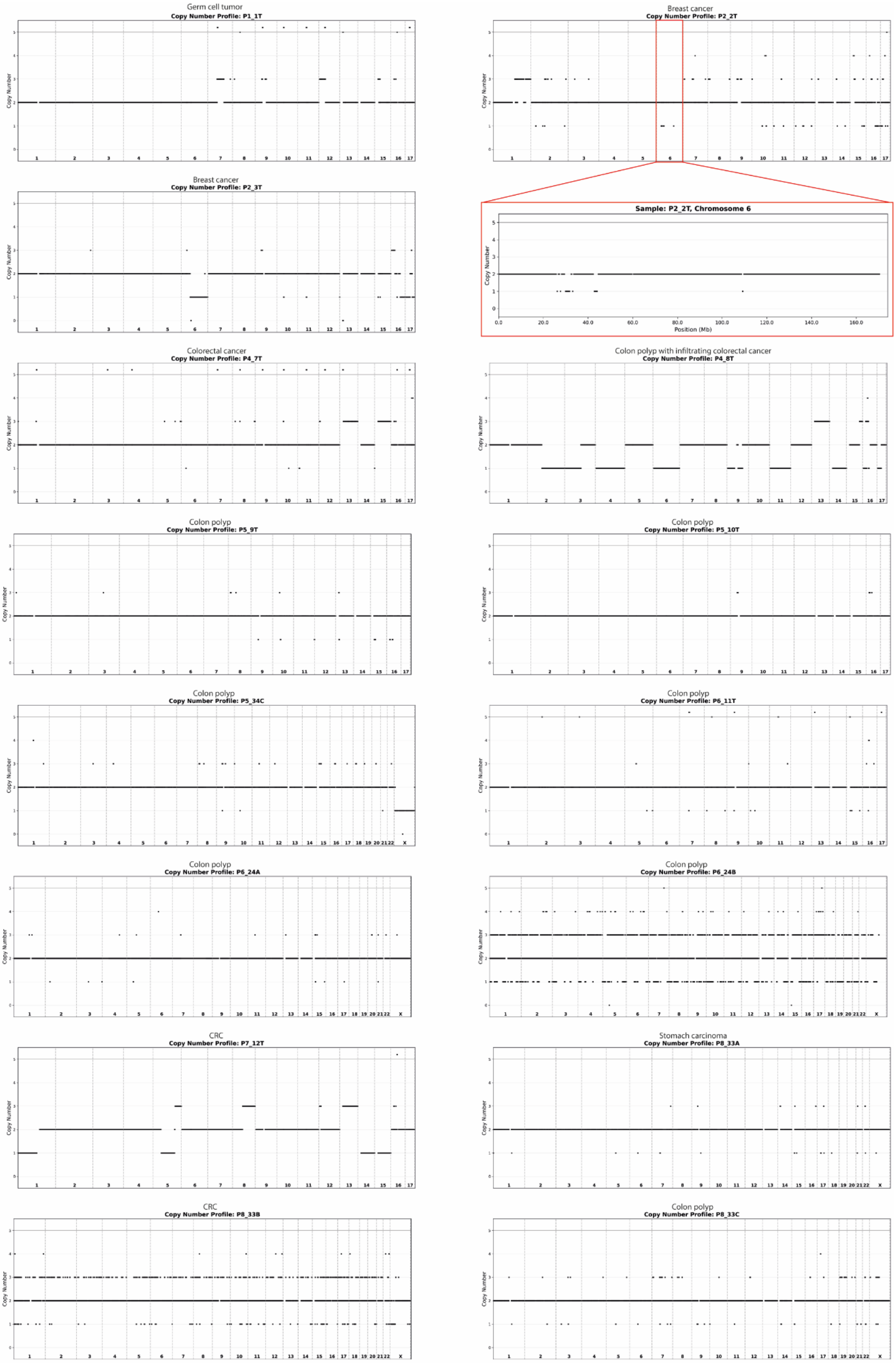

Germ cell tumor
Copy Number Profile: P1_1T
Breast cancer
Copy Number Profile: P2_2T
Breast cancer
Copy Number Profile: P2_3T
Sample: P2_2T, Chromosome 6
Position (Mb)
Colorectal cancer
Copy Number Profile: P4_7T
Colon polyp with infiltrating colorectal cancer
Copy Number Profile: P4_8T
Colon polyp
Copy Number Profile: P5_9T
Colon polyp
Copy Number Profile: P5_10T
Colon polyp
Copy Number Profile: P5_34C
Colon polyp
Copy Number Profile: P6_11T
Colon polyp
Copy Number Profile: P6_24A
Colon polyp
Copy Number Profile: P6_24B
CRC
Copy Number Profile: P7_12T
Stomach carcinoma
Copy Number Profile: P8_33A
CRC
Copy Number Profile: P8_33B
Colon polyp
Copy Number Profile: P8_33C

Figure S5. Copy number analysis of tumors from *MCM8/MCM9* variant carriers from our case series. Total copy number analysis was performed using CNVkit (v0.9.8) on WGS and WES data, processed separately. To improve visual clarity, segments smaller than 100 kb were excluded. Due to figure size constraints, some segments may appear to display two copy number values; however, this is neither a technical error nor artifact, as verified by the individual chromosome plots. A zoom-in of chromosome 6 from P2_2T (red box) illustrates that segments do not overlap. For enhanced readability, segments with copy number values above 5 are displayed above the grey reference bar. *WES, whole-exome sequencing; WGS, whole-genome sequencing.*

Figure S6

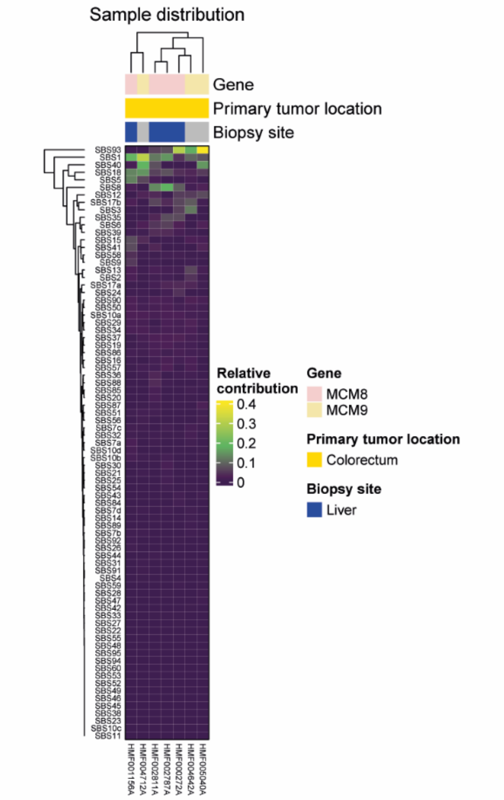

Figure S6. Mutational signature analysis of metastasized CRCs from monoallelic *MCM8/MCM9* variant carriers in the HMF cancer-specific cohort. Mutational signature analysis was conducted on metastasized CRCs from monoallelic *MCM8/MCM9* variant carriers in the HMF cancer-specific cohort. The heatmap displays unsupervised hierarchical clustering of the SBS mutational signature profiles. This analysis included tumors from four monoallelic *MCM8* and three monoallelic *MCM9* variant carriers without a second hit in the *MCM8/MCM9* genes. All germline *MCM8/MCM9* variants were classified as VUS per the ACMG/AMP classification for variant interpretation.[55, 56] The rows represent SBS mutational signatures, while the columns represent individual samples. The identification of SBS mutational signatures was achieved by fitting the counts of SNVs per 96 tri-nucleotide context to the COSMIC signatures[76], employing the MutationalPatterns tool.[77] *ACMG, American College of Medical Genetics and Genomics; AMP, Association for Molecular Pathology; HMF, Hartwig Medical Foundation; SBS, single base substitution; SNV, single nucleotide variant; VUS, variant of uncertain significance.*

Figure S7

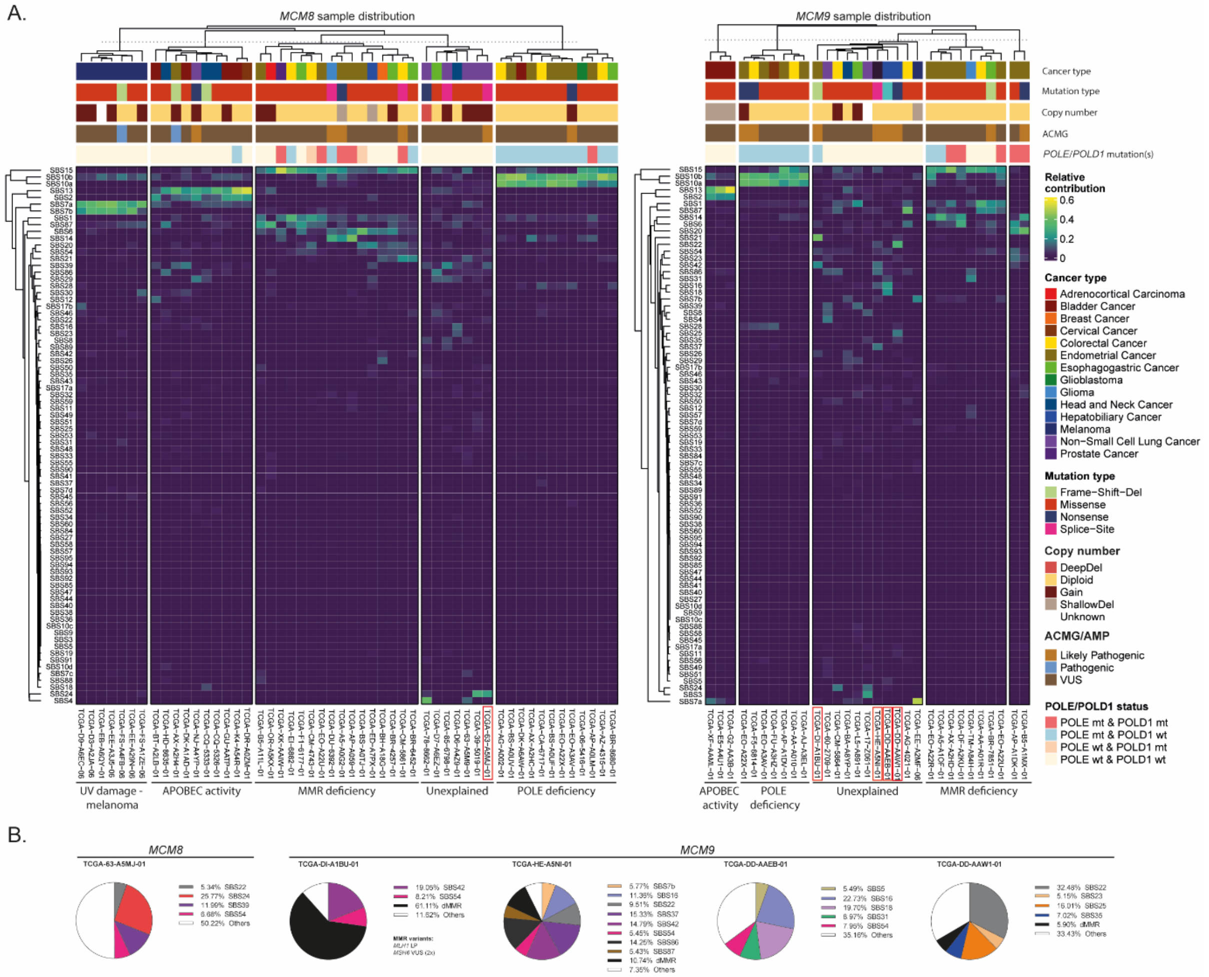

Figure S7. Mutational signature analysis on TCGA Pan-Cancer Atlas samples harboring somatic *MCM8/MCM9* mutation(s). (**A**) Heatmaps showing unsupervised hierarchic clustering of the SBS mutational signature profiles of TCGA Pan-Cancer Atlas tumors with somatic *MCM8/MCM9* mutation(s). Rows represent SBS mutational signatures, while columns represent individual samples. In clusters represented by SBS7a/b (UV damage), SBS2 and SBS13 (APOBEC activity), SBS6, SBS14, SBS15, SBS20, and SBS21 (MMR deficiency), or SBS10a/b (POLE deficiency), the somatic *MCM8/MCM9* variants were likely secondary to other mutational processes.[71, 72, 76, 85, 86] Tumors with likely pathogenic *MCM8* (n=1) or *MCM9* (n=4) variants in the unexplained clusters were marked by red squares. (**B**) In tumors with likely pathogenic *MCM8* (n=1) or *MCM9* (n=4) variants from the unexplained clusters, shared SBS mutational signatures included SBS22 (aristolochic acid exposure), SBS42 (haloalkane exposure), and SBS54 (sequencing artefact). Notably, SBS mutational signatures associated with MMR deficiency (SBS6, SBS14, SBS15, SBS20, SBS21) were also present in three of these five tumors. However, these could be explained by a likely pathogenic *MLH1* variant and two variants of unknown significance in the *MSH6* gene in one (TCGA-DI-A1BU-01) of these tumors. The TCGA Pan-Cancer Atlas was assessed via cBioPortal for Cancer Genomics (https://www.cbioportal.org/) in February-April, 2023. *ACMG, American College of Medical Genetics and Genomics; AMP, Association for Molecular Pathology; LP, likely pathogenic; mt, mutation; SBS, single base substitution; TCGA, The Cancer Genome Atlas; VUS, variant of uncertain significance; wt, wildtype.*

Supplemental tables

Table S1. ICD10 and ICD-O codes and corresponding phenotypes used to identify cohorts for variant enrichment analysis

ICD10 codes	Phenotype	100K Genomes Project		200K UK Biobank	
		n[a]	Median age (range)[b]	n[a]	Median age (range)[b]
K635	Colonic polyps	3051	70 (5-100)	9262	73 (45-84)
K621	Rectal polyps	1404	69 (5-98)	5164	72 (51-84)
D120,D121,D123,D124,D125,D126,D127,D128,D129	Colorectal adenomas	2877	72 (10-99)	10297	73 (45-84)
C180,C181,C182,C183,C184,C185,C186,C187,C188,C189,C19,C20	Colorectal cancer	3473	73 (17-102	3239	74 (42-84)
C509	Breast cancer	4438	66 (22-102)	6783	
C160, C161, C162, C163, C164, C165, C166, C168, C169	Gastric cancer	281	70 (18-97)	481	
C430, C431, C432, C433, C434, C435, C436, C437, C438, C439	Melanoma	714	70 (13-102)	1626	
C541, C542, C543, C548, C549, C55	Endometrial cancer	1107	71 (21-99)	938	
C56, C561, C562,C563, C569	Ovarian cancer	844		696	
C530, C531, C538, C539	Cervical cancer	135		145	
N979	Female infertility	434		495	57 (46-72)
E283	Primary Ovarian Insufficiency	71		24	
N46	Male infertility	20		33	
G400	Localization-related (focal) (partial) idiopathic epilepsy and epileptic syndromes with seizures of localized onset	218		25	
G401	Localization-related (focal) (partial) symptomatic epilepsy and epileptic syndromes with simple partial seizures	1413		124	
G402	Localization-related (focal) (partial) symptomatic epilepsy and epileptic syndromes with complex partial seizures	996	27 (0-89)	193	
G403	Generalized idiopathic epilepsy and epileptic syndromes	2251		313	
G404	Other generalized epilepsy and epileptic syndromes	724		8	
G405	Special epileptic syndromes	81		31	
E343	Short Stature	994		9	

E300	Delayed Puberty	129		0	
E039	Hypothyroidism	3323	63 (0-102)	10997	73 (44-84)
Q510	Absent/Infantile Uteri	13		0	
**ICD-O**	**Phenotype**	**100K Genomes Project**		**200K UK Biobank**	
		**n**[a]	**Median age (range)**[b]	**n**[a]	**Median age (range)**[b]
9061/3<br>9070/3<br>9071/3<br>9100/3<br>9080/0<br>9080/1<br>9085/3<br>9084/3<br>9086/3	Seminoma<br>Embryonal carcinoma<br>Yolk sac tumor<br>Choriocarcinoma<br>Mature teratoma<br>Immature teratoma of the yhymus<br>Mixed germ cell tumor<br>Teratoma with somatic type malignancies<br>Germ cell tumor with associated hematological malignancy	152		216	

[a] n = number of participants with each phenotype. Some participants had multiple ICD10 codes that were included in our search.
[b] The ages of the cases are provided for analyses where variant enrichment could be performed (i.e., at least one homozygous or compound heterozygous case and control was available).

ICD10, International Classification of Diseases 10th Revision; ICD-O, International Classification of Diseases for Oncology

Table S2. Overview of *MCM8/MCM9* variant carriers meeting pathogenicity-based filtering criteria, including sources

Current study ID	Current family ID (only for new cases from outpatient clinic or carriers from literature with updated data)	Source	Digital Object Identifier (DOI) of source (if applicable)	Study ID used in source (if applicable)	Germline *MCM8* variant(s)	Germline *MCM9* variant(s)
**Biallelic *MCM8* (VUS)**						
01143258		Carriers from literature - data from papers only	10.1016/j.fertnstert.2017.07.015	V-11	c.482A>C, p.(His161Pro)	
02328912	*MCM8*_04	Carriers from literature - updated data	10.1172/jci.insight.140698	SXS48	c.692T>A, p.(Ile231Lys); c.994A>G, p.(Thr332Ala)	
07868858		Carriers from literature - data from papers only	10.1016/j.ajhg.2022.01.011	P0281	c.482A>C, p.(His161Pro)	
15517012	*MCM8*_04	Outpatient clinic			c.692T>A, p.(Ile231Lys); c.994A>G, p.(Thr332Ala)	
28590785		Carriers from literature - data from papers only	10.1016/j.fertnstert.2017.07.015	V-5	c.482A>C, p.(His161Pro)	
39848302		Carriers from literature - data from papers only	10.1016/j.fertnstert.2017.07.015	V-9	c.482A>C, p.(His161Pro)	
48582770		Carriers from literature - data from papers only	10.1172/JCI78473	IV-9	c.446C>G, p.(Pro149Arg)	
55456091		Carriers from literature - data from papers only	10.1172/JCI78473	IV-6	c.446C>G, p.(Pro149Arg)	
71809414		Carriers from literature - data from papers only	10.1016/j.fertnstert.2017.07.015	V-10	c.482A>C, p.(His161Pro)	
92426276		Carriers from literature - data from papers only	10.1172/JCI78473	IV-1	c.446C>G, p.(Pro149Arg)	
96184605		Carriers from literature - data from papers only	10.1016/j.ajhg.2022.01.011	P0370	c.482A>C, p.(His161Pro)	
**Biallelic *MCM8* (pathogenic or likely pathogenic)**						
01130852		Carriers from literature - data from papers only	10.1210/jc.2019-00248	BAB7675	c.925C>T, p.(Arg309*)	
08232281		Carriers from literature - data from papers only	10.1136/jmedgenet-2014-102921	IV-6	c.1470_1471insTA, p.(Leu491fs)	
13147827		Carriers from literature - data from papers only	10.1002/mgg3.1165	IV-1	c.351_354del, p.(Lys118fs)	
23867510		Carriers from literature - data from papers only	10.1136/jmedgenet-2014-102921	V-1	c.1954-1G>A, splice acceptor	
25350218		Carriers from literature - data from papers only	10.1002/mgg3.1165	IV-3	c.351_354del, p.(Lys118fs)	
26298807	*MCM8*_01	Outpatient clinic			c.2027A>T, p.(Tyr676Phe); c.1953+1G>C, splice donor	

38595546		Carriers from literature - data from papers only	10.1136/jmedgenet-2014-102921	V-2	c.1954-1G>A, splice acceptor	
43675980		Carriers from literature - data from papers only	10.1136/jmedgenet-2014-102921	IV-3	c.1470_1471insTA, p.(Leu491fs)	
45685855		Carriers from literature - data from papers only	10.1210/clinem/dgaa155	IV-2	c.925C>T, p.(Arg309*)	
46951971		Carriers from literature - data from papers only	10.1210/jc.2019-00248	BAP7675	c.925C>T, p.(Arg309*)	
54907964		Carriers from literature - data from papers only	10.1136/jmedgenet-2014-102921	IV-2	c.1470_1471insTA, p.(Leu491fs)	
57322856		Carriers from literature - data from papers only	10.1136/jmedgenet-2014-102921	IV-7	c.1470_1471insTA, p.(Leu491fs)	
74081581		Carriers from literature - data from papers only	10.1136/jmedgenet-2014-102921	IV-4	c.1470_1471insTA, p.(Leu491fs)	
80565678		Carriers from literature - data from papers only	10.1038/s41431-021-00977-9	1	c.1953+1G>C, splice donor	
82585307	*MCM8*_01	Outpatient clinic			c.2027A>T, p.(Tyr676Phe); c.1953+1G>C, splice donor	
**Biallelic *MCM9* (VUS)**						
31857966		Carriers from literature - data from papers only	10.1093/hmg/ddaa101	NOA-144		c.1301A>C, p.(Gln434Pro)
57170234		Carriers from literature - data from papers only	10.1007/s10815-021-02083-7	FS0054		c.1291A>G, p.(Met431Val); c.1157C>T, p.(Thr386Met)
58289086		Carriers from literature - data from papers only	10.1007/s10815-018-1349-4	FPOI38		c.1784C>G, p.(Thr595Arg); c.905-1G>T, splice acceptor
78493209	*MCM9*_09	Outpatient clinic				c.1642C>T, p.(Arg548Trp); c.152A>T, p.(Asn51Ile)
82082774	*MCM9*_09	Carriers from literature - updated data	10.1172/jci.insight.140698	011-69294-1		c.1642C>T, p.(Arg548Trp); c.152A>T, p.(Asn51Ile)
88176711	*MCM9*_08	Carriers from literature - updated data	10.1172/jci.insight.140698	MSS13-1961		c.3425A>G, p.(Lys1142Arg); c.1640T>C, p.(Leu547Pro)
**Biallelic *MCM9* (pathogenic or likely pathogenic)**						
08014933	*MCM9*_03	Carriers from literature - updated data	10.1016/j.cancergen.2015.10.001	III-4		c.672_673delinsC, p.(Glu225fs)
17276581	*MCM9*_04	Carriers from literature - updated data	10.1038/s41525-021-00242-4	IV-2		c.1483G>T, p.(Glu495*)
17419906		Carriers from literature - data from papers only	10.1515/jpem-2020-0590	P33		c.1732+2T>C, splice donor
30893196	*MCM9*_05	Carriers from literature - updated data	10.3390/jcm12030990	IV-2		c.394C>T, p.(Arg132*)
37139385		Carriers from literature - data from papers only	10.1016/j.ajhg.2014.11.002	AII-6		c.1732+2T>C, splice donor
40636194		Carriers from literature - data from papers only	10.1210/jc.2019-00248	BAB10068		c.220C>T, p.(Arg74*)
42545284		Carriers from literature - data from papers only	10.1515/jpem-2020-0590	P32		c.1732+2T>C, splice donor

44283624	*MCM9*_04	Carriers from literature - updated data	10.1038/s41525-021-00242-4	IV-1		c.1483G>T, p.(Glu495*)
54365488	*MCM9*_06	Outpatient clinic				c.1720C>T, p.(Arg574*); c.1529-3C>A, splice donor
54601491		Carriers from literature - data from papers only	10.1111/cge.13803	POI-02		c.1473dup, p.(Thr492Tyrfs*4)
54840711	*MCM9*_05	Carriers from literature - updated data	10.3390/jcm12030990	IV-3		c.394C>T, p.(Arg132*)
67217467		Carriers from literature - data from papers only	10.1007/s10815-018-1349-4	FPOI24		c.1651C>T, p.(Gln551*)
69061260		Carriers from literature - data from papers only	10.1111/cge.13803	POI-03		c.1473dup, p.(Thr492Tyrfs*4)
74185426	*MCM9*_05	Carriers from literature - updated data	10.3390/jcm12030990	IV-5		c.394C>T, p.(Arg132*)
75503761		Carriers from literature - data from papers only	10.1210/jc.2019-00248	BAB9435		c.394C>T, p.(Arg132*)
79778808		Carriers from literature - data from papers only	10.1210/jc.2016-2565			c.1651C>T, p.(Gln551*)
80899809	*MCM9*_05	Carriers from literature - updated data	10.3390/jcm12030990	IV-1		c.394C>T, p.(Arg132*)
80909502		Carriers from literature - data from papers only	10.1016/j.ajhg.2014.11.002	AII-4		c.1732+2T>C, splice donor
82941667		Carriers from literature - data from papers only	10.1016/j.ajhg.2014.11.002	BII-1		c.394C>T, p.(Arg132*)
91725927	*MCM9*_10	Outpatient clinic				c.394C>T, p.(Arg132*)
98796144	*MCM9*_03	Carriers from literature - updated data	10.1016/j.cancergen.2015.10.001	III-3		c.672_673delinsC, p.(Glu225fs)
17290073	*MCM9*_07	Outpatient clinic				c.820C>T, p.(Gln274*); c.2237_2238dup, p.(Phe747Ilefs*78)
**Monoallelic *MCM8* (VUS)**						
19246873	*MCM8*_04	Outpatient clinic			c.692T>A, p.(Ile231Lys)	
20049146		Carriers from literature - data from papers only	10.1172/JCI78473	IV-8	c.446C>G, p.(Pro149Arg)	
21357409		Carriers from literature - data from papers only	10.1186/s12920-020-00813-x	P32	c.839C>G, p.(Ser280Cys)	
22206931		Carriers from literature - data from papers only	10.1210/jc.2019-00248	BAP7100	c.89A>C, p.(Lys30Thr); c.1330A>G, p.(Ile444Val)	
22444712		Carriers from literature - data from papers only	10.1210/jc.2019-00248		c.89A>C, p.(Lys30Thr)	
24757813		Carriers from literature - data from papers only	10.1016/j.fertnstert.2017.07.015	IV-3	c.482A>C, p.(His161Pro)	
27628395		Carriers from literature - data from papers only	10.1172/JCI78473	IV-2	c.446C>G, p.(Pro149Arg)	
28219291		Carriers from literature - data from papers only	10.1002/mgg3.1396	Proband's sister	c.724T>C, p.(Cys242Arg); c.1334C>A, p.(Ala445Asp)	

28527992	*MCM8*_03	Outpatient clinic			c.482A>G, p.(His161Arg)	
28664918		Carriers from literature - data from papers only	10.1172/JCI78473	IV-5	c.446C>G, p.(Pro149Arg)	
33556074		Carriers from literature - data from papers only	10.1186/s12920-020-00813-x	P28	c.1445C>T, p.(Thr482Met)	
33783390	*MCM8*_04	Outpatient clinic			c.692T>A, p.(Ile231Lys)	
34898570		Carriers from literature - data from papers only	10.1210/jc.2016-2565		c.1577A>G, p.(Gln526Arg)	
35155778	*MCM8*_01	Outpatient clinic			c.2027A>T, p.(Tyr676Phe)	
35922003		Carriers from literature - data from papers only	10.1016/j.fertnstert.2017.07.015	V-8	c.482A>C, p.(His161Pro)	
37725555	*MCM8*_04	Outpatient clinic			c.692T>A, p.(Ile231Lys)	
39121178	*MCM8*_04	Outpatient clinic			c.692T>A, p.(Ile231Lys)	
45566358		Carriers from literature - data from papers only	10.1172/JCI78473	III-1	c.446C>G, p.(Pro149Arg)	
51426649		Carriers from literature - data from papers only	10.1210/jc.2019-00248		c.89A>C, p.(Lys30Thr); c.1330A>G, p.(Ile444Val)	
55019508		Carriers from literature - data from papers only	10.1016/j.fertnstert.2016.08.018	192	c.950A>T, p.(His317Leu); c.1801_1803del, p.(His601Arg)	
56285499		Carriers from literature - data from papers only	10.1016/j.fertnstert.2017.07.015	V-6	c.482A>C, p.(His161Pro)	
57000767		Carriers from literature - data from papers only	10.1210/jc.2016-2565		c.1561G>A, p.(Asp521Asn)	
64086299		Carriers from literature - data from papers only	10.1002/mgg3.1396	Proband	c.724T>C, p.(Cys242Arg); c.1334C>A, p.(Ala445Asp)	
65644624		Carriers from literature - data from papers only	10.1210/jc.2016-2565		c.482A>G, p.(His161Arg)	
70776442		Carriers from literature - data from papers only	10.1186/s12920-020-00813-x	P14	c.1445C>T, p.(Thr482Met)	
71560890	*MCM8*_02	Carriers from literature - updated data	10.1172/jci.insight.140698	MSS23-1939	c.2209G>A, p.(Ala737Thr)	
76635144		Carriers from literature - data from papers only	10.1016/j.fertnstert.2017.07.015	IV-4	c.482A>C, p.(His161Pro)	
80115707		Carriers from literature - data from papers only	10.1210/jc.2016-2565		c.1334G>A, p.(Arg445Gln)	
91398837		Carriers from literature - data from papers only	10.1172/JCI78473	III-2	c.446C>G, p.(Pro149Arg)	
93819210	*MCM8*_04	Outpatient clinic			c.692T>A, p.(Ile231Lys)	
99800629	*MCM8*_01	Outpatient clinic			c.2027A>T, p.(Tyr676Phe)	
**Monoallelic *MCM8* (pathogenic or likely pathogenic)**						

19352727	*MCM8*_01	Outpatient clinic			c.1953+1G>C, splice donor	
23670309		Carriers from literature - data from papers only	10.1002/mgg3.1165	III-1	c.351_354del, p.(Lys118fs)	
23830393		Carriers from literature - data from papers only	10.1136/jmedgenet-2014-102921	III-1	c.1470_1471insTA, p.(Leu491fs)	
35383705		Carriers from literature - data from papers only	10.1136/jmedgenet-2014-102921	IV-1	c.1954-1G>A, splice acceptor	
39108256		Carriers from literature - data from papers only	10.1210/jc.2019-00248		c.925C>T, p.(Arg309*)	
48121907		Carriers from literature - data from papers only	10.1136/jmedgenet-2014-102921	IV-5	c.1470_1471insTA, p.(Leu491fs)	
48419419		Carriers from literature - data from papers only	10.1136/jmedgenet-2014-102921	V-3	c.1954-1G>A, splice acceptor	
50513168		Carriers from literature - data from papers only	10.1210/jc.2019-00248		c.925C>T, p.(Arg309*)	
53121845	*MCM8*_05	Carriers from literature - updated data	10.1172/jci.insight.140698	LLS17	c.351_354del, p.(Lys118Glufs*5); c.414A>G, p.(Ile138Met)	
56752266		Carriers from literature - data from papers only	10.1002/mgg3.1165	III-2	c.351_354del, p.(Lys118fs)	
70137960		Carriers from literature - data from papers only	10.1136/jmedgenet-2014-102921	III-3	c.1470_1471insTA, p.(Leu491fs)	
70350057		Carriers from literature - data from papers only	10.3390/cancers13040929	AA3530	c.876-1del	
72640236		Carriers from literature - data from papers only	10.1210/clinem/dgaa155	III-2	c.925C>T, p.(Arg309*)	
85760408		Carriers from literature - data from papers only	10.1210/clinem/dgaa155	III-1	c.925C>T, p.(Arg309*)	
86022960	*MCM8*_01	Outpatient clinic			c.1953+1G>C, splice donor	
95528561		Carriers from literature - data from papers only	10.1136/jmedgenet-2014-102921	III-2	c.1470_1471insTA, p.(Leu491fs)	
95717575		Carriers from literature - data from papers only	10.1136/jmedgenet-2014-102921	IV-2	c.1954-1G>A, splice acceptor	
97627350		Carriers from literature - data from papers only	10.1210/clinem/dgaa155	IV-1	c.925C>T, p.(Arg309*)	
**Monoallelic *MCM9* (VUS)**						
06583901		Carriers from literature - data from papers only	10.1016/j.neurobiolaging.2021.12.004	III-4		c.739C>T, p.(Arg247Trp)
10277227	*MCM8_MCM9_*01	Carriers from literature - updated data	10.1172/jci.insight.140698	MSS2-1941	c.832C>T, p.(Arg278Cys)	c.3425A>G, p.(Lys1142Arg)
17364805		Carriers from literature - data from papers only	10.1210/jc.2016-2565			c.686T>G, p.(Val229Gly)
17724734		Carriers from literature - data from papers only	10.1016/j.fertnstert.2019.11.015	POI-1		c.1423C>T, p.(Leu475Phe)

26113606	*MCM9*_02	Carriers from literature - updated data	10.1172/jci.insight.140698	NA96-14		c.1915C>G, p.(Leu639Val)
36162360		Carriers from literature - data from papers only	10.1371/journal.pone.0240795	POI-25		c.1163C>A, p.(Thr388Asn)
37235179		Carriers from literature - data from papers only	10.1210/jc.2016-2565			c.1784C>G, p.(Thr595Arg)
38753115		Carriers from literature - data from papers only	10.1016/j.neurobiolaging.2021.12.004	IV-9		c.739C>T, p.(Arg247Trp)
48901681		Carriers from literature - data from papers only	10.1016/j.neurobiolaging.2021.12.004	III-8		c.739C>T, p.(Arg247Trp)
53809122		Carriers from literature - data from papers only	10.1002/humu.24057	F39		c.427C>T, p.(Arg143Trp)
54336601	*MCM9*_06	Outpatient clinic				c.1529-3C>A, splice donor
58397907		Carriers from literature - data from papers only	10.1016/j.neurobiolaging.2021.12.004	III-3		c.739C>T, p.(Arg247Trp)
63437136		Carriers from literature - data from papers only	10.1016/j.neurobiolaging.2021.12.004	III-1		c.739C>T, p.(Arg247Trp)
83280371		Carriers from literature - data from papers only	10.1186/s12920-020-00813-x	P26		c.1330G>C, p.(Val444Leu)
90757541		Carriers from literature - data from papers only	10.1210/jc.2016-2565			c.905-1G>T, splice acceptor
**Monoallelic *MCM9* (pathogenic or likely pathogenic)**						
00367545	*MCM9*_03	Carriers from literature - updated data	10.1016/j.cancergen.2015.10.001	III-2		c.672_673delinsC, p.(Glu225fs)
00369720		Carriers from literature - data from papers only	10.1515/jpem-2020-0590			c.1732+2T>C, splice donor
00882217		Carriers from literature - data from papers only	10.1016/j.ajhg.2014.11.002	AI-1		c.1732+2T>C, splice donor
02099761		Carriers from literature - data from papers only	10.1016/j.ajhg.2014.11.002	AI-2		c.1732+2T>C, splice donor
07793607	*MCM9*_03	Carriers from literature - updated data	10.1016/j.cancergen.2015.10.001	II-1		c.672_673delinsC, p.(Glu225fs)
14948286		Carriers from literature - data from papers only	10.1210/jc.2016-2565			c.2011G>T, p.(Glu671*)
15964832		Carriers from literature - data from papers only	10.1210/jc.2019-00248			c.220C>T, p.(Arg74*)
24996014		Carriers from literature - data from papers only	10.1016/j.ajhg.2014.11.002	BII-2		c.394C>T, p.(Arg132*)
25080587	*MCM9*_03	Carriers from literature - updated data	10.1016/j.cancergen.2015.10.001	III-1		c.672_673delinsC, p.(Glu225fs)
26472597		Carriers from literature - data from papers only	10.1016/j.ajhg.2014.11.002	BI-1		c.394C>T, p.(Arg132*)
29508735	*MCM9*_01	Carriers from literature - updated data	10.1172/jci.insight.140698	NA41-1		c.1987dup, p.(Ser663Phefs*36)
35173643		Carriers from literature - data from papers only	10.1016/j.ajhg.2014.11.002	AII-3		c.1732+2T>C, splice donor

40559303	*MCM9*_04	Carriers from literature - updated data	10.1038/s41525-021-00242-4	IV-3		c.1483G>T, p.(Glu495*)
41229278	*MCM9*_03	Carriers from literature - updated data	10.1016/j.cancergen.2015.10.001	II-2		c.672_673delinsC, p.(Glu225fs)
42152259		Carriers from literature - data from papers only	10.1210/jc.2019-00248			c.220C>T, p.(Arg74*)
42750756		Carriers from literature - data from papers only	10.1210/jc.2019-00248			c.394C>T, p.(Arg132*)
49294952	*MCM9*_04	Carriers from literature - updated data	10.1038/s41525-021-00242-4	III-2		c.1483G>T, p.(Glu495*)
58731687		Carriers from literature - data from papers only	10.1016/j.ajhg.2014.11.002	AII-2		c.1732+2T>C, splice donor
62507727		Carriers from literature - data from papers only	10.1210/jc.2019-00248			c.394C>T, p.(Arg132*)
71823158	*MCM9*_03	Carriers from literature - updated data	10.1016/j.cancergen.2015.10.001	I-1		c.672_673delGGinsC, p.(Glu225fs)
76457125		Carriers from literature - data from papers only	10.1016/j.ajhg.2014.11.002	BII-4		c.394C>T, p.(Arg132*)
77887094		Carriers from literature - data from papers only	10.1016/j.ajhg.2014.11.002	BI-2		c.394C>T, p.(Arg132*)
82416835		Carriers from literature - data from papers only	10.1515/jpem-2020-0590			c.1732+2T>C, splice donor
92396046	*MCM9*_04	Carriers from literature - updated data	10.1038/s41525-021-00242-4	IV-4		c.1483G>T, p.(Glu495*)
94130086		Carriers from literature - data from papers only	10.1210/jc.2016-2565			c.2011G>T, p.(Glu671*)
95282910	*MCM9*_04	Carriers from literature - updated data	10.1038/s41525-021-00242-4	III-3		c.1483G>T, p.(Glu495*)
98962558	*MCM9*_05	Carriers from literature - updated data	10.3390/jcm12030990	IV-6		c.394C>T, p.(Arg132*)
99284187	*MCM9*_06	Outpatient clinic				c.1720C>T, p.(Arg574*)
59356494	*MCM9*_07	Outpatient clinic				c.820C>T, p.(Gln274*)
63108002	*MCM9*_07	Outpatient clinic				c.2237_2238dup, p.(Phe747Ilefs*78)

Table S3. Driver mutations in CRC-related genes detected in tumors from our case series.

Sample	HUGO symbol	Driver mutation	Variant classification
P1_1T	*ARID1A*	c.5452C>T, p.(Gln1818*)	Nonsense mutation
	DICER1	c.1966C>T. p.(Arg656*)	Nonsense mutation
	EP300	-	Splice site
	GRIN2A	c.601C>T, p.(Gln201*)	Nonsense mutation
	LRP1B	c.5395C>T, p.(Gln1799*)	Nonsense mutation
	LRP1B	-	Splice site
	LRP1B	c.1453insA, p.(Gly485Trpfs*)	Frame shift ins
	MYH9	c.5014G>T, p.(Glu1672*)	Nonsense mutation
	SMARCA4	c.580C>T, p.(Gln194*)	Nonsense mutation
P2_2T	*CYLD*	c.686delT, p.(Pro229Leufs*)	Frame shift del
P4_7T	*CTNNB1*	c.110C>A, p.(Ser37Tyr)	Missense mutation
P4_8T	*CTNNB1*	c.110C>A, p.(Ser37Tyr)	Missense mutation
P4_8T	*KRAS*	c.35G>A, p.(Gly12Asp)	Missense mutation
P5_10T	*AMER1*	c.3329C>A, p.(Ser1110*)	Nonsense mutation
	FBXW7	c.1393C>T, p.(Arg465Cys)	Missense mutation
	ROBO2	c.727G>T, p.(Glu243*)	Nonsense mutation
	TP53	-	Splice site
P5_9T	*APC*	-	Splice site
	APC	c.2767A>T, p.(Arg923*)	Nonsense mutation
	LRP1B	c.6064G>T, p.(Gly2022*)	Nonsense mutation
P6_11T	*APC*	c.2674G>T, p.(Glu892*)	Nonsense mutation
	ROBO2	p.(Thr470Argfs*)	Frame shift del
P7_12T	*FBXW7*	c.37C>T, p.(Arg13*)	Nonsense mutation
	TP53	c.524G>A, p.(Arg175His)	Missense mutation
	TP53	-	Splice site

CRC, colorectal cancer

Supplemental references

1. Golubicki M, Bonjoch L, Acuna-Ochoa JG, et al. Germline biallelic Mcm8 variants are associated with early-onset Lynch-like syndrome. JCI Insight. 2020;5(18).
2. Goldberg Y, Halpern N, Hubert A, et al. Mutated MCM9 is associated with predisposition to hereditary mixed polyposis and colorectal cancer in addition to primary ovarian failure. Cancer Genet. 2015;208(12):621-4.
3. Goldberg Y, Aleme O, Peled-Perets L, et al. MCM9 is associated with germline predisposition to early-onset cancer-clinical evidence. NPJ Genom Med. 2021;6(1):78.
4. Potorac I, Laterre M, Malaise O, et al. The Role of MCM9 in the Etiology of Sertoli Cell-Only Syndrome and Premature Ovarian Insufficiency. J Clin Med. 2023;12(3).
5. Richards CS, Bale S, Bellissimo DB, et al. ACMG recommendations for standards for interpretation and reporting of sequence variations: Revisions 2007. Genet Med. 2008;10(4):294-300.
6. Richards S, Aziz N, Bale S, et al. Standards and guidelines for the interpretation of sequence variants: a joint consensus recommendation of the American College of Medical Genetics and Genomics and the Association for Molecular Pathology. Genet Med. 2015;17(5):405-24.
7. Wyrwoll MJ, Kockerling N, Vockel M, et al. Genetic Architecture of Azoospermia-Time to Advance the Standard of Care. Eur Urol. 2023;83(5):452-62.
